# Worldwide trends in hypertension prevalence and progress in treatment and control from 1990 to 2019: a pooled analysis of 1201 population-representative studies with 104 million participants

**DOI:** 10.1016/S0140-6736(21)01330-1

**Published:** 2021-09-11

**Authors:** NCD Risk Factor Collaboration (NCD-RisC), Bin Zhou, Rodrigo M Carrillo-Larco, Goodarz Danaei, Leanne M Riley, Christopher J Paciorek, Gretchen A Stevens, Edward W Gregg, James E Bennett, Bethlehem Solomon, Rosie K Singleton, Marisa K Sophiea, Maria LC Iurilli, Victor PF Lhoste, Melanie J Cowan, Stefan Savin, Mark Woodward, Yulia Balanova, Renata Cifkova, Albertino Damasceno, Paul Elliott, Farshad Farzadfar, Jiang He, Nayu Ikeda, Andre P Kengne, Young-Ho Khang, Hyeon Chang Kim, Avula Laxmaiah, Hsien-Ho Lin, Paula Margozzini Maira, J Jaime Miranda, Hannelore Neuhauser, Johan Sundström, Cherian Varghese, Indah S Widyahening, Tomasz Zdrojewski, Leandra Abarca-Gómez, Ziad A Abdeen, Hanan F Abdul Rahim, Niveen M Abu-Rmeileh, Benjamin Acosta-Cazares, Robert J Adams, Wichai Aekplakorn, Kaosar Afsana, Shoaib Afzal, Imelda A Agdeppa, Javad Aghazadeh-Attari, Carlos A Aguilar-Salinas, Charles Agyemang, Noor Ani Ahmad, Ali Ahmadi, Naser Ahmadi, Nastaran Ahmadi, Fariba Ahmadizar, Soheir H Ahmed, Wolfgang Ahrens, Kamel Ajlouni, Rajaa Al-Raddadi, Monira Alarouj, Fadia AlBuhairan, Shahla AlDhukair, Mohamed M Ali, Abdullah Alkandari, Ala'a Alkerwi, Kristine Allin, Eman Aly, Deepak N Amarapurkar, Norbert Amougou, Philippe Amouyel, Lars Bo Andersen, Sigmund A Anderssen, Ranjit Mohan Anjana, Alireza Ansari-Moghaddam, Daniel Ansong, Hajer Aounallah-Skhiri, Joana Araújo, Inger Ariansen, Tahir Aris, Raphael E Arku, Nimmathota Arlappa, Krishna K Aryal, Thor Aspelund, Felix K Assah, Maria Cecília F Assunção, Juha Auvinen, Mária Avdićová, Ana Azevedo, Mohsen Azimi-Nezhad, Fereidoun Azizi, Mehrdad Azmin, Bontha V Babu, Suhad Bahijri, Nagalla Balakrishna, Mohamed Bamoshmoosh, Maciej Banach, Maja Banadinović, Piotr Bandosz, José R Banegas, Joanna Baran, Carlo M Barbagallo, Alberto Barceló, Amina Barkat, Marta Barreto, Aluisio JD Barros, Mauro Virgílio Gomes Barros, Anna Bartosiewicz, Abdul Basit, Joao Luiz D Bastos, Iqbal Bata, Anwar M Batieha, Assembekov Batyrbek, Louise A Baur, Robert Beaglehole, Antonisamy Belavendra, Habiba Ben Romdhane, Mikhail Benet, Lowell S Benson, Salim Berkinbayev, Antonio Bernabe-Ortiz, Gailute Bernotiene, Heloísa Bettiol, Jorge Bezerra, Aroor Bhagyalaxmi, Santosh K Bhargava, Daniel Bia, Katia Biasch, Elysée Claude Bika Lele, Mukharram M Bikbov, Bihungum Bista, Peter Bjerregaard, Espen Bjertness, Marius B Bjertness, Cecilia Björkelund, Katia V Bloch, Anneke Blokstra, Simona Bo, Martin Bobak, Heiner Boeing, Jose G Boggia, Carlos P Boissonnet, Stig E Bojesen, Vanina Bongard, Alice Bonilla-Vargas, Matthias Bopp, Herman Borghs, Pascal Bovet, Christopher B Boyer, Lutgart Braeckman, Imperia Brajkovich, Francesco Branca, Juergen Breckenkamp, Hermann Brenner, Lizzy M Brewster, Yajaira Briceño, Miguel Brito, Graziella Bruno, H Bas Bueno-de-Mesquita, Gloria Bueno, Anna Bugge, Con Burns, Michael Bursztyn, Antonio Cabrera de León, Joseph Cacciottolo, Christine Cameron, Günay Can, Ana Paula C Cândido, Mario V Capanzana, Naděžda Čapková, Eduardo Capuano, Vincenzo Capuano, Viviane C Cardoso, Axel C Carlsson, Joana Carvalho, Felipe F Casanueva, Laura Censi, Marvin Cervantes-Loaiza, Charalambos A Chadjigeorgiou, Snehalatha Chamukuttan, Angelique W Chan, Queenie Chan, Himanshu K Chaturvedi, Nish Chaturvedi, Miao Li Chee, Chien-Jen Chen, Fangfang Chen, Huashuai Chen, Shuohua Chen, Zhengming Chen, Ching-Yu Cheng, Bahman Cheraghian, Imane Cherkaoui Dekkaki, Angela Chetrit, Kuo-Liong Chien, Arnaud Chiolero, Shu-Ti Chiou, Adela Chirita-Emandi, María-Dolores Chirlaque, Belong Cho, Kaare Christensen, Diego G Christofaro, Jerzy Chudek, Eliza Cinteza, Frank Claessens, Janine Clarke, Els Clays, Emmanuel Cohen, Hans Concin, Cyrus Cooper, Tara C Coppinger, Simona Costanzo, Dominique Cottel, Chris Cowell, Cora L Craig, Amelia C Crampin, Ana B Crujeiras, Juan J Cruz, Semánová Csilla, Liufu Cui, Felipe V Cureau, Sarah Cuschieri, Graziella D'Arrigo, Eleonora d'Orsi, Jean Dallongeville, Rachel Dankner, Thomas M Dantoft, Luc Dauchet, Kairat Davletov, Guy De Backer, Dirk De Bacquer, Amalia De Curtis, Giovanni de Gaetano, Stefaan De Henauw, Paula Duarte de Oliveira, David De Ridder, Delphine De Smedt, Mohan Deepa, Alexander D Deev, Vincent Jr DeGennaro, Hélène Delisle, Stefaan Demarest, Elaine Dennison, Valérie Deschamps, Meghnath Dhimal, Augusto F Di Castelnuovo, Juvenal Soares Dias-da-Costa, Alejandro Diaz, Ty T Dickerson, Zivka Dika, Shirin Djalalinia, Ha TP Do, Annette J Dobson, Chiara Donfrancesco, Silvana P Donoso, Angela Döring, Maria Dorobantu, Marcus Dörr, Kouamelan Doua, Nico Dragano, Wojciech Drygas, Charmaine A Duante, Priscilla Duboz, Rosemary B Duda, Virginija Dulskiene, Anar Dushpanova, Aleksandar Džakula, Vilnis Dzerve, Elzbieta Dziankowska-Zaborszczyk, Ricky Eddie, Ebrahim Eftekhar, Robert Eggertsen, Sareh Eghtesad, Gabriele Eiben, Ulf Ekelund, Mohammad El-Khateeb, Jalila El Ati, Denise Eldemire-Shearer, Marie Eliasen, Roberto Elosua, Rajiv T Erasmus, Raimund Erbel, Cihangir Erem, Louise Eriksen, Johan G Eriksson, Jorge Escobedo-de la Peña, Saeid Eslami, Ali Esmaeili, Alun Evans, David Faeh, Albina A Fakhretdinova, Caroline H Fall, Elnaz Faramarzi, Mojtaba Farjam, Mohammad Reza Fattahi, Asher Fawwad, Francisco J Felix-Redondo, Stephan B Felix, Trevor S Ferguson, Romulo A Fernandes, Daniel Fernández-Bergés, Daniel Ferrante, Thomas Ferrao, Marika Ferrari, Marco M Ferrario, Catterina Ferreccio, Haroldo S Ferreira, Eldridge Ferrer, Jean Ferrieres, Thamara Hubler Figueiró, Günther Fink, Krista Fischer, Leng Huat Foo, Maria Forsner, Heba M Fouad, Damian K Francis, Maria do Carmo Franco, Ruth Frikke-Schmidt, Guillermo Frontera, Flavio D Fuchs, Sandra C Fuchs, Yuki Fujita, Matsuda Fumihiko, Viktoriya Furdela, Ariel Furer, Takuro Furusawa, Zbigniew Gaciong, Andrzej Galbarczyk, Henrike Galenkamp, Fabio Galvano, Jingli Gao, Pei Gao, Manoli Garcia-de-la-Hera, Pablo Garcia, Dickman Gareta, Sarah P Garnett, Jean-Michel Gaspoz, Magda Gasull, Andrea Gazzinelli, Ulrike Gehring, Johanna M Geleijnse, Ronnie George, Ali Ghanbari, Erfan Ghasemi, Oana-Florentina Gheorghe-Fronea, Anup Ghimire, Alessandro Gialluisi, Simona Giampaoli, Christian Gieger, Tiffany K Gill, Jonathan Giovannelli, Glen Gironella, Aleksander Giwercman, Konstantinos Gkiouras, Marcel Goldberg, Rebecca A Goldsmith, Luis F Gomez, Aleksandra Gomula, Helen Gonçalves, Mauer Gonçalves, Bruna Gonçalves Cordeiro da Silva, David A Gonzalez-Chica, Marcela Gonzalez-Gross, Juan P González-Rivas, Clicerio González-Villalpando, María-Elena González-Villalpando, Angel R Gonzalez, Mariano Bonet Gorbea, Frederic Gottrand, Sidsel Graff-Iversen, Dušan Grafnetter, Aneta Grajda, Maria G Grammatikopoulou, Ronald D Gregor, Tomasz Grodzicki, Giuseppe Grosso, Gabriella Gruden, Dongfeng Gu, Ong Peng Guan, Elias F Gudmundsson, Vilmundur Gudnason, Ramiro Guerrero, Idris Guessous, Andre L Guimaraes, Martin C Gulliford, Johanna Gunnlaugsdottir, Marc J Gunter, Prakash C Gupta, Rajeev Gupta, Oye Gureje, Beata Gurzkowska, Laura Gutierrez, Felix Gutzwiller, Seongjun Ha, Farzad Hadaegh, Rosa Haghshenas, Hamid Hakimi, Jytte Halkjær, Ian R Hambleton, Behrooz Hamzeh, Dominique Hange, Abu AM Hanif, Sari Hantunen, Jie Hao, Carla Menêses Hardman, Rachakulla Hari Kumar, Seyed Mohammad Hashemi-Shahri, Jun Hata, Teresa Haugsgjerd, Alison J Hayes, Yuna He, Margit Heier, Marleen Elisabeth Hendriks, Rafael dos Santos Henrique, Ana Henriques, Leticia Hernandez Cadena, Herqutanto, Sauli Herrala, Ramin Heshmat, Allan G Hill, Sai Yin Ho, Suzanne C Ho, Michael Hobbs, Michelle Holdsworth, Reza Homayounfar, Gonul Horasan Dinc, Andrea RVR Horimoto, Claudia M Hormiga, Bernardo L Horta, Leila Houti, Christina Howitt, Thein Thein Htay, Aung Soe Htet, Maung Maung Than Htike, Yonghua Hu, José María Huerta, Ilpo Tapani Huhtaniemi, Laetitia Huiart, Martijn Huisman, Abdullatif S Husseini, Inge Huybrechts, Nahla Hwalla, Licia Iacoviello, Anna G Iannone, Mohsen M Ibrahim, Norazizah Ibrahim Wong, M Arfan Ikram, Violeta Iotova, Vilma E Irazola, Takafumi Ishida, Godsent C Isiguzo, Muhammad Islam, Sheikh Mohammed Shariful Islam, Masanori Iwasaki, Rod T Jackson, Jeremy M Jacobs, Hashem Y Jaddou, Tazeen Jafar, Kenneth James, Konrad Jamrozik, Imre Janszky, Edward Janus, Marjo-Riitta Jarvelin, Grazyna Jasienska, Ana Jelaković, Bojan Jelaković, Garry Jennings, Anjani Kumar Jha, Chao Qiang Jiang, Ramon O Jimenez, Karl-Heinz Jöckel, Michel Joffres, Mattias Johansson, Jari J Jokelainen, Jost B Jonas, Torben Jørgensen, Pradeep Joshi, Farahnaz Joukar, Jacek Jóżwiak, Anne Juolevi, Gregor Jurak, Vesna Jureša, Rudolf Kaaks, Anthony Kafatos, Eero O Kajantie, Zhanna Kalmatayeva, Natasa Kalpourtzi, Ofra Kalter-Leibovici, Freja B Kampmann, Srinivasan Kannan, Eva Karaglani, Line L Kårhus, Khem B Karki, Marzieh Katibeh, Joanne Katz, Jussi Kauhanen, Prabhdeep Kaur, Maryam Kavousi, Gyulli M Kazakbaeva, Ulrich Keil, Lital Keinan Boker, Sirkka Keinänen-Kiukaanniemi, Roya Kelishadi, Han CG Kemper, Maryam Keramati, Alina Kerimkulova, Mathilde Kersting, Timothy Key, Yousef Saleh Khader, Davood Khalili, Kay-Tee Khaw, Bahareh Kheiri, Motahareh Kheradmand, Alireza Khosravi, Ursula Kiechl-Kohlendorfer, Stefan Kiechl, Japhet Killewo, Dong Wook Kim, Jeongseon Kim, Heidi Klakk, Magdalena Klimek, Jurate Klumbiene, Michael Knoflach, Elin Kolle, Patrick Kolsteren, Jukka P Kontto, Raija Korpelainen, Paul Korrovits, Jelena Kos, Seppo Koskinen, Katsuyasu Kouda, Sudhir Kowlessur, Slawomir Koziel, Jana Kratenova, Vilma Kriaucioniene, Peter Lund Kristensen, Steiner Krokstad, Daan Kromhout, Herculina S Kruger, Ruzena Kubinova, Renata Kuciene, Urho M Kujala, Zbigniew Kulaga, R Krishna Kumar, Pawel Kurjata, Yadlapalli S Kusuma, Vladimir Kutsenko, Kari Kuulasmaa, Catherine Kyobutungi, Tiina Laatikainen, Carl Lachat, Youcef Laid, Tai Hing Lam, Orlando Landrove, Vera Lanska, Georg Lappas, Bagher Larijani, Tint Swe Latt, Gwenaëlle Le Coroller, Khanh Le Nguyen Bao, Tuyen D Le, Jeannette Lee, Jeonghee Lee, Nils Lehmann, Terho Lehtimäki, Daniel Lemogoum, Naomi S Levitt, Yanping Li, Christa L Lilly, Wei-Yen Lim, M Fernanda Lima-Costa, Xu Lin, Yi-Ting Lin, Lars Lind, Vijaya Lingam, Allan Linneberg, Lauren Lissner, Mieczyslaw Litwin, Wei-Cheng Lo, Helle-Mai Loit, Esther Lopez-Garcia, Tania Lopez, Paulo A Lotufo, José Eugenio Lozano, Iva Lukačević Lovrenčić, Janice L Lukrafka, Dalia Luksiene, Annamari Lundqvist, Robert Lundqvist, Nuno Lunet, Michala Lustigová, Edyta Luszczki, Guansheng Ma, Jun Ma, George LL Machado-Coelho, Aristides M Machado-Rodrigues, Enguerran Macia, Luisa M Macieira, Ahmed A Madar, Stefania Maggi, Dianna J Magliano, Emmanuella Magriplis, Gowri Mahasampath, Bernard Maire, Marjeta Majer, Marcia Makdisse, Fatemeh Malekzadeh, Reza Malekzadeh, Rahul Malhotra, Kodavanti Mallikharjuna Rao, Sofia K Malyutina, Lynell V Maniego, Yannis Manios, Jim I Mann, Fariborz Mansour-Ghanaei, Enzo Manzato, Anie Marcil, Staffan B Mårild, Mihalea Marinović Glavić, Pedro Marques-Vidal, Larissa Pruner Marques, Jaume Marrugat, Reynaldo Martorell, Luis P Mascarenhas, Marija Matasin, Ellisiv B Mathiesen, Prashant Mathur, Alicia Matijasevich, Piotr Matlosz, Tandi E Matsha, Christina Mavrogianni, Jean Claude N Mbanya, Anselmo J Mc Donald Posso, Shelly R McFarlane, Stephen T McGarvey, Stela McLachlan, Rachael M McLean, Scott B McLean, Breige A McNulty, Sounnia Mediene Benchekor, Jurate Medzioniene, Parinaz Mehdipour, Kirsten Mehlig, Amir Houshang Mehrparvar, Aline Meirhaeghe, Christa Meisinger, Carlos Mendoza Montano, Ana Maria B Menezes, Geetha R Menon, Alibek Mereke, Indrapal I Meshram, Andres Metspalu, Haakon E Meyer, Jie Mi, Nathalie Michels, Kairit Mikkel, Karolina Milkowska, Jody C Miller, Cláudia S Minderico, GK Mini, Mohammad Reza Mirjalili, Erkin Mirrakhimov, Marjeta Mišigoj-Duraković, Pietro A Modesti, Sahar Saeedi Moghaddam, Bahram Mohajer, Mostafa K Mohamed, Shukri F Mohamed, Kazem Mohammad, Mohammad Reza Mohammadi, Zahra Mohammadi, Noushin Mohammadifard, Reza Mohammadpourhodki, Viswanathan Mohan, Salim Mohanna, Muhammad Fadhli Mohd Yusoff, Iraj Mohebbi, Farnam Mohebi, Marie Moitry, Line T Møllehave, Dénes Molnár, Amirabbas Momenan, Charles K Mondo, Eric Monterrubio-Flores, Kotsedi Daniel K Monyeki, Jin Soo Moon, Mahmood Moosazadeh, Leila B Moreira, Alain Morejon, Luis A Moreno, Karen Morgan, George Moschonis, Malgorzata Mossakowska, Aya Mostafa, Seyed-Ali Mostafavi, Jorge Mota, Mohammad Esmaeel Motlagh, Jorge Motta, Marcos André Moura-dos-Santos, Malay K Mridha, Kelias P Msyamboza, Thet Thet Mu, Alfa J Muhihi, Maria L Muiesan, Martina Müller-Nurasyid, Neil Murphy, Jaakko Mursu, Kamarul Imran Musa, Sanja Musić Milanović, Vera Musil, Norlaila Mustafa, Iraj Nabipour, Shohreh Naderimagham, Gabriele Nagel, Balkish M Naidu, Farid Najafi, Harunobu Nakamura, Jana Námešná, Ei Ei K Nang, Vinay B Nangia, Sameer Narake, Ndeye Coumba Ndiaye, William A Neal, Azim Nejatizadeh, Ilona Nenko, Martin Neovius, Chung T Nguyen, Nguyen D Nguyen, Quang V Nguyen, Quang Ngoc Nguyen, Ramfis E Nieto-Martínez, Teemu J Niiranen, Yury P Nikitin, Toshiharu Ninomiya, Sania Nishtar, Marina A Njelekela, Marianna Noale, Oscar A Noboa, Ahmad Ali Noorbala, Teresa Norat, Maria Nordendahl, Børge G Nordestgaard, Davide Noto, Natalia Nowak-Szczepanska, Mohannad Al Nsour, Baltazar Nunes, Terence W O'Neill, Dermot O'Reilly, Caleb Ochimana, Eiji Oda, Augustine N Odili, Kyungwon Oh, Kumiko Ohara, Ryutaro Ohtsuka, Valérie Olié, Maria Teresa A Olinto, Isabel O Oliveira, Mohd Azahadi Omar, Altan Onat, Sok King Ong, Lariane M Ono, Pedro Ordunez, Rui Ornelas, Pedro J Ortiz, Clive Osmond, Sergej M Ostojic, Afshin Ostovar, Johanna A Otero, Kim Overvad, Ellis Owusu-Dabo, Fred Michel Paccaud, Cristina Padez, Elena Pahomova, Karina Mary de Paiva, Andrzej Pająk, Domenico Palli, Luigi Palmieri, Wen-Harn Pan, Songhomitra Panda-Jonas, Francesco Panza, Mariela Paoli, Dimitrios Papandreou, Soon-Woo Park, Suyeon Park, Winsome R Parnell, Mahboubeh Parsaeian, Patrick Pasquet, Nikhil D Patel, Halyna Pavlyshyn, Ivan Pećin, Mangesh S Pednekar, João M Pedro, Nasheeta Peer, Sergio Viana Peixoto, Markku Peltonen, Alexandre C Pereira, Karen GDA Peres, Marco A Peres, Annette Peters, Janina Petkeviciene, Niloofar Peykari, Son Thai Pham, Rafael N Pichardo, Iris Pigeot, Hynek Pikhart, Aida Pilav, Lorenza Pilotto, Freda Pitakaka, Aleksandra Piwonska, Andreia n Pizarro, Pedro Plans-Rubió, Ozren Polašek, Miquel Porta, Anil Poudyal, Farhad Pourfarzi, Akram Pourshams, Hossein Poustchi, Rajendra Pradeepa, Alison J Price, Jacqueline F Price, Rui Providencia, Soile E Puhakka, Maria Puiu, Margus Punab, Radwan F Qasrawi, Mostafa Qorbani, Daniel Queiroz, Tran Quoc Bao, Ivana Radić, Ricardas Radisauskas, Salar Rahimikazerooni, Mahfuzar Rahman, Olli Raitakari, Manu Raj, Ellina M Rakhimova, Sudha Ramachandra Rao, Ambady Ramachandran, Elisabete Ramos, Lekhraj Rampal, Sanjay Rampal, Daniel A Rangel Reina, Vayia Rarra, Cassiano Ricardo Rech, Josep Redon, Paul Ferdinand M Reganit, Valéria Regecová, Luis Revilla, Abbas Rezaianzadeh, Robespierre Ribeiro, Elio Riboli, Adrian Richter, Fernando Rigo, Tobias F Rinke de Wit, Raphael M Ritti-Dias, Cynthia Robitaille, Fernando Rodríguez-Artalejo, María del Cristo Rodriguez-Perez, Laura A Rodríguez-Villamizar, Ulla Roggenbuck, Rosalba Rojas-Martinez, Dora Romaguera, Elisabetta L Romeo, Annika Rosengren, Joel GR Roy, Adolfo Rubinstein, Jean-Bernard Ruidavets, Blanca Sandra Ruiz-Betancourt, Maria Ruiz-Castell, Iuliia A Rusakova, Paola Russo, Marcin Rutkowski, Charumathi Sabanayagam, Hamideh Sabbaghi, Harshpal S Sachdev, Alireza Sadjadi, Ali Reza Safarpour, Sare Safi, Saeid Safiri, Olfa Saidi, Sibel Sakarya, Nader Saki, Benoit Salanave, Eduardo Salazar Martinez, Diego Salmerón, Veikko Salomaa, Jukka T Salonen, Massimo Salvetti, Jose Sánchez-Abanto, Susana Sans, Diana A Santos, Ina S Santos, Lèlita C Santos, Maria Paula Santos, Rute Santos, Jouko L Saramies, Luis B Sardinha, Giselle Sarganas, Nizal Sarrafzadegan, Thirunavukkarasu Sathish, Kai-Uwe Saum, Savvas Savva, Norie Sawada, Mariana Sbaraini, Marcia Scazufca, Beatriz D Schaan, Herman Schargrodsky, Sabine Schipf, Carsten O Schmidt, Peter Schnohr, Ben Schöttker, Sara Schramm, Constance Schultsz, Aletta E Schutte, Sylvain Sebert, Aye Aye Sein, Abhijit Sen, Idowu O Senbanjo, Sadaf G Sepanlou, Jennifer Servais, Svetlana A Shalnova, Teresa Shamah-Levy, Morteza Shamshirgaran, Coimbatore Subramaniam Shanthirani, Maryam Sharafkhah, Sanjib K Sharma, Jonathan E Shaw, Amaneh Shayanrad, Ali Akbar Shayesteh, Zumin Shi, Kenji Shibuya, Hana Shimizu-Furusawa, Dong Wook Shin, Majid Shirani, Rahman Shiri, Namuna Shrestha, Khairil Si-Ramlee, Alfonso Siani, Rosalynn Siantar, Abla M Sibai, Caroline Ramos de Moura Silva, Diego Augusto Santos Silva, Mary Simon, Judith Simons, Leon A Simons, Michael Sjöström, Jolanta Slowikowska-Hilczer, Przemyslaw Slusarczyk, Liam Smeeth, Hung-Kwan So, Fernanda Cunha Soares, Eugène Sobngwi, Stefan Söderberg, Agustinus Soemantri, Reecha Sofat, Vincenzo Solfrizzi, Mohammad Hossein Somi, Emily Sonestedt, Yi Song, Thorkild IA Sørensen, Elin P Sørgjerd, Maroje Sorić, Charles Sossa Jérome, Aïcha Soumaré, Bente Sparboe-Nilsen, Karen Sparrenberger, Jan A Staessen, Gregor Starc, Bill Stavreski, Jostein Steene-Johannessen, Peter Stehle, Aryeh D Stein, George S Stergiou, Jochanan Stessman, Jutta Stieber, Doris Stöckl, Tanja Stocks, Jakub Stokwiszewski, Karien Stronks, Maria Wany Strufaldi, Machi Suka, Chien-An Sun, Yn-Tz Sung, Paibul Suriyawongpaisal, Rody G Sy, Holly E Syddall, René Charles Sylva, Moyses Szklo, E Shyong Tai, Mari-Liis Tammesoo, Abdonas Tamosiunas, Eng Joo Tan, Xun Tang, Frank Tanser, Yong Tao, Mohammed Rasoul Tarawneh, Carolina B Tarqui-Mamani, Anne Taylor, Julie Taylor, William R Tebar, Grethe S Tell, Tania Tello, Yih Chung Tham, KR Thankappan, Holger Theobald, Xenophon Theodoridis, Lutgarde Thijs, Mikael Thinggaard, Nihal Thomas, Barbara Thorand, Betina H Thuesen, Erik J Timmermans, Dwi H Tjandrarini, Anne Tjonneland, Ulla Toft, Hanna K Tolonen, Janne S Tolstrup, Murat Topbas, Roman Topór-Madry, María José Tormo, Michael J Tornaritis, Maties Torrent, Laura Torres-Collado, Giota Touloumi, Pierre Traissac, Areti Triantafyllou, Dimitrios Trichopoulos, Antonia Trichopoulou, Oanh TH Trinh, Atul Trivedi, Lechaba Tshepo, Shoichiro Tsugane, Azaliia M Tuliakova, Marshall K Tulloch-Reid, Fikru Tullu, Tomi-Pekka Tuomainen, Jaakko Tuomilehto, Maria L Turley, Gilad Twig, Per Tynelius, Christophe Tzourio, Peter Ueda, Eunice Ugel, Hanno Ulmer, Hannu MT Uusitalo, Gonzalo Valdivia, Damaskini Valvi, Rob M van Dam, Bert-Jan van den Born, Johan Van der Heyden, Yvonne T van der Schouw, Koen Van Herck, Hoang Van Minh, Natasja M Van Schoor, Irene GM van Valkengoed, Elisabeth M van Zutphen, Dirk Vanderschueren, Diego Vanuzzo, Anette Varbo, Senthil K Vasan, Tomas Vega, Toomas Veidebaum, Gustavo Velasquez-Melendez, Giovanni Veronesi, WM Monique Verschuren, Roosmarijn Verstraeten, Cesar G Victora, Lucie Viet, Salvador Villalpando, Paolo Vineis, Jesus Vioque, Jyrki K Virtanen, Sophie Visvikis-Siest, Bharathi Viswanathan, Tiina Vlasoff, Peter Vollenweider, Ari Voutilainen, Alisha N Wade, Janette Walton, Elvis OA Wambiya, Wan Mohamad Wan Bebakar, Wan Nazaimoon Wan Mohamud, Rildo de Souza Wanderley Júnior, Ming-Dong Wang, Ningli Wang, Qian Wang, Xiangjun Wang, Ya Xing Wang, Ying-Wei Wang, S Goya Wannamethee, Nicholas Wareham, Wenbin Wei, Aneta Weres, Bo Werner, Peter H Whincup, Kurt Widhalm, Andrzej Wiecek, Rainford J Wilks, Johann Willeit, Peter Willeit, Emmanuel A Williams, Tom Wilsgaard, Bogdan Wojtyniak, Roy A Wong-McClure, Andrew Wong, Tien Yin Wong, Jean Woo, Frederick C Wu, Shouling Wu, Justyna Wyszynska, Haiquan Xu, Liang Xu, Nor Azwany Yaacob, Weili Yan, Ling Yang, Xiaoguang Yang, Yang Yang, Tabara Yasuharu, Xingwang Ye, Panayiotis K Yiallouros, Moein Yoosefi, Akihiro Yoshihara, San-Lin You, Novie O Younger-Coleman, Ahmad Faudzi Yusoff, Ahmad A Zainuddin, Seyed Rasoul Zakavi, Farhad Zamani, Sabina Zambon, Antonis Zampelas, Maria Elisa Zapata, Ko Ko Zaw, Kristyna Zejglicova, Tajana Zeljkovic Vrkic, Yi Zeng, Luxia Zhang, Zhen-Yu Zhang, Dong Zhao, Ming-Hui Zhao, Shiqi Zhen, Yingfeng Zheng, Bekbolat Zholdin, Dan Zhu, Marie Zins, Emanuel Zitt, Yanina Zocalo, Nada Zoghlami, Julio Zuñiga Cisneros, Majid Ezzati

## Abstract

**Background:**

Hypertension can be detected at the primary health-care level and low-cost treatments can effectively control hypertension. We aimed to measure the prevalence of hypertension and progress in its detection, treatment, and control from 1990 to 2019 for 200 countries and territories.

**Methods:**

We used data from 1990 to 2019 on people aged 30–79 years from population-representative studies with measurement of blood pressure and data on blood pressure treatment. We defined hypertension as having systolic blood pressure 140 mm Hg or greater, diastolic blood pressure 90 mm Hg or greater, or taking medication for hypertension. We applied a Bayesian hierarchical model to estimate the prevalence of hypertension and the proportion of people with hypertension who had a previous diagnosis (detection), who were taking medication for hypertension (treatment), and whose hypertension was controlled to below 140/90 mm Hg (control). The model allowed for trends over time to be non-linear and to vary by age.

**Findings:**

The number of people aged 30–79 years with hypertension doubled from 1990 to 2019, from 331 (95% credible interval 306–359) million women and 317 (292–344) million men in 1990 to 626 (584–668) million women and 652 (604–698) million men in 2019, despite stable global age-standardised prevalence. In 2019, age-standardised hypertension prevalence was lowest in Canada and Peru for both men and women; in Taiwan, South Korea, Japan, and some countries in western Europe including Switzerland, Spain, and the UK for women; and in several low-income and middle-income countries such as Eritrea, Bangladesh, Ethiopia, and Solomon Islands for men. Hypertension prevalence surpassed 50% for women in two countries and men in nine countries, in central and eastern Europe, central Asia, Oceania, and Latin America. Globally, 59% (55–62) of women and 49% (46–52) of men with hypertension reported a previous diagnosis of hypertension in 2019, and 47% (43–51) of women and 38% (35–41) of men were treated. Control rates among people with hypertension in 2019 were 23% (20–27) for women and 18% (16–21) for men. In 2019, treatment and control rates were highest in South Korea, Canada, and Iceland (treatment >70%; control >50%), followed by the USA, Costa Rica, Germany, Portugal, and Taiwan. Treatment rates were less than 25% for women and less than 20% for men in Nepal, Indonesia, and some countries in sub-Saharan Africa and Oceania. Control rates were below 10% for women and men in these countries and for men in some countries in north Africa, central and south Asia, and eastern Europe. Treatment and control rates have improved in most countries since 1990, but we found little change in most countries in sub-Saharan Africa and Oceania. Improvements were largest in high-income countries, central Europe, and some upper-middle-income and recently high-income countries including Costa Rica, Taiwan, Kazakhstan, South Africa, Brazil, Chile, Turkey, and Iran.

**Interpretation:**

Improvements in the detection, treatment, and control of hypertension have varied substantially across countries, with some middle-income countries now outperforming most high-income nations. The dual approach of reducing hypertension prevalence through primary prevention and enhancing its treatment and control is achievable not only in high-income countries but also in low-income and middle-income settings.

**Funding:**

WHO.

## Introduction

Hypertension, along with pre-hypertension and other hazardously high blood pressure, is responsible for 8·5 million deaths from stroke, ischaemic heart disease, other vascular diseases, and renal disease worldwide.^1^, ^2^ Hypertension can be detected in the community and primary care facilities, and several effective drugs are available at fairly low cost for treating patients with hypertension and reducing the risk of its sequelae.^1^, ^3^, ^4^, ^5^ Improving the effective coverage of treatment for patients with hypertension is an objective of many global, regional, and national initiatives, and programmes.

**Research in context**

**Evidence before this study**

We searched MEDLINE (via PubMed) for articles published from inception to Jan 15, 2021, using the search terms ((hypertension[Title] AND (((medication OR treatment) AND control) OR aware*) AND “blood pressure”) OR (cardiovascular[Title] AND risk factor*[Title] AND “blood pressure” AND (((medication OR treatment) AND control) OR aware*))) AND (trend* OR global OR worldwide) NOT patient*[Title]. No language restrictions were applied. We found a few multi-country studies that reported hypertension prevalence, treatment, and control. These studies used up to 135 data sources that had sampled from national or sub-national populations or data from small communities. Few multi-country studies reported trends over time. The largest of these analyses covered snapshots in 2000 and 2010 and grouped countries into high income versus low income and middle income. We also found several studies that analysed trends in individual countries. To our knowledge, there is no study on long-term trends in, nor the contemporary levels of, hypertension prevalence, detection, treatment, and control that covers the entire world.

**Added value of this study**

To our knowledge, this study is the first comprehensive global analysis of trends in hypertension prevalence, detection, treatment, and control that covers all countries worldwide. The data used in the study were from 184 countries, together covering 99% of the global population, and were subject to rigorous inclusion and exclusion criteria. Data were analysed using a standardised protocol and were pooled using a statistical model designed to incorporate how hypertension and its care and control vary in relation to age, geography, and time.

**Implications of all the available evidence**

Hypertension care—including detection, treatment, and control—varies substantially worldwide and even within the same region of the world. Sub-Saharan Africa, Oceania, and south Asia have the lowest rates of detection, treatment, and control and many countries in these regions have seen little improvement in these outcomes over the past 30 years. The large improvements observed in some upper-middle-income and recently high-income countries show that the expansion of universal health coverage and primary care can be leveraged to enhance hypertension care and reduce the health burden of this condition.

Comparable data on hypertension detection, treatment, and control are needed to learn from good practice to guide health system programmes. No comparable global data exist to assess which countries have high versus low rates of detection, treatment, and control, and how these measures have changed over time. We present consistent national, regional, and global estimates of trends in hypertension prevalence, detection, treatment and control from 1990 to 2019 for 200 countries and territories (referred to as countries hereafter).

## Methods

### Data sources

We used data from 1990 to 2019, collated by the NCD Risk Factor Collaboration (NCD-RisC), as detailed previously^6^ and summarised in the appendix (pp 2–3). The inclusion criteria were that (1) data were collected using a probabilistic sampling method with a defined sampling frame; (2) data were from population samples at the national, sub-national (covering one or more sub-national regions), or community (one or a small number of communities) level; (3) systolic blood pressure and diastolic blood pressure were measured; and (4) data on hypertension treatment were available.

Studies were excluded if they (1) included or excluded participants on the basis of health status; (2) were done only among minority ethnic groups or specific educational, occupational, or other socioeconomic groups; (3) recruited participants through health facilities, except studies whose sampling frame was health insurance schemes in countries where at least 80% of the population were insured, and studies based on primary care systems in high-income and central European countries with universal insurance; or (4) had not measured blood pressure. A list of data sources and their characteristics is provided in the appendix (pp 7–30).

We established whether a participant had been diagnosed with hypertension using questions worded as variations of “Have you ever been told by a doctor or other health professional that you had hypertension, also called high blood pressure?” We assessed whether a person was taking medication for hypertension using questions worded as variations of “Are you currently taking any medicines, tablets, or pills for high blood pressure?” or “In the past 2 weeks, have you taken any drugs (medication) for raised blood pressure prescribed by a doctor or other health worker?” In studies that gathered information on prescribed medicines, we used survey information to establish that the purpose of taking a blood pressure-lowering drug was specifically to treat hypertension.

### Outcomes

Our primary outcomes were prevalence of hypertension, the proportion of people with hypertension who reported a previous hypertension diagnosis (detection), who were taking medication for hypertension (treatment), and whose blood pressure was controlled (control).^7^ Hypertension was defined as having systolic blood pressure 140 mm Hg or greater, diastolic blood pressure 90 mm Hg or greater, or taking medication for hypertension. Control was defined as taking medication for hypertension and having systolic blood pressure less than 140 mm Hg and diastolic blood pressure less than 90 mm Hg. We also report the proportion of people with hypertension who were undiagnosed or untreated with systolic blood pressure 160 mm Hg or greater or diastolic blood pressure 100 mm Hg or greater. We restricted our analysis to men and women aged 30–79 years because hypertension prevalence is relatively low before age 30 years and because guidelines differ in thresholds and treatment targets in older ages.^8^

### Statistical analysis

We calculated the prevalence, detection, treatment, and control of hypertension by sex and age group for each study. The denominators for detection, treatment, and control were the number of people with hypertension. When applicable, we used survey sample weights and accounted for complex survey design.

We applied a Bayesian hierarchical model to these sex-specific and age-specific data to estimate the primary outcomes by country, year, and age. All analyses were done separately by sex and for each primary outcome. The model is described in detail in a statistical paper^9^ and related substantive papers^6^, ^10^ and summarised in the appendix (pp 4–6). Countries were grouped into 21 regions, which were further grouped into nine super-regions (appendix pp 31–32). In the hierarchical model, estimates for a country-year were informed by its own data if available, by data from other years in the same country, and from other countries, especially those from the same region and super-region. The extent to which estimates for each country-year were influenced by data from other years and countries depended on whether the country had data, sample size, whether data were national, and the within-country and within-region variability of the available data.

The model allowed for non-linear time trends and non-linear age patterns. For this analysis, we adapted the model to allow time trends to vary by age (appendix pp 4–6) because how hypertension and its detection, treatment, and control have changed over time depends on age.^11^, ^12^ The model also accounted for the possibility that hypertension prevalence, detection, treatment, and control in sub-national and community studies might systematically differ from those in nationally representative studies, or might have larger variation than in national studies, so that national data had a larger influence on the estimates than sub-national or community data did with similar sample sizes. Finally, the model accounted and adjusted for how much studies that were done in only rural or urban areas differed from national studies.

We fitted the model using the Markov chain Monte Carlo (MCMC) algorithm implemented in R (version 3.6.0), and obtained 50 000 post-burn-in samples from the posterior distribution of model parameters. We kept every 10th sample, and the resultant 5000 samples were used to obtain the posterior distributions of the primary outcomes. The reported 95% credible intervals (CrIs) are the 2·5th to 97·5th percentiles of the posterior distributions. We calculated age-standardised hypertension prevalence, and the rates of detection, treatment, and control, by weighting age-specific estimates using the WHO standard population.^13^ When calculating age-standardised detection, treatment, and control rates, we also accounted for the age pattern of hypertension prevalence, which appears in the denominator, by using the combination of WHO standard population weights and age-specific hypertension prevalence in each country and year to weight age-specific estimates. Estimates for regions, super-regions, and the world were calculated by weighting the age-specific and sex-specific posterior samples for the constituent countries with the corresponding age-specific and sex-specific national populations; the population data were from World Population Prospects (2019 revision).^14^ The estimates in each country and region and in each year are for the corresponding national and regional population in that year. We used consistent analysis and presentation units over the entire 30-year period. For countries that were formed during these 30 years (eg, South Sudan and Montenegro), estimates apply to an equivalent territory for the years before their formation.

### Role of the funding source

The funder of the study had no role in study design, data collection, data analysis, data interpretation, or writing of the report.

## Results

**Figure 1:**
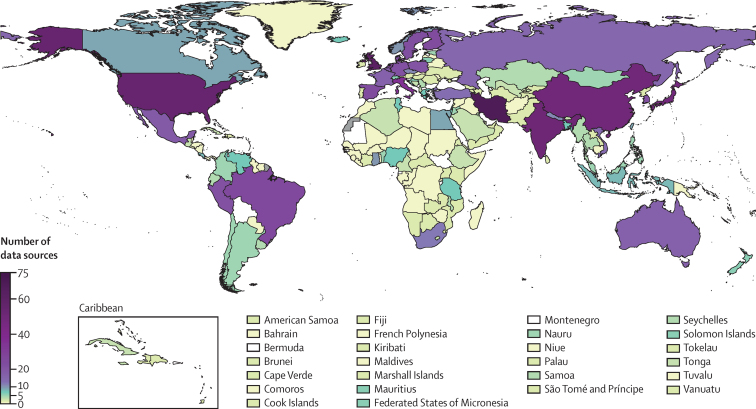
Number of data sources by country

We used 1201 studies carried out from 1990 to 2019 with data on 104 million participants aged 30–79 years. Of these, 986 (82·1%) studies also had information on previous diagnosis. 184 countries, covering 99% of the global population, had at least one data source (figure 1), and 131 countries, covering 94% of the world's population, had two or more data sources. Regionally, data availability ranged from 2·2 data sources per country in sub-Saharan Africa to 26·0 data sources per country in the high-income Asia-Pacific region (figure 1).

**Figure 2:**
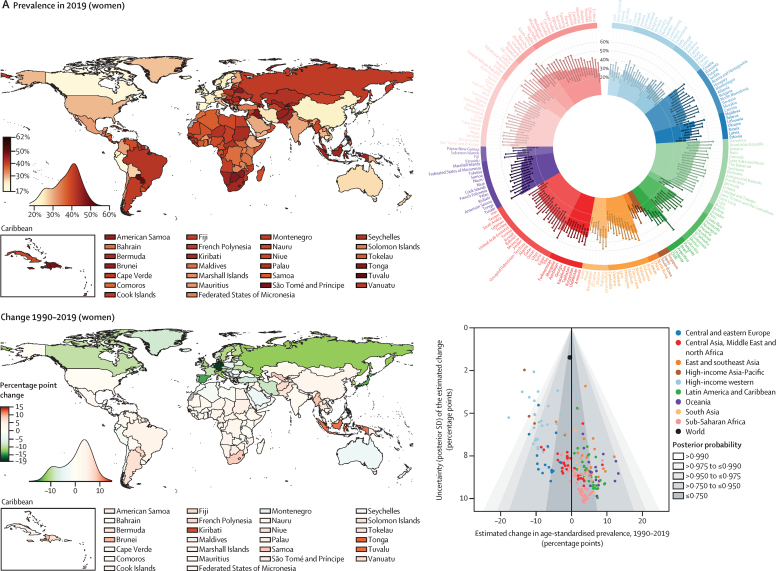

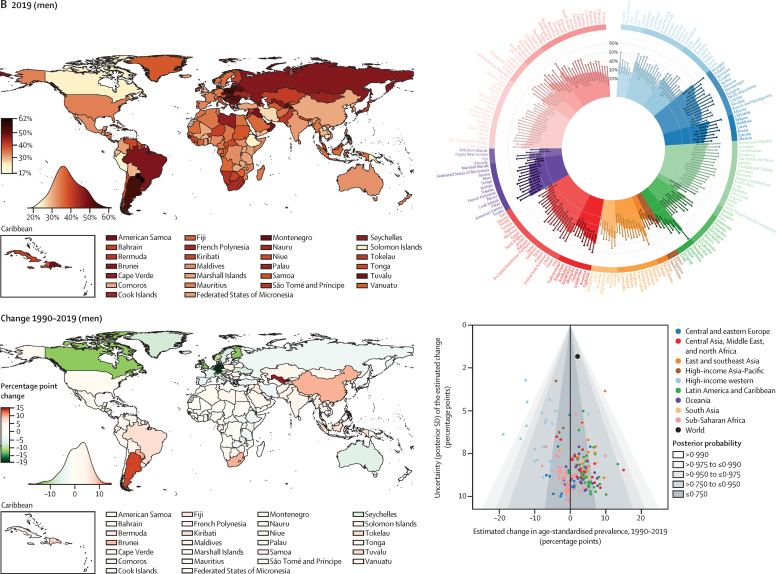
Prevalence of hypertension in 2019 and change from 1990 to 2019 in women and men Prevalence of hypertension in 2019 and change from 1990 to 2019 in women (A) and men (B). The density plot alongside each map shows the distribution of estimates across countries. The top right graph in each panel shows results ordered within regions and super-regions with 95% credible intervals. The bottom right graph in each panel shows the change from 1990 to 2019 in hypertension prevalence in relation to the uncertainty of the change measured by posterior SD. Shaded areas show the posterior probability of an estimated increase or decrease being a true increase or decrease. Each point shows one country. See the appendix (pp 33–46) for numerical results.

In 2019, the global age-standardised prevalence of hypertension in adults aged 30–79 years was 32% (95% CrI 30–34) in women and 34% (32–37) in men, similar to 1990 levels of 32% (30–35) in women and 32% (30–35) in men (figure 2). The stable global prevalence was a net effect of a decrease in high-income countries, and for women also in central and eastern Europe, and an increase in some low-income and middle-income countries. The decline was greater than 12 percentage points in women in several high-income countries (posterior probability [PP] of the observed decline being a true decline >0·98 for all country and sex combinations; figure 2). By contrast, age-standardised prevalence increased, or at best remained unchanged, in most low-income and middle-income countries (figure 2). The increase was 10–15 percentage points among men in three countries and among women in four countries (PP 0·85–0·99).

Nationally, prevalence of hypertension in 2019 was lowest in Canada and Peru for both men and women; in Taiwan, South Korea, Japan, and some countries in western Europe for women; and in some low-income and middle-income countries for men (figure 2). Age-standardised prevalence in all of these countries was less than 24% for women and less than 25% for men in 2019 (figure 2). Hypertension prevalence was highest throughout central and eastern Europe, central Asia, Oceania, southern Africa, and some countries in Latin America and the Caribbean (figure 2). For women in two countries and men in nine countries, age-standardised prevalence surpassed 50% (figure 2).

**Figure 3:**
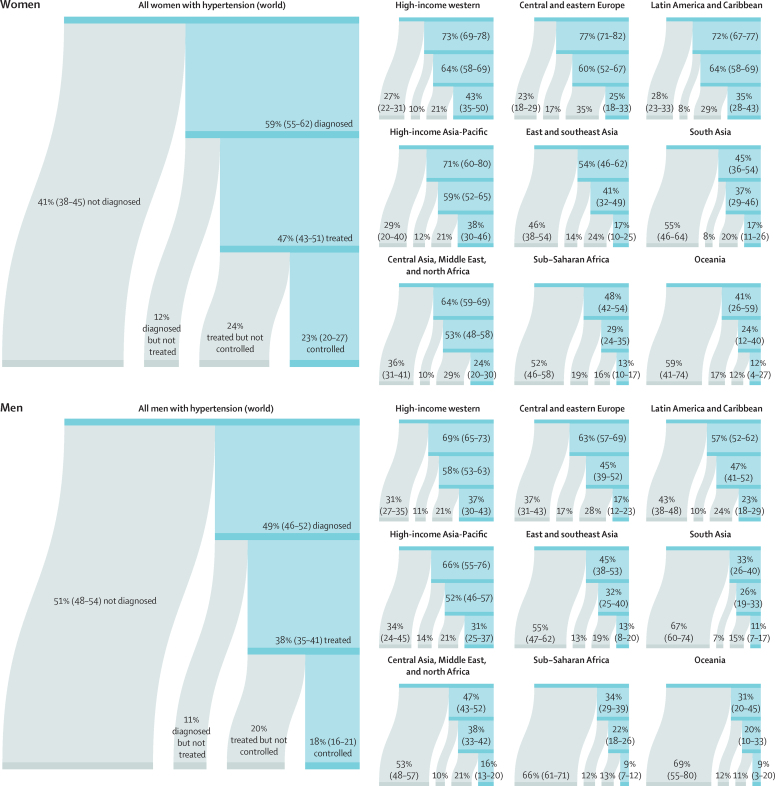
Hypertension treatment cascade in 2019, for women and men globally and by region Data are estimate (95% credible interval). Each stream shows the loss of people with hypertension throughout the treatment cascade and its associated percentage for women and men.

Globally, 41% (95% CrI 38–45) of women and 51% (48–54) of men with hypertension did not report a previous diagnosis (figure 3). The treatment rate was 47% (43–51) in women and 38% (35–41) in men. Less than half of those treated had achieved hypertension control, leading to global control rates of 23% (20–27) for women and 18% (16–21) for men with hypertension (figure 3). 27–34% of women and men in the high-income western and Asia-Pacific regions with hypertension were not aware of their condition, an additional 10–14% were untreated, and 21% did not achieve control (figure 3). The detection gap, together with sequential low treatment coverage and effectiveness, led to control rates ranging from 31% in men in the high-income Asia-Pacific to 43% in women in the high-income western region (figure 3). Control rates were below 13% in sub-Saharan Africa and Oceania, where 50–60% of women and nearly 70% of men with hypertension were not aware of their condition; detection, treatment and control rates in south Asia were only slightly higher (figure 3). In all regions the coverage of treatment increased with age, being highest in those aged 65 years and older (appendix pp 50–51).

**Figure 4:**
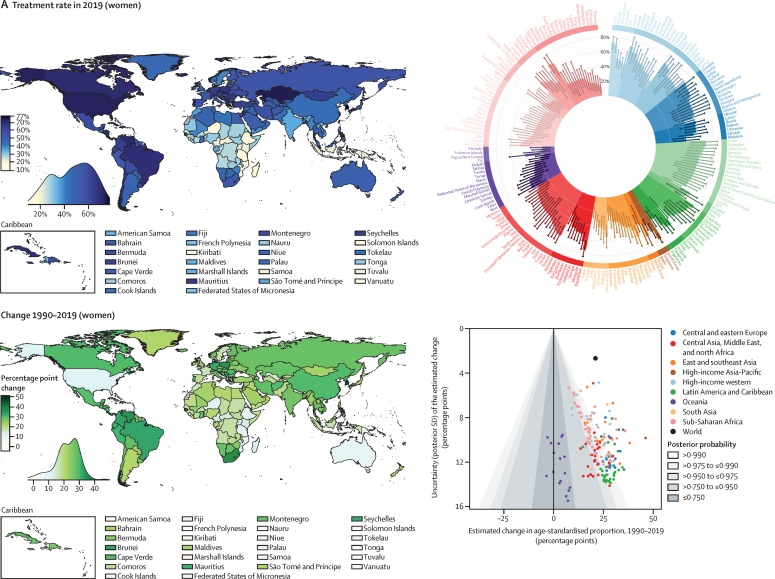

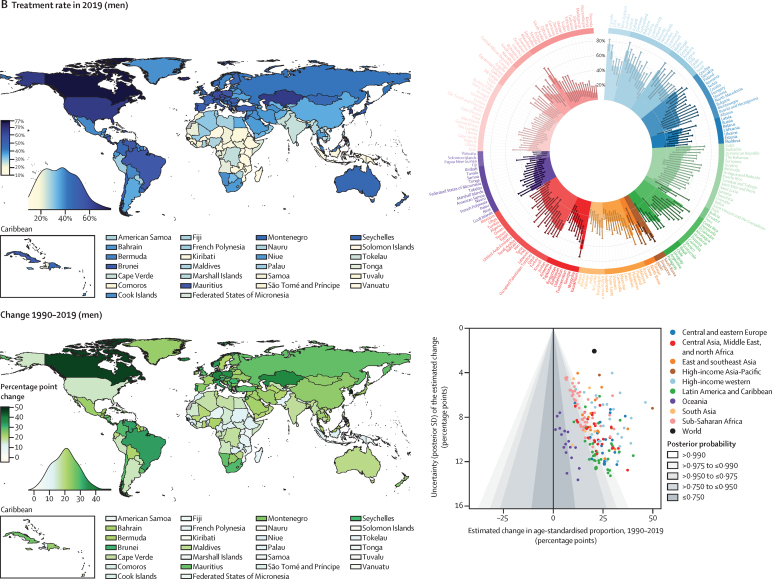

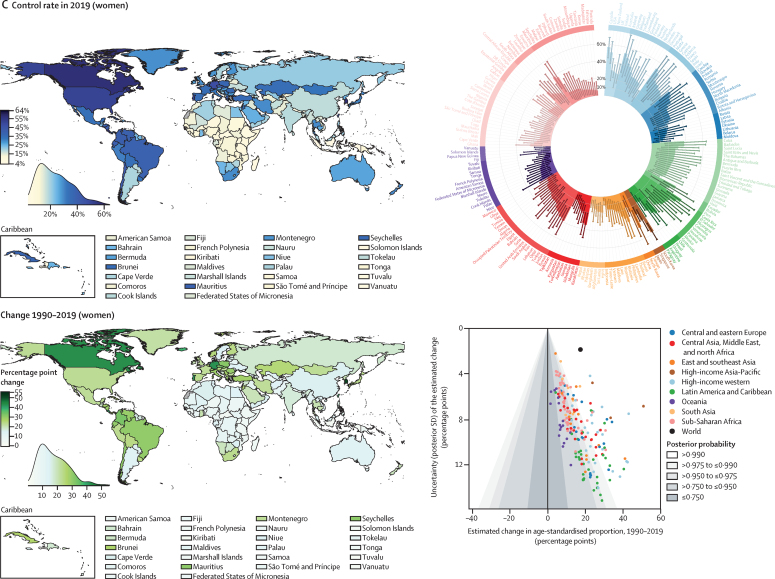

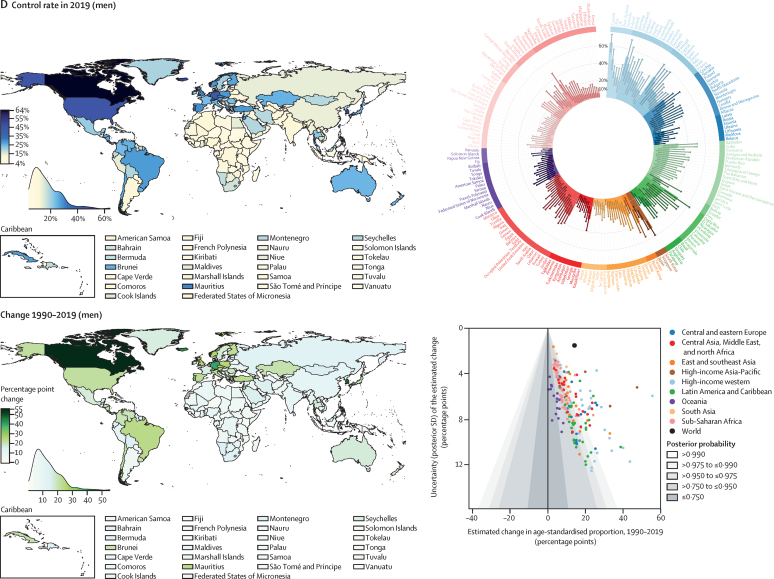
Proportion of women and men with hypertension who used treatment and whose blood pressure was controlled in 2019, and change from 1990 to 2019 See the appendix (pp 52–53) for control rates among those on treatment and for a comparison of treatment and control rates (pp 54–55). The density plot alongside each map shows the distribution of estimates across countries. The top right graph in each panel shows the results ordered within regions and super-regions with their 95% credible intervals. The bottom right graph in each panel shows the change from 1990 to 2019 in hypertension treatment and control rates in relation to the uncertainty of the change measured by posterior SD. Shaded areas show the posterior probability of an estimated increase or decrease being a true increase or decrease. Each point shows one country. See the appendix (pp 33–46) for numerical results.

Nationally, hypertension treatment and control were highest in South Korea, Canada, and Iceland, where more than 70% of women and men with hypertension were treated and over half had their hypertension controlled (figure 4). Treatment and control rates were also high in the USA, Costa Rica, Germany, Portugal, and Taiwan. At the other extreme, treatment rates were less than 25% for women and less than 20% for men in Nepal, Indonesia, and several countries in sub-Saharan Africa and Oceania (figure 4). Control rates were less than 10% for women and men in these countries and for men in some countries in the Middle East and north Africa, central and south Asia, and eastern Europe (figure 4). The proportion of those treated who achieved control varied by more than four times across countries (appendix pp 54–55). In particular, many countries in eastern Europe, central and east Asia, and the Middle East and north Africa had somewhat high treatment rates but low control, contrasting with findings in high-income countries and some countries in Latin America and the Caribbean, where treatment and control tracked more closely (appendix pp 54–55).

Hypertension treatment and control improved in most countries since 1990, but we found little improvement in many countries in sub-Saharan Africa and Oceania (figure 4). Improvements were largest in high-income countries and central Europe, with some countries expanding treatment and control by more than 30 percentage points (figure 4). Some upper-middle-income countries and recently high-income countries in other regions (eg, Costa Rica, Taiwan, Kazakhstan, South Africa, Brazil, Chile, Turkey, and Iran) also substantially enhanced treatment and control (figure 4). Hypertension treatment and control rates were lower in men than in women in most countries (appendix pp 56–57). The male disadvantage in treatment was smaller in high-income countries than elsewhere and, in a few countries, we found the reverse of this pattern (appendix pp 56–57).

**Figure 5:**
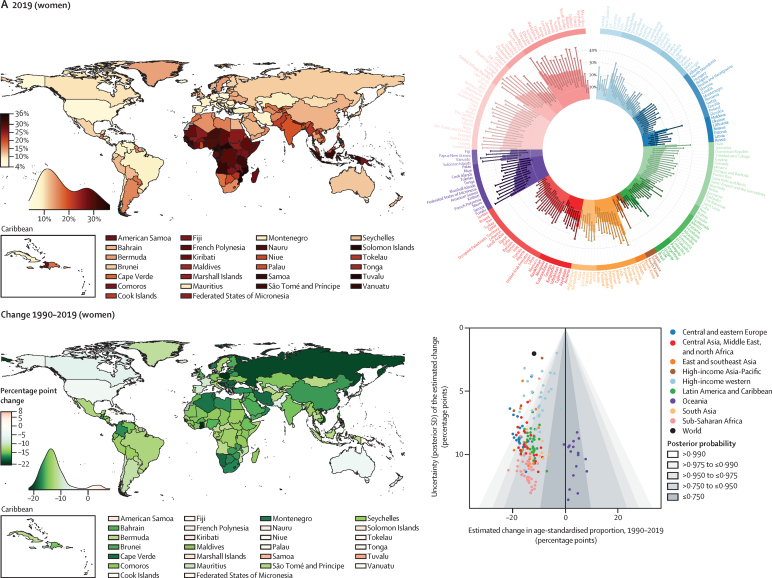

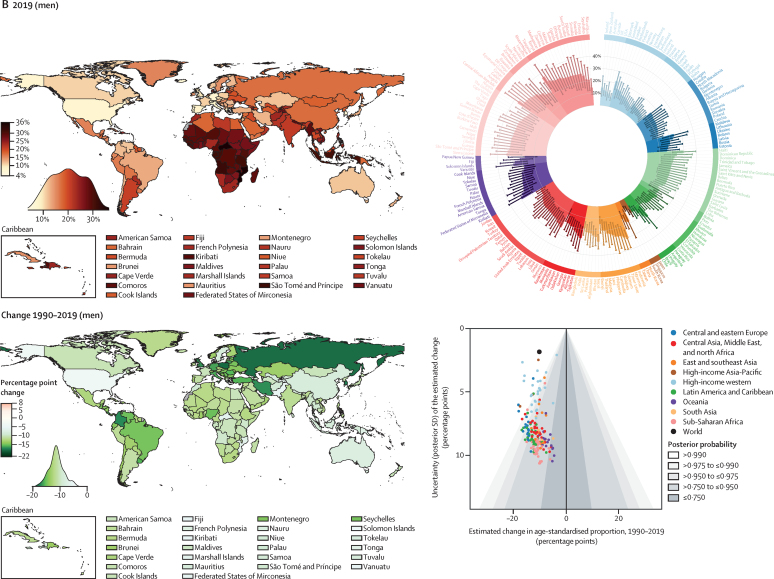
Proportion of women and men with hypertension who had systolic blood pressure 160 mm Hg or greater or diastolic blood pressure 100 mm Hg or greater but were not diagnosed or treated, in 2019, and change from 1990 to 2019 The density plot alongside each map shows the distribution of estimates across countries. The top right graph in each panel shows the results ordered within regions and super-regions with their 95% credible intervals. The bottom right graph in each panel shows the change from 1990 to 2019 in the proportion of people with hypertension who had systolic blood pressure 160 mm Hg or greater or diastolic blood pressure 100 mm Hg or greater but were not diagnosed or treated, in relation to the uncertainty of the change measured by posterior SD. Shaded areas show the posterior probability of an estimated increase or decrease being a true increase or decrease. Each point shows one country.

In 2019, the proportion of people with systolic blood pressure 160 mm Hg or greater or diastolic blood pressure 100 mm Hg or greater but were not diagnosed or treated was below 10% in countries with high treatment coverage, and as low as 4% among women in South Korea (figure 5). Between one in four to one in three women and men with hypertension in many sub-Saharan African and Oceanian countries and in some countries in central, south, and southeast Asia had systolic blood pressure 160 mm Hg or greater or diastolic blood pressure 100 mm Hg or greater but were not diagnosed or treated (figure 5).

**Figure 6:**
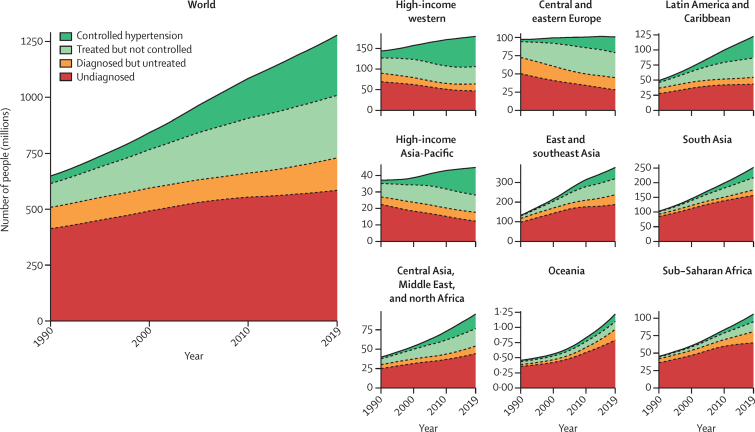
Trends in the number of people with hypertension who reported a diagnosis, who used treatment, and whose blood pressure was effectively controlled, globally and by region, 1990–2019 See the appendix (pp 58–60) for trends in the percentage of people with hypertension who reported a diagnosis, who had treatment, and whose blood pressure was effectively controlled, globally and by region.

Despite stable global prevalence, the absolute number of people aged 30–79 years with hypertension doubled from 331 (95% CrI 306–359) million women and 317 (292–344) million men in 1990 to 626 (584–668) million women and 652 (604–698) million men in 2019 due to population growth and ageing (figure 6). Similarly, despite improvement in detection, treatment, and control rates, more people did not achieve effective control in 2019 than in 1990 because of the large increase in the number of people with hypertension (figure 6).

In high-income western and Asia-Pacific regions and in central and eastern Europe, the opposite effects of declining prevalence and population growth and ageing led to a small net increase in the number of people with hypertension (figure 6). The improvements in treatment and control from 1990 to 2019 shifted many of those with hypertension in these regions from being untreated to being treated and having their hypertension controlled (figure 6). These improvements lowered the absolute number of those who were not treated or whose hypertension was not effectively controlled (figure 6).

In 2019, over 1 billion people with hypertension (82% of all people with hypertension in the world) lived in low-income and middle-income regions (figure 6). This number was much larger than the number in 1990 because prevalence remained unchanged or increased and the population grew and became older. In sub-Saharan Africa, Oceania, and south Asia, most of the increase was in those without a previous diagnosis, whereas in east and southeast Asia and Latin America and the Caribbean the number of people in this category increased slightly until the mid-2000s before flattening. Since then, many more of those with hypertension have been detected, treated, and controlled (figure 6).

## Discussion

Our novel comprehensive analysis of hypertension prevalence and care has shown that since 1990 the number of people with hypertension worldwide has doubled, with most of the increase occurring in low-income and middle-income regions. In high-income countries, prevalence has declined while health systems have achieved treatment rates of up to 80% and control rates of up to 60%. Middle-income countries in Latin America; east and southeast Asia; and central Asia, the Middle East, and north Africa have also enhanced the detection and treatment of hypertension. Some of these countries, such as Costa Rica, now outperform most high-income nations in hypertension treatment and control. Low detection and treatment rates persist in the world's poorest nations, especially in sub-Saharan Africa, Oceania, and south Asia. Together with the increasing number of people who have hypertension, these low detection and treatment rates will shift an increasing share of the burden of vascular and renal conditions to these regions.

To our knowledge, no previous study of trends in hypertension prevalence, detection, treatment, and control covers all countries in the world. Our results are consistent with a multi-country study that reported for 2000 and 2010,^15^ in terms of higher treatment and control in high-income countries than in low-income and middle-income countries, but our national results show that substantial variability exists at any level of economic development, with some upper-middle-income countries having treatment and control rates as good as, or better than, those in some high-income countries. The findings of a study^16^ on 44 low-income and middle-income countries were consistent with ours in terms of hypertension treatment rates being highest in Latin America and lowest in sub-Saharan Africa; although, this study did not have data on trends or from high-income countries. Our finding on variable improvement in rates of hypertension treatment in high-income countries is consistent with a previous multi-country study.^12^

The strengths of our study include its scope of presenting consistent and comparable global estimates of hypertension prevalence, treatment, and control; the scale and quality of data that were harmonised in a rigorous process; and the statistical methods that were designed for analysing trends in the hypertension treatment cascade. We used data from more than 1200 studies in 184 countries, covering 99% of the world's population, which is eight times as many studies as were in the previous largest analysis.^15^ We used only data from studies that had measured blood pressure to avoid bias in self-reported data. We re-analysed data according to a standardised protocol and the characteristics and quality of data were rigorously verified through repeated checks by NCD-RisC members. We used a statistical model that accounted for heterogeneous trends by age in hypertension prevalence, detection, treatment, and control, and we used all available data, while giving more weight to national data than to non-national sources.

Similar to all global analyses, our study has some limitations. Despite our extensive efforts to identify and access data, some countries, especially those in Oceania and sub-Saharan Africa, had less data than in other regions. Most health surveys collect data on previous diagnosis and treatment of hypertension using a questionnaire, which may lead to measurement error. Validation studies show that recall of hypertension diagnosis and medication has good agreement with actual medical history (eg, with Cohen's κ ranging between 0·55 and 0·91).^17^, ^18^, ^19^, ^20^ Mercury sphygmomanometers were more common in earlier studies, whereas studies done after 2000 often used digital oscillometric devices. Similarly, studies differed on whether they used multiple cuff sizes or one cuff size or whether they measured blood pressure more than once. The effect of measurement device and protocol on population prevalence depends on the circumstances of each study. For example, an automated digital device with a standard cuff, although not the traditional gold standard in a clinical setting, avoids observer bias and increases compliance and possibly even response rate, compared with a mercury sphygmomanometer with multiple cuffs.^21^ Nonetheless, measurements from different devices are not fully comparable. Most health surveys are based on one visit to each participant, during which blood pressure is measured multiple times, usually after a resting period when interviews are done. Hypertension prevalence based on data collected in multiple visits might be lower than that based on one visit.^22^ We had insufficient comparable data on treatment details such as the type of drugs because these data are not consistently collected in population-representative surveys. Complementing survey data with data from health facilities or prescriptions could provide such clinically relevant details.

Our country results show that preventing hypertension and enhancing its detection, treatment, and control is feasible not only in high-income countries, but also in low-income and middle-income nations. Although the nutritional, behavioural, and environmental causes of increased blood pressure are well established, little is known on which actions and interventions that can be widely replicated are responsible for the observed reductions in hypertension prevalence.^23^ Similarly, although randomised trials have shown the efficacy of hypertension treatment and studies in some countries or communities have shown that strategies such as simple evidence-based guidelines, the use of non-physician health workers, and patient follow-ups using text messages can improve hypertension care,^1^, ^2^, ^24^, ^25^, ^26^, ^27^, ^28^, ^29^, ^30^ little transferable guidance exists on how to achieve high rates of detection, treatment, and control for entire populations. Implementation research on the role of risk factors and health system determinants of hypertension care and management requires detailed country-level data. Information for seven countries with high rates of treatment is summarised in the appendix (pp 47–49).

Over the period of our analysis, hypertension prevalence decreased while obesity, which is a risk factor for hypertension, increased,^6^ which implies that hypertension's dietary and environmental determinants must have improved. Reducing salt intake to prevent hypertension might be possible through a combination of fiscal, regulatory, and possibly behavioural interventions,^31^, ^32^ although few examples exist of successful national programmes so far. Increased availability and consumption of fruits and vegetables^33^ might partly account for the observed declines in hypertension, which indicates that making these foods affordable (eg, through targeted subsidies for poorer families) and accessible (eg, through more efficient supply and storage) might be effective for hypertension prevention.

Expanding hypertension detection has been helped by both more widespread and regular contact with health services and more frequent measurement of blood pressure.^34^, ^35^ Increased health-care use requires universal health insurance^36^, ^37^, ^38^, ^39^ and expansion of primary care. In some countries, training non-physician health workers in the management of non-communicable diseases (NCDs) might be needed.^24^, ^25^, ^26^, ^27^, ^28^ Guidelines, availability of blood pressure monitors, and regular health checks and screening programmes^40^, ^41^, ^42^, ^43^, ^44^ facilitate more frequent measurement. The expansion of universal health coverage and primary care in places with low rates of diagnosis, especially sub-Saharan Africa and south Asia, provides an opportunity for improving hypertension care,^45^, ^46^ but needs to be accompanied with guidelines,^47^ training, and blood pressure monitors in health facilities. Improvements in treatment have been helped by some of the same factors as those for diagnosis, as well as guidelines that recommend progressively lower thresholds to initiate treatment and wider availability and lower cost of antihypertensive medicines, many of which are no longer under a patent.^48^ Despite this improvement, insufficient access to medicines contributes to the low treatment rates in some low-income countries.^46^, ^49^, ^50^, ^51^

We also found large variation in hypertension control among those who were treated. Understanding the reasons for the large variation in real-world effectiveness of treatment needs data on both the health-system features that enable high-quality care and the type of pharmacological approach used—eg, renin-angiotensin-system inhibitors, calcium-channel blockers, or diuretics;^52^ whether single-pill combination therapy is used;^53^ how much the prescribing physician titrates or intensifies treatment when needed; and patient adherence to treatment. New technologies such as telemonitoring, home blood pressure monitoring, and text message reminders might improve adherence,^29^, ^54^, ^55^, ^56^ but these measures can be effective only if patients have uninterrupted access to effective medicines.

Hypertension prevention and control can make a substantial contribution to achieving the Sustainable Development Goals target 3.4 on NCDs.^57^, ^58^ Some countries, such as Canada, Costa Rica, South Korea, and Taiwan, have achieved low hypertension prevalence or high control through both improved prevention and improving every stage of the treatment cascade.^30^, ^59^ Universal health insurance has been instrumental in achieving high effective coverage but should be complemented with primary care strengthening, evidence-based hypertension guidelines that are up to date and are adapted to the country contexts,^8^, ^47^ health workforce training, and a robust system of drug procurement and distribution.^30^ Programmes should also be regularly assessed, both at the population level, as our work has done, and in health facilities to ensure accountability and stimulate improvement.^60^

## Data sharing

Computer code and age-standardised and crude results of this study can be downloaded from https://www.ncdrisc.org and age-specific results can be requested via the same website. The input data are available at https://www.ncdrisc.org when permitted by data governance and sharing arrangements; contact information is provided for other data sources.

## Declaration of interests

RC reports grants from the Ministry of Health of the Czech Republic; and personal fees from Herbacos Recordati, Amegen, and Krka, outside the submitted work. GD reports consulting fees from Vital Strategies; and an honorarium from the American College of Cardiology, outside the submitted work. ME reports a charitable grant from the AstraZeneca Young Health Programme; and personal fees from Prudential, outside the submitted work. CJP reports holding stocks in Pfizer, outside the submitted work. JS reports ownership in companies providing services to Itrim, Amgen, Janssen, Novo Nordisk, Eli Lily, Boehringer Ingelheim, Bayer, Pfizer, and AstraZeneca, outside the submitted work. MW reports personal fees from Amgen, Kyowa Kirin and Freeline, outside the submitted work. All other authors declare no competing interests.
