
JOURNAL INFORMATION
==============================
NLM Title Abbreviation: Lancet
Journal ID: Lancet
NLM Journal ID: 2985213R

Lancet (London, England)

ISSN: 0140-6736
EISSN: 1474-547X

ARTICLE INFORMATION
==============================
PMCID: PMC8841598
PMID: 35123692
DOI: 10.1016/S0140-6736(22)00061-7
Article ID: S0140-6736(22)00061-7
Article version: 1
Subjects: Department of Error

Department of Error

Print publication date: 2022 Feb 5
Volume: 399
Issue: 10324
First page: 520
Last page: 520
Copyright: .
Copyright year: 2022
Copyright holder: Elsevier Ltd
License: This is an open access article under the CC BY license (http://creativecommons.org/licenses/by/4.0/).
License URL: https://creativecommons.org/licenses/by/4.0/

Erratum for: Worldwide trends in hypertension prevalence and progress in treatment and control from 1990 to 2019: a pooled analysis of 1201 population-representative studies with 104 million participants 398 10304 11 9 2021 957 980 Lancet (London, England) 10.1016/S0140-6736(21)01330-1 PMC8446938 34450083

==============================
NCD Risk Factor Collaboration (NCD-RisC). Worldwide trends in hypertension prevalence and progress in treatment and control from 1990 to 2019: a pooled analysis of 1201 population-representative studies with 104 million participants. Lancet 2021; 398: 957–80—In this Article, Marialaura Bonaccio, Maria Benedetta Donati, and Francesco Gianfagna have been added to the NCD Risk Factor Collaboration list, and Steinar Krokstad's name has been corrected. These corrections have been made to the online version as of Feb 3, 2022

