# Supplementary material for: Worldwide trends in hypertension prevalence and progress in treatment and control from 1990 to 2019: a pooled analysis of 1201 population-representative studies with 104 million participants
Source: Lancet. 2021 Sep 11;398(10304):957–80. doi: 10.1016/S0140-6736(21)01330-1 (PMC8446938; doi:10.1016/S0140-6736(21)01330-1)
Supplement: Supplementary appendix [file mmc1.pdf]

THE LANCET

Supplementary appendix

This appendix formed part of the original submission and has been peer reviewed. We post it as supplied by the authors.

Supplement to: NCD Risk Factor Collaboration (NCD-RisC). Worldwide trends in hypertension prevalence and progress in treatment and control from 1990 to 2019: a pooled analysis of 1201 population-representative studies with 104 million participants. *Lancet* 2021; published online Aug 24. http://dx.doi.org/10.1016/S0140-6736(21)01330-1.

Table of Contents

Appendix Text 1. Data management. 2

Appendix Text 2. Statistical methods. 4

Appendix Table 1. Data sources used in the analysis. 7

Appendix Table 2. List of analysis regions and "super-regions", and countries in each region. 31

Appendix Table 3. Estimated age-standardised prevalence of hypertension and proportions of people with hypertension who reported a diagnosis, who used treatment, and whose blood pressure was effectively controlled, by country in 2019. 33

Appendix Table 4. Status of hypertension risk factors, and policies and programmes related to hypertension prevention, detection, treatment and control in seven countries with high hypertension treatment rates. 47

Appendix Figure 1. Proportion of people with hypertension who reported a diagnosis, who used treatment, and whose blood pressure was effectively controlled, by region and age group in 2019. 50

Appendix Figure 2. Proportion of people using hypertension treatment whose blood pressure was effectively controlled in 2019, and change from 1990 to 2019. 52

Appendix Figure 3. The relationship between the proportion of people with hypertension who had treatment and whose blood pressure was effectively controlled in 2019. 54

Appendix Figure 4. The relationship between male and female hypertension prevalence, diagnosis, treatment and control in 2019. 56

Appendix Figure 5. Trends in the proportion of people with hypertension who reported a diagnosis, who had treatment, and whose blood pressure was effectively controlled, globally and by region from 1990 to 2019. 58

Appendix Figure 6. Proportion of people with hypertension who reported a diagnosis in 2019, and change from 1990 to 2019. 61

References 64

Appendix Text 1. Data management.

We used a database on cardiometabolic risk factors collated by the Non-Communicable Disease Risk Factor Collaboration (NCD-RisC). Data were obtained from publicly available multi-country and national measurement surveys (e.g., Demographic and Health Surveys (DHS), WHO-STEPwise approach to Surveillance (STEPS) surveys, and those identified via the Inter-University Consortium for Political and Social Research, UK Data Service, and European Health Interview & Health Examination Surveys Database). With the help of World Health Organization (WHO) and its regional and country offices as well as World Heart Federation, we identified and accessed population-based survey data from national health and statistical agencies. We searched and reviewed published studies as detailed previously[1] and invited eligible studies to join NCD-RisC, as did we with data holders from earlier pooled analysis of cardiometabolic risk factors.[2-5]

We carefully checked that each data source meets our inclusion criteria listed below. Potential duplicate data sources were first identified by comparing studies from the same country and year, followed by checking with NCD-RisC members that had provided data about whether the sources from the same country and year were the same or distinct. If two sources were confirmed as duplicates, one was discarded. All NCD-RisC members are also periodically asked to review the list of sources from their country, to verify that the included data meet the inclusion criteria and are not duplicates, and to suggest additional sources. The NCD-RisC database is continuously updated through all the above routes. For each data source, we recorded the study population, sampling approach, years of measurement, and measurement methods. Only population-based data were included, and these were assessed in terms of whether they covered the whole country, multiple sub-national regions, or one or a small number of communities, and whether rural, urban, or both participants were included. All submitted data were checked by at least two independent persons. Questions and clarifications were discussed with NCD-RisC members and resolved before data were incorporated in the database.

Anonymised individual data for participants aged 30 to 79 years from the studies in the NCD-RisC database were reanalysed according to a standardised protocol for 1,157 of the 1,201 studies. We calculated the prevalence of hypertension (as defined in the main paper) and the proportion of people with hypertension who reported a prior hypertension diagnosis, who were taking medication for hypertension and whose blood pressure was controlled, and the associated standard errors by sex and ten-year age groups from 30 to 79 years. We also calculated the proportion of people with hypertension who untreated stage 2 hypertension. All analyses incorporated sample weights and complex survey design, when applicable, in calculating summary statistics, with computer code provided to NCD-RisC members who requested assistance.

Additionally, summary statistics for nationally representative data from sources that were identified but not accessed via the above routes were extracted from published reports. Data were also extracted for two STEPS surveys that were not publicly available. We also included those data from a previous global-data pooling study,[2] when not accessed through the above routes.

We excluded <0.1% of all participants who had implausible systolic blood pressure (defined as <70 mmHg or >270 mmHg) or diastolic blood pressure (<30 mmHg or >150 mmHg). 0.2% of participants reported previous diagnosis with hypertension, were not using medicines, and had blood pressure below 140/90 mmHg. This phenomenon was more common among young adults aged 30-49 years. We assigned them to not having hypertension on the premise that either their hypertension was controlled through lifestyle modification, or their answer may reflect misreporting of general advice about their blood pressure as having had a diagnosis of hypertension.

Appendix Text 2. Statistical methods.

We used a Bayesian hierarchical model to estimate the primary outcomes. The model was applied separately to estimate the following metrics for men and women by country, year, and age: (1) prevalence of hypertension, and the proportion of people with hypertension (2) who reported a prior hypertension diagnosis, (3) who were taking medication for hypertension, (4) whose blood pressure was controlled, and (5) who had untreated stage 2 hypertension. The model is described in detail in statistical[6] and related substantive papers,[1,2] and the computer code for the model is available at www.ncdrisc.org.

In summary, the model had a hierarchical structure in which estimates for each country and year were informed by their own data, if available, and by data from other years in the same country and from other countries, especially those in the same region and super-region, with data for similar time periods. The extent to which estimates for each country-year were influenced by data from other years and other countries depended on whether the country had data, the sample size of the data, whether they were nationally representative, and the within-country and within-region variability of the available data. For the purpose of hierarchical analysis, countries were organised into 21 regions, mostly based on geography and national income. Regions were in turn organised into nine super-regions (Appendix Table 2).

The statistical model included terms that allowed non-linear time trends, specified through a combination of a linear slope and a second order random walk,[7] both modelled hierarchically. We used a cubic spline to allow non-linear age associations. We used a cubic spline model with one knot at 55 years, the mid-point of 30-79-year age range of our study. The mid-age z_h of age group h in study i was used to calculate the age model term γ_i:

$$\gamma_i(z_h) = \gamma_{1i} z_h + \gamma_{2i} z_h^2 + \gamma_{3i} z_h^3 + \gamma_{4i} (z_h - 55)_+^3$$

We adapted the age model that was used in previous NCD-RisC papers[1,2,6] to allow time trends to be different by age. We made this modification because how each of our primary outcomes has changed over time may vary by age.[8,9] Age-dependent trends were specified by interactions with time in the age model. Specifically, each of the spline coefficients (γ_{ki}; k = 1, 2, 3, 4) was allowed to vary both across countries and (linearly) over time, with a hierarchical structure using the equation below, where t is the year study i was conducted; φ is the global intercept; ϕ is the global slope; c, r, s are the country, region and super-region random intercepts, respectively; ω^c, ω^r, ω^s are the country, region and super-region random slopes, respectively; and $j[i]$ is the index of the country study i was from, $l[i]$ is its region, and $m[i]$ is its super-region. We tested models with and without interaction of age and time, including those with time interactions at a subset of hierarchies or for intercepts only. We selected the following model based on Watanabe-Akaike information criterion and visual inspection of results.

$$\gamma_{ki} = \psi_k + \phi_k t_i + c_{kj[i]} + r_{kl[i]} + s_{km[i]} + \omega^c_{kj[i]} t_i + \omega^r_{kl[i]} t_i + \omega^s_{km[i]} t_i$$

The statistical model also accounted for the possibility that hypertension prevalence, diagnosis, treatment and control in sub-national and community samples might differ systematically from nationally representative samples and have larger variation than in national studies. These features were implemented by including data-driven fixed-effect and random-effect terms for sub-national and community data. The fixed effects adjusted for systematic differences between sub-national or community studies and national studies. The random effects allowed national data to have greater influence on the estimates than sub-national or community data with similar sample sizes. Finally, the model accounted for rural-urban differences in the primary outcomes, through the use of fixed effects for rural-only and urban-only studies. These rural and urban fixed effects were weighted by the difference between the study-level and the country-level shares of population living in cities versus rural areas in the year in which the study was done. Data on the proportion of population who live

in urban areas were from the World Urbanization Prospects (2018 revision).[10] The statistical details of the model and the evaluation of its performance are provided in a statistical paper[6] and the appendix of a previous substantive paper.[2]

All analyses were done separately by sex because men and women in the same country can have different levels and trends in hypertension prevalence, diagnosis, treatment and control.[11-13] We fitted the statistical model using Markov chain Monte Carlo (MCMC) implemented in the R programming language.[14] We obtained 50,000 post-burn-in samples (or draws) from the posterior distribution of model parameters. We kept every 10th sample, resulting in 5,000 samples that were in turn used to obtain the posterior distributions of hypertension prevalence, diagnosis, treatment and control. Posterior estimates were made in five-year age groups from 30 to 79 years of age for every year from 1990 to 2019. The uncertainty of the model estimates, represented by their posterior distributions, incorporate: the sampling uncertainty of the data sources used in the study; uncertainty associated with fluctuations in national data because of unmeasured study design and implementation factors that lead to variability beyond that of sampling; additional uncertainty associated with data sources that were not national, because of subgroup variation within each country; and uncertainty due to use of a model to estimate prevalence or proportion by age group, country, and year when data were missing.

We analysed each primary outcome separately. The estimated posterior mean of diagnosis was lower than that of treatment in <0.4% of all country-year-sex-age groups (by an average of 1.8 percentage points); the estimated posterior mean of treatment was lower than that of control in 0.04% of all country-year-sex-age groups (by an average of 0.7 percentage point). In such situations, we adjusted the diagnosis and control rates so that diagnosis was not smaller than treatment and treatment was not smaller than control.

Appendix Table 1. Data sources used in the analysis. All studies had measured data on blood pressure and data on treatment.

	Country	Data years	Survey/Study name/Citation	Level of representative-ness	Rural, urban, or both	Age range used in the global analysis		Sample size used in the global analysis		Information on diagnosis	Type of blood pressure measurement device*	Note
						Female	Male	Female	Male			
1	Afghanistan	2018	STEPS	National	both	30-69	30-69	1,069	1,292	Yes	Standard mercury	
2	Albania	2001	Shapo et al., J Epidemiol Community Health 2003; 57: 734-9	Community	urban	25+	25+	585	535	No	Unknown	1
3	Albania	2008-2009	Demographic and Health Survey Albania 2008-2009	National	both	30-49	30-49	2,081	1,613	Yes	Digital oscillometric	
4	Albania	2017-2018	Demographic and Health Survey Albania 2017-2018	National	both	30-59	30-59	10,100	3,917	Yes	Standard mercury	
5	Algeria	2003	STEPS	Subnational	both	30-64	30-64	1,965	1,336	Yes	Digital oscillometric	
6	Algeria	2007-2009	The ISOR (InSulino-resistance in ORan) Study	Community	urban	30-64	30-64	405	377	Yes	Aneroid	
7	Algeria	2016-2017	STEPS	National	both	30-69	30-69	2,853	2,360	Yes	Digital oscillometric	
8	American Samoa	1994	McGarvey, Pac Health Dialog 2001; 8: 157-62	National	both	30-79	30-79	219	145	Yes	Standard mercury	
9	American Samoa	2004	STEPS	National	both	30-64	30-64	914	818	Yes	Digital oscillometric	
10	Angola	2013-2014	CardioBengo - Population based cardiovascular longitudinal study in Bengo Province, Angola	Community	both	30-65	30-65	866	384	Yes	Digital oscillometric	
11	Argentina	1997	de Sereday et al., Diabetes Metab 2004; 30: 335-9	Subnational	urban	30-59	30-59	849	632	No	Unknown	
12	Argentina	2004-2005	CArdiovascular Risk factors Multiple Evaluation in Latin America (CARMELA)	Community	urban	25-64	25-64	748	734	Yes	Standard mercury	1
13	Argentina	2011-2012	CESCAS Study	Community	urban	30-79	30-79	2,164	1,481	Yes	Unknown	
14	Argentina	2012-2013	Primer estudio sobre el estado nutricional y los hábitos alimentarios de la población adulta de Rosario	Community	urban	30-70	30-70	480	221	Yes	Digital oscillometric	
15	Argentina	2018	Encuesta Nacional de Factores de Riesgo 2018	National	both	30-79	30-79	7,029	5,053	Yes	Unknown	
16	Armenia	2005	Demographic and Health Survey Armenia 2005	National	both	30-49	30-49	3,249	572	Yes	Digital oscillometric	
17	Armenia	2016	STEPS	National	both	30-69	30-69	1,192	438	Yes	Digital oscillometric	
18	Australia	1989	Risk Factor Prevalence Study	National	urban	30-69	30-69	3,826	3,681	Yes	Standard mercury	2
19	Australia	1990-1991	Canberra-Queanbeyan Longitudinal Study of the Elderly	Community	urban	70-79	70-79	276	331	No	Standard mercury	
20	Australia	1994	MONICA, Newcastle	Subnational	urban	35-64	35-64	687	636	Yes	Random-zero mercury	
21	Australia	1994	MONICA, Perth inner	Community	urban	30-64	30-64	322	339	Yes	Standard mercury	
22	Australia	1994	MONICA, Perth outer	Community	urban	30-64	30-64	351	349	Yes	Standard mercury	
23	Australia	1995-1996	National Nutrition Survey 1995	National	both	30-79	30-79	4,252	3,907	No	Standard mercury	
24	Australia	1996-1998	Western Australian AAA Screening Program	Community	urban		65-79		10,867	No	Standard mercury	
25	Australia	1998	Blue Mountain Eyes Study II; Simons et al., Med J Aust 2005; 182: 219-22	Community	urban	50-79	50-79	931	665	No	Unknown	
26	Australia	1999-2000	The Australian Diabetes, Obesity and Lifestyle Study 1999-2000	National	both	30-79	30-79	5,576	4,581	Yes	Unknown	
27	Australia	1999-2003	North West Adelaide Health Study	Community	urban	30-79	30-79	675	620	Yes	Standard mercury	
28	Australia	2004-2005	The Australian Diabetes, Obesity and Lifestyle Study 2004-2005	National	both	30-79	30-79	3,296	2,712	Yes	Digital oscillometric	
29	Australia	2004-2006	North West Adelaide Health Study	Community	urban	30-79	30-79	1,507	1,342	No	Standard mercury	
30	Australia	2008-2010	North West Adelaide Health Study	Community	urban	30-79	30-79	1,195	1,045	No	Standard mercury	
31	Australia	2011-2012	Australian Health Survey	National	both	30-79	30-79	5,313	4,907	No	Digital oscillometric	3
32	Australia	2012	The Australian Diabetes, Obesity and Lifestyle Study 2012	National	both	37-79	37-79	2,339	1,859	Yes	Digital oscillometric	
33	Australia	2014-2015	National Health Survey	National	both	30-79	30-79	4,673	4,085	Yes	Digital oscillometric	
34	Australia	2017-2018	National Health Survey	National	both	30-79	30-79	4,759	4,184	Yes	Digital oscillometric	
35	Austria	1991	CINDI survey Vorarlberg/Austria	Subnational	both	30-64	30-64	663	618	No	Standard mercury	
36	Austria	1998-1999	CINDI survey Vorarlberg/Austria	Subnational	both	30-64	30-64	89	88	No	Standard mercury	
37	Azerbaijan	2006	Demographic and Health Survey Azerbaijan 2006	National	both	30-49	30-59	4,374	1,480	Yes	Digital oscillometric	
38	Azerbaijan	2017	STEPS	National	both	30-69	30-69	1,380	921	Yes	Digital oscillometric	
39	Bahamas	2011-2012	STEPS	National	both	30-64	30-64	786	463	Yes	Digital oscillometric	
40	Bahrain	1995-1996	National Population Register in Bahrain; Al-Mahroos et al., Int J Epidmiol 2000; 29:71-6	National	both	50-69	40-59	872	1,218	No	Standard mercury	
41	Bangladesh	2006	Urban Health Survey	Subnational	urban	35-59	35-59	959	979	Yes	Standard mercury	
42	Bangladesh	2009-2010	STEPS	National	both	30-79	30-79	3,757	3,633	Yes	Aneroid	
43	Bangladesh	2011	Demographic and Health Survey Bangladesh 2011	National	both	30-79	30-79	3,767	3,719	Yes	Digital oscillometric	
44	Bangladesh	2013	STEPS	National	both	30-79	30-79	1,740	1,486	Yes	Digital oscillometric	
45	Bangladesh	2015	An Assessment of BRAC Health Nutrition and Population Programme and Benchmark Survey of Sustainable Development Goal - 2015	National	rural	35-79	35-79	6,374	5,014	Yes	Standard mercury	
46	Bangladesh	2017-2018	Demographic and Health Survey Bangladesh 2017-2018	National	both	30-49		2,845		Yes	Digital oscillometric	
47	Bangladesh	2018	STEPS	National	both	30-69	30-69	3,202	3,020	Yes	Digital oscillometric	
48	Barbados	2011-2013	Health of the Nation (HotN)	National	both	30-79	30-79	648	429	Yes	Digital oscillometric	
49	Belarus	2016-2017	STEPS	National	both	30-69	30-69	2,552	1,755	Yes	Digital oscillometric	
50	Belgium	1990-1993	MONICA, Charleroi	Community	urban	30-64	30-64	302	307	Yes	Random-zero mercury	
51	Belgium	1990-1992	MONICA, Ghent	Community	urban	30-64	30-64	417	451	Yes	Random-zero mercury	
52	Belgium	1991-1994	Flemish Study on Environment, Genes and Health Outcomes	Community	rural	30-79	30-79	370	344	Yes	Standard mercury	
53	Belgium	1992-1995	Flemish Study on Environment, Genes and Health Outcomes	Community	rural	30-79	30-79	288	280	No	Standard mercury	
54	Belgium	1994-1996	BIRNH Elderly: Belgian Interuniversity Research on Nutrition and Health in the Elderly	National	both	65-79	65-79	795	936	Yes	Random-zero mercury	

	Country	Data years	Survey/Study name/Citation	Level of representative-ness	Rural, urban, or both	Age range used in the global analysis		Sample size used in the global analysis		Information on diagnosis	Type of blood pressure measurement device*	Note
						Female	Male	Female	Male			
55	Belgium	1996-1998	Flemish Study on Environment, Genes and Health Outcomes	Community	rural	30-79	30-79	177	182	Yes	Standard mercury	
56	Belgium	1998	Flemish Study on Environment, Genes and Health Outcomes	Community	rural	32-79	32-79	351	313	No	Standard mercury	
57	Belgium	1998-2000	Flemish Study on Environment, Genes and Health Outcomes	Community	rural	30-79	30-79	153	156	No	Standard mercury	
58	Belgium	1999-2001	Flemish Study on Environment, Genes and Health Outcomes	Community	rural	30-79	30-79	160	142	Yes	Standard mercury	
59	Belgium	2001	Flemish Study on Environment, Genes and Health Outcomes	Community	rural	30-78	30-78	161	175	No	Standard mercury	
60	Belgium	2002-2003	Flemish Study on Environment, Genes and Health Outcomes	Community	rural	30-79	30-79	151	144	No	Standard mercury	
61	Belgium	2003	The European Male Ageing Study	Community	both		40-79		443	Yes	Digital oscillometric	
62	Belgium	2002-2005	Flemish Study on Environment, Genes and Health Outcomes	Community	rural	30-79	30-79	199	198	Yes	Standard mercury	
63	Belgium	2005-2008	Flemish Study on Environment, Genes and Health Outcomes	Community	rural	30-79	30-79	75	74	Yes	Standard mercury	
64	Belgium	2008	The European Male Ageing Study	Community	both		40-79		258	Yes	Digital oscillometric	
65	Belgium	2009-2013	Flemish Study on Environment, Genes and Health Outcomes	Community	rural	30-79	30-79	299	301	No	Standard mercury	
66	Belgium	2010-2015	Flemish Study on Environment, Genes and Health Outcomes	Community	rural	30-79	30-79	345	336	No	Standard mercury	
67	Belgium	2018-2019	Belgian Health Examination Survey	National	both	30-79	30-79	523	469	Yes	Digital oscillometric	
68	Belize	2004-2005	CAMDI	National	both	30-79	30-79	784	473	Yes	Digital oscillometric	
69	Benin	2007	STEPS	Community	urban	30-64	30-64	1,090	711	Yes	Digital oscillometric	
70	Benin	2008	STEPS	National	both	30-64	30-64	2,811	2,811	Yes	Digital oscillometric	
71	Benin	2011-2012	Demographic and Health Survey Benin 2011-2012	National	both	30-49	30-64	2,255	2,625	Yes	Digital oscillometric	
72	Benin	2015	STEPS	National	both	30-69	30-69	1,711	1,646	Yes	Digital oscillometric	
73	Benin	2017-2018	Demographic and Health Survey Benin 2017-2018	National	both	30-49	30-64	2,847	3,640	Yes	Digital oscillometric	
74	Bhutan	2007	STEPS	Community	urban	30-74	30-74	1,181	1,005	Yes	Digital oscillometric	
75	Bhutan	2014	STEPS	National	both	30-69	30-69	1,262	875	Yes	Digital oscillometric	
76	Bhutan	2019	STEPS	National	both	30-69	30-69	2,499	1,677	Yes	Digital oscillometric	
77	Bosnia and Herzegovina	2002	Non-communicable disease risk factor survey, Federation of Bosnia and Herzegovina	Subnational	both	30-64	30-64	1,431	1,017	Yes	Standard mercury	
78	Bosnia and Herzegovina	2012	Non-communicable disease risk factor survey, Federation of Bosnia and Herzegovina	Subnational	urban	30-79	30-79	541	436	Yes	Standard mercury	
79	Bosnia and Herzegovina	2012	Non-communicable disease risk factor survey, Federation of Bosnia and Herzegovina	Subnational	rural	30-79	30-79	998	884	Yes	Standard mercury	
80	Botswana	2007	STEPS	National	both	30-64	30-64	1,913	873	Yes	Digital oscillometric	
81	Botswana	2014	STEPS	National	both	30-69	30-69	1,685	744	Yes	Digital oscillometric	
82	Brazil	1995-1996	Cohort study from Porto Alegre	Community	urban	30-79	30-79	428	332	Yes	Aneroid	
83	Brazil	1996-1997	The Bambui Cohort Study of Ageing	Community	urban	60-79	60-79	814	530	Yes	Aneroid	
84	Brazil	1999	Lessa et al., Arq Bras Cardiol 2006; 87: 747-56	Community	urban	30+	30+	650	442	No	Unknown	1
85	Brazil	1999-2000	Prevalence of Risk Factors for Coronary Artery Disease in the State of Rio Grande do Sul	Subnational	urban	30-79	30-79	348	341	Yes	Aneroid	
86	Brazil	1999-2000	Pelotas cross-sectional survey	Community	urban	30-69	30-69	881	611	Yes	Aneroid	
87	Brazil	2003	Hartmann et al., Cad Saude Publica 2007; 23: 1857-66	Community	urban	30-60		737		No	Unknown	
88	Brazil	2003-2005	São Paulo Health & Ageing Study	Community	urban	65-79	65-79	1,034	679	Yes	Digital oscillometric	
89	Brazil	2004	Caju & Virgen das Gracas	Community	rural	30-79	30-79	206	206	No	Standard mercury	
90	Brazil	2004	de Castro et al., Arq Bras Cardiol 2007; 88: 334-9	Community	urban	25+	25+	130	108	No	Unknown	1
91	Brazil	2004-2006	Hearts of Brazil	National	urban	30-79	30-79	512	456	No	Aneroid	
92	Brazil	2005	Syndrome of Obesity and Risk Factors for Cardiovascular Disease Study	Community	urban	30-79	30-79	840	513	No	Digital oscillometric	
93	Brazil	2008	The Bambui Cohort Study of Ageing	Community	urban	71-79	71-79	315	188	Yes	Aneroid	
94	Brazil	2008	Caju & Virgen das Gracas	Community	rural	30-79	30-79	205	198	Yes	Standard mercury	
95	Brazil	2009-2010	EpiFloripa Cohort Study of Ageing - Wave 1	Community	urban	60-79	60-79	918	532	Yes	Digital oscillometric	
96	Brazil	2009-2010	EpiFloripa Adults Cohort Study	Community	urban	30-59	30-59	669	498	Yes	Digital oscillometric	
97	Brazil	2010	San Pedro	Community	rural	30-79	30-79	157	104	Yes	Standard mercury	
98	Brazil	2012-2013	Prevalence of Leptin Polymorphism Gln223Arg	Community	urban	30-79	30-79	369	201	Yes	Digital oscillometric	
99	Brazil	2010-2015	Baependi Heart Study	Community	rural	30-79	30-79	997	713	Yes	Digital oscillometric	
100	Brazil	2013	Pesquisas Nacional de Saude	National	both	30-79	30-79	23,956	17,882	Yes	Unknown	
101	Brazil	2011-2014	Profile of Risk Factors for Coronary Arterial Disease in Rio Grade do Sul - Revaluation After 10 Years	Subnational	urban	30-79	30-79	374	281	Yes	Aneroid	
102	Brazil	2013-2014	EpiFloripa Cohort Study of Ageing - Wave 2	Community	urban	63-79	63-79	586	328	Yes	Digital oscillometric	
103	Brazil	2015-2016	Brazilian Longitudinal Study of the Elderly Health and Wellness	National	both	50-79	50-79	4,725	3,745	Yes	Digital oscillometric	
104	Brazil	2017	Effectiveness of interventions for health promotion in frail older adults with chronic non-communicable diseases in primary healthcare in Recife: a randomized community trial	Community	urban	60-79	60-79	487	134	Yes	Digital oscillometric	
105	Brazil	2016-2017	Study in Presidente Prudente	Community	urban	30-79	30-79	388	246	Yes	Digital oscillometric	
106	Brazil	2017-2018	EpiFloripa Cohort Study of Ageing - Wave 3	Community	urban	60-79	60-79	482	294	Yes	Digital oscillometric	
107	Brazil	2018-2019	Epidemiology in the health (Santo Anastácio Edition)	Community	urban	30-79	30-79	105	71	Yes	Digital oscillometric	
108	Brunei Darussalam	2010-2011	National Health And Nutritional Status Survey (NHANSS)	National	both	30-75	30-75	576	444	Yes	Digital oscillometric	
109	Brunei Darussalam	2015-2016	National Non-Communicable Diseases Survey (NNCDS)	National	both	30-69	30-69	866	669	Yes	Digital oscillometric	

	Country	Data years	Survey/Study name/Citation	Level of representative-ness	Rural, urban, or both	Age range used in the global analysis		Sample size used in the global analysis		Information on diagnosis	Type of blood pressure measurement device*	Note
						Female	Male	Female	Male			
110	Burkina Faso	2013	STEPS	National	both	30-64	30-64	1,437	1,374	Yes	Digital oscillometric	
111	Cabo Verde	2007	STEPS	National	both	30-64	30-64	952	542	Yes	Digital oscillometric	
112	Cabo Verde	2019	STEPS	National	both	30-69	30-69	2,038	1,373	Yes	Digital oscillometric	
113	Cambodia	2010	STEPS	National	both	30-64	30-64	2,881	1,581	Yes	Digital oscillometric	
114	Cameroon	2003	STEPS	Subnational	urban	30-79	30-79	2,706	1,802	Yes	Digital oscillometric	
115	Cameroon	2007	Cameroon Burden of Diabetes - Second Survey	Subnational	urban	30-79	30-79	3,264	2,493	Yes	Digital oscillometric	
116	Cameroon	2014-2015	Cardiovascular risk factors screening in urban and rural areas in the Far-North Region Cameroon	Subnational	both	30-79	30-79	255	306	Yes	Digital oscillometric	
117	Canada	1986-1992	Canada Heart Health Survey	National	both	30-74	30-74	7,858	7,662	Yes	Standard mercury	2
118	Canada	1995	MONICA, Halifax	Community	both	30-64	30-64	261	250	Yes	Aneroid	
119	Canada	2006	Ontario Survey of the Prevalence and Control of Hypertension; Leenen et al., CMAJ 2008; 178: 1441-9	Community	urban	20-79	20-79	1,540	1,452	No	Unknown	1
120	Canada	2007-2009	Canadian Health Measures Survey, Cycle 1	National	both	40-79	30-79	1,204	1,424	Yes	Digital oscillometric	
121	Canada	2009-2011	Canadian Health Measures Survey, Cycle 2	National	both	30-79	30-79	1,727	1,469	Yes	Digital oscillometric	
122	Canada	2012-2013	Canadian Health Measures Survey, Cycle 3	National	both	30-79	30-79	1,432	1,385	Yes	Digital oscillometric	
123	Canada	2014-2015	Canadian Health Measures Survey, Cycle 4	National	both	30-79	30-79	1,402	1,411	Yes	Digital oscillometric	
124	Canada	2016-2017	Canadian Health Measures Survey, Cycle 5	National	both	30-79	30-79	1,404	1,393	Yes	Digital oscillometric	
125	Canada	2018-2019	Canadian Health Measures Survey, Cycle 6	National	both	40-79	30-79	1,053	1,450	Yes	Digital oscillometric	
126	Central African Republic	2010	STEPS	Subnational	both	30-64	30-64	1,531	1,471	Yes	Digital oscillometric	
127	Central African Republic	2017	STEPS	Subnational	both	25-64	25-64	1,810	1,064	Yes	Digital oscillometric	1
128	Chad	2008	STEPS	Community	urban	30-64	30-64	608	722	Yes	Digital oscillometric	
129	Chile	2003	Encuesta Nacional de Salud	National	both	30-79	30-79	1,433	1,098	Yes	Digital oscillometric	
130	Chile	2004-2005	CArdiovascular Risk factors Multiple Evaluation in Latin America (CARMELA)	Community	urban	25-64	25-64	872	783	Yes	Standard mercury	1
131	Chile	2009-2010	Encuesta Nacional de Salud	National	both	30-79	30-79	2,123	1,433	Yes	Digital oscillometric	
132	Chile	2011-2012	CESCAS Study	Community	urban	30-79	30-79	926	818	Yes	Unknown	
133	Chile	2016-2017	Encuesta Nacional de Salud	National	both	30-79	30-79	2,617	1,431	Yes	Digital oscillometric	
134	China	1991	China National Hypertension Survey Epidemiology Follow-up Study	National	both	40-79	40-79	83,909	81,873	Yes	Standard mercury	
135	China	1991	Sino-MONICA Shanghai	Community	rural	30-64	30-64	624	564	Yes	Standard mercury	
136	China	1991	Health, health risk and quality of life in a Hong Kong Chinese elderly cohort	Community	both	70-79	70-79	572	596	No	Standard mercury	
137	China	1991	China Health and Nutrition Study	National	both	30-79	30-79	2,868	2,586	Yes	Standard mercury	4
138	China	1990-1991	China Prospective Study	National	both		40-79		230,546	Yes	Unknown	
139	China	1991	China National Hypertension Survey	National	both	30-74	30-74	325,838	291,240	No	Standard mercury	
140	China	1992	Sino-MONICA Sichuan	Community	both	30-64	30-64	447	542	Yes	Standard mercury	
141	China	1991-1992	Fangshan Cohort Study	Community	urban	34-79	34-79	1,742	875	No	Standard mercury	
142	China	1992	Huashan Study	Community	urban	35-75	35-75	962	893	No	Standard mercury	
143	China	1993	Sino-MONICA Anhui	Community	urban	30-64	30-64	156	167	Yes	Standard mercury	
144	China	1993	Sino-MONICA Beijing	Community	both	30-64	30-64	750	564	Yes	Standard mercury	
145	China	1993	Sino-MONICA Jiangsu	Community	urban	30-64	30-64	300	408	Yes	Standard mercury	
146	China	1993	Sino-MONICA Liaoning	Community	both	30-64	30-64	460	437	Yes	Standard mercury	
147	China	1993	China Health and Nutrition Study	National	both	30-79	30-79	2,900	2,585	Yes	Standard mercury	4
148	China	1995-1996	Hong Kong Cardiovascular Risk Factor Prevalence Study 1995-1996	Community	both	30-74	30-74	1,380	1,278	Yes	Standard mercury	
149	China	1997	INTERMAP, Beijing	Community	rural	40-59	40-59	139	133	Yes	Random-zero mercury	
150	China	1997	INTERMAP, Guangxi	Community	rural	40-59	40-59	138	140	Yes	Random-zero mercury	
151	China	1997	China Health and Nutrition Study	National	both	30-79	30-79	3,394	3,078	Yes	Standard mercury	4
152	China	1997	INTERMAP, Shanxi	Community	rural	40-59	40-59	146	143	Yes	Random-zero mercury	
153	China	2000	China Health and Nutrition Study	National	both	30-79	30-79	3,944	3,531	Yes	Standard mercury	4
154	China	2000-2001	The International Collaborative Study of Cardiovascular Disease in Asia	National	both	35-74	35-74	8,008	7,515	Yes	Standard mercury	
155	China	2002	China National Nutrition and Health Survey	National	both	30-79	30-79	62,231	52,711	Yes	Standard mercury	
156	China	2004	China Health and Nutrition Study	National	both	30-79	30-79	4,141	3,714	Yes	Standard mercury	4
157	China	2005	Pang et al., Intern Med 2008; 47: 893-7	Community	rural	35+	35+	22,963	22,962	No	Unknown	1
158	China	2006	Beijing Eye Study	Community	both	45-79	45-79	1,785	1,319	Yes	Standard mercury	
159	China	2006	CHEAPS Study; Dong et al., Indian J Med Res 2008; 128: 122-7	Subnational	rural	35-74	35-74	22,012	21,680	No	Unknown	
160	China	2006	China Health and Nutrition Study	National	both	30-79	30-79	4,254	3,745	Yes	Standard mercury	4
161	China	2006	Dong et al., Hypertens Res 2007; 30: 951-8	Subnational	rural	35-74	35-74	14,048	14,395	No	Unknown	
162	China	2004-2008	China Kadoorie Biobank baseline survey	Subnational	rural	35-74	35-74	162,842	115,782	Yes	Digital oscillometric	
163	China	2004-2008	China Kadoorie Biobank baseline survey	Subnational	urban	35-74	35-74	132,856	89,216	Yes	Digital oscillometric	
164	China	2007-2008	China National Diabetes and Metabolic Disorders Study	National	both	30+	30+	18,294	17,715	Yes	Standard mercury	1

	Country	Data years	Survey/Study name/Citation	Level of representative-ness	Rural, urban, or both	Age range used in the global analysis		Sample size used in the global analysis		Information on diagnosis	Type of blood pressure measurement device*	Note
						Female	Male	Female	Male			
165	China	2008-2010	Fangshan Family-based Ischemic Stroke Study in China (FISSIC) program	Community	rural	40-79	40-79	37,227	19,868	Yes	Standard mercury	
166	China	2009	China Health and Nutrition Study	National	both	30-79	30-79	4,350	3,931	Yes	Standard mercury	4
167	China	2007-2010	SAGE	National	both	50-79	50-79	6,235	5,427	Yes	Digital oscillometric	
168	China	2009-2010	China National Survey of Chronic Kidney Disease	National	both	30-79	30-79	20,307	13,669	Yes	Standard mercury	
169	China	2010	China Noncommunicable Disease Surveillance	National	both	30-79	30-79	45,331	37,327	No	Digital oscillometric	
170	China	2011-2012	China Health and Retirement Longitudinal Study (CHARLS), baseline survey	National	both	45-79	45-79	6,761	6,172	Yes	Digital oscillometric	
171	China	2011	China Health and Nutrition Study	National	both	30-79	30-79	5,823	5,159	Yes	Standard mercury	4
172	China	2011	Beijing Eye Study	Community	both	50-79	50-79	1,658	1,199	Yes	Standard mercury	
173	China	2010-2012	China National Nutrition and Health Surveillance	National	both	45-59	45-59	25,904	19,071	Yes	Standard mercury	
174	China	2012	China Health and Retirement Longitudinal Study (CHARLS), wave 2 pilot survey	Subnational	both	45-79	45-79	902	814	Yes	Digital oscillometric	
175	China	2013	China Health and Retirement Longitudinal Study (CHARLS), wave 2 survey	National	both	45-79	45-79	6,202	5,577	Yes	Digital oscillometric	
176	China	2012-2013	The Kailuan Study	Community	urban	30-79	30-79	19,009	71,824	Yes	Standard mercury	
177	China	2012-2015	Shanghai Women's Health Study	Community	urban	52-79		37,813		No	Unknown	
178	China	2012-2015	China Hypertension Survey	National	both	25-74	25-74	190,976	175,027	Yes	Digital oscillometric	1
179	China	2012-2015	Shanghai Men's Health Study	Community	urban		47-79		30,003	No	Unknown	
180	China	2015	China Health and Retirement Longitudinal Study (CHARLS), wave 4 survey	National	both	45-79	45-79	7,014	6,446	Yes	Digital oscillometric	
181	China	2014-2015	The Kailuan Study	Community	urban	30-79	30-79	16,860	60,876	Yes	Digital oscillometric	
182	China	2015	China Health and Nutrition Study	National	both	30-79	30-79	4,493	3,864	Yes	Standard mercury	4
183	China	2014-2017	China Patient-Centered Evaluative Assessment of Cardiac Events (PEACE) Million Persons Project	Subnational	both	35-75	35-75	1,035,141	703,745	Yes	Digital oscillometric	
184	Colombia	2002	The Santa Fe Study (Santa Fe)	Community	urban	30-69	30-69	380	206	No	Unknown	
185	Colombia	2004-2005	CArdiovascular Risk factors Multiple Evaluation in Latin America (CARMELA)	Community	urban	25-64	25-64	815	738	Yes	Standard mercury	1
186	Colombia	2007	Encuesta Nacional de Salud	National	both	30-69	30-69	5,176	3,535	Yes	Digital oscillometric	
187	Colombia	2010	STEPS	Subnational	both	30-64	30-64	815	612	Yes	Digital oscillometric	
188	Colombia	2015	STEPS	Subnational	both	30-64	30-64	856	584	Yes	Digital oscillometric	
189	Comoros	2011	STEPS	National	both	30-64	30-64	2,876	1,268	Yes	Digital oscillometric	
190	Congo	2004	STEPS	Community	urban	30-64	30-64	585	640	Yes	Digital oscillometric	
191	Cook Islands	2003	STEPS	National	both	30-64	30-64	849	831	Yes	Digital oscillometric	
192	Cook Islands	2013-2015	STEPS	National	both	30-64	30-64	355	382	Yes	Digital oscillometric	
193	Costa Rica	2004	CAMDI	Community	urban	30-79	30-79	562	293	Yes	Digital oscillometric	
194	Costa Rica	2004-2006	Costa Rican Longevity and Healthy Aging Study Pre-1945 Cohort Wave 1	National	both	60-79	60-79	942	828	Yes	Digital oscillometric	
195	Costa Rica	2006-2008	Costa Rican Longevity and Healthy Aging Study Pre-1945 Cohort Wave 2	National	both	62-79	62-79	762	668	Yes	Digital oscillometric	
196	Costa Rica	2009-2010	Costa Rican Longevity and Healthy Aging Study Pre-1945 Cohort Wave 3	National	both	64-79	64-79	576	480	Yes	Digital oscillometric	
197	Costa Rica	2010	Costa Rican National Cardiovascular Risk Factors Survey, 2010	National	both	30-79	30-79	1,965	729	Yes	Standard mercury	
198	Costa Rica	2010-2011	Costa Rican Longevity and Healthy Aging Study 1945-1955 Cohort Wave 1	National	both	54-66	54-66	1,688	1,079	Yes	Digital oscillometric	
199	Costa Rica	2012-2014	Costa Rican Longevity and Healthy Aging Study 1945-1955 Cohort Wave 2	National	both	56-68	56-68	1,503	893	Yes	Digital oscillometric	
200	Costa Rica	2014	Costa Rican National Cardiovascular Risk Factors Survey, 2014	National	both	30-79	30-79	1,807	810	Yes	Standard mercury	
201	Cote d'Ivoire	2005	STEPS	Subnational	urban	30-64	30-64	597	551	Yes	Digital oscillometric	
202	Cote d'Ivoire	2005	STEPS	Subnational	rural	30-64	30-64	561	561	Yes	Digital oscillometric	
203	Croatia	2003	Croatian Adult Health Survey 2003	National	both	30-79	30-79	5,191	2,527	Yes	Standard mercury	
204	Croatia	2002-2007	Epidemiology of arterial hypertension in Croatia (EH-UH)	National	both	30-79	30-79	535	398	Yes	Standard mercury	
205	Croatia	2008	Croatian Adult Health Cohort Study (CroHort)	Subnational	both	30-79	30-79	1,823	848	Yes	Standard mercury	
206	Croatia	2008	Endemic Nephropathy and Arterial Hypertension (ENAH)	Subnational	rural	30-79	30-79	398	230	No	Digital oscillometric	
207	Croatia	2010	Endemic Nephropathy and Arterial Hypertension (ENAH)	Subnational	rural	30-79	30-79	262	158	No	Digital oscillometric	
208	Croatia	2015	Endemic Nephropathy and Arterial Hypertension (ENAH) Follow-up Study	Subnational	rural	30-79	30-79	423	205	No	Digital oscillometric	
209	Cuba	1999-2000	The Survey on Health, Well-Being, and Aging in Latin America and the Caribbean (SABE)	Community	urban	60-79	60-79	835	531	Yes	Unknown	5
210	Cuba	2010	National Risk Factor Survey	National	both	30-79	30-79	2,915	2,439	Yes	Standard mercury	
211	Cuba	2011	Noncommunicable disease risk factor in Cienfuegos	Community	urban	30-74	30-74	704	487	Yes	Standard mercury	
212	Czechia	1988	MONICA	National	both	30-64	30-64	1,280	1,231	Yes	Standard mercury	2
213	Czechia	1992	MONICA	National	both	30-64	30-64	1,094	1,006	Yes	Standard mercury	
214	Czechia	1997-1998	Czech post-MONICA	National	both	30-64	30-64	1,498	1,379	Yes	Standard mercury	
215	Czechia	1998-2002	Health, Lifestyle and the Environment	National	urban	45-54	45-54	2,033	1,527	Yes	Standard mercury	
216	Czechia	2000-2001	Czech post-MONICA	National	both	30-64	30-64	1,521	1,480	Yes	Standard mercury	
217	Czechia	2002-2005	Health, Alcohol and Psychosocial factors In Eastern Europe (HAPIEE)	Subnational	urban	44-72	44-72	3,910	3,293	Yes	Digital oscillometric	
218	Czechia	2004-2005	Health, Lifestyle and the Environment	National	urban	45-54	45-54	1,060	758	Yes	Standard mercury	
219	Czechia	2006-2009	Czech post-MONICA	National	both	30-64	30-64	1,703	1,579	Yes	Standard mercury	
220	Czechia	2009	Health, Lifestyle and the Environment	National	urban	45-54	45-54	444	305	Yes	Standard mercury	

	Country	Data years	Survey/Study name/Citation	Level of representative-ness	Rural, urban, or both	Age range used in the global analysis		Sample size used in the global analysis		Information on diagnosis	Type of blood pressure measurement device*	Note
						Female	Male	Female	Male			
221	Czechia	2014-2015	European Heath Examination Survey	National	both	30-64	30-64	653	433	Yes	Digital oscillometric	
222	Czechia	2015-2018	MONICA	National	both	30-65	30-65	1,270	1,178	Yes	Standard mercury	
223	Denmark	1991-1992	MONICA, Glostrup	Subnational	urban	30-61	30-61	668	662	Yes	Standard mercury	
224	Denmark	1991-1994	Copenhagen City Heart Study	Subnational	urban	30-79	30-79	4,995	3,877	No	Random-zero mercury	
225	Denmark	1993-1994	MONICA I, 10-years re-examination (semi-MONICA)	Community	urban	41-72	41-72	1,312	1,322	Yes	Unknown	
226	Denmark	1993-1997	EPIC Aarhus	Community	urban	50-65	50-65	7,583	6,817	Yes	Digital oscillometric	
227	Denmark	1993-1997	EPIC Copenhagen	Community	urban	50-65	50-65	19,050	15,900	Yes	Digital oscillometric	
228	Denmark	2001-2003	Copenhagen City Heart Study	Subnational	urban	30-79	30-79	2,763	2,169	No	Random-zero mercury	
229	Denmark	2003-2004	Copenhagen General Population Study 1	Subnational	urban	30-79	30-79	5,079	4,477	No	Digital oscillometric	
230	Denmark	2005	Copenhagen General Population Study 1	Subnational	urban	30-79	30-79	5,475	4,771	No	Digital oscillometric	
231	Denmark	2006	Copenhagen General Population Study 1	Subnational	urban	30-79	30-79	4,441	4,705	No	Digital oscillometric	
232	Denmark	2006-2008	The Health2006 Cohort	Subnational	urban	30-71	30-71	1,706	1,427	Yes	Standard mercury	
233	Denmark	2007	Copenhagen General Population Study 1	Subnational	urban	30-79	30-79	6,467	3,706	No	Digital oscillometric	
234	Denmark	2008	Copenhagen General Population Study 1	Subnational	urban	30-79	30-79	6,083	4,417	No	Digital oscillometric	
235	Denmark	2007-2008	The Danish Health Examination Survey 2007-2008	National	both	30-79	30-79	9,288	6,492	Yes	Digital oscillometric	
236	Denmark	2009	Copenhagen General Population Study 1	Subnational	urban	30-79	30-79	4,960	3,955	No	Digital oscillometric	
237	Denmark	2010	Copenhagen General Population Study 1	Subnational	urban	30-79	30-79	4,518	3,625	No	Digital oscillometric	
238	Denmark	2011	Copenhagen General Population Study 1	Subnational	urban	30-79	30-79	5,596	4,740	No	Digital oscillometric	
239	Denmark	2011-2012	The Health2006 cohort - 5-year follow-up	Subnational	urban	30-76	30-76	1,177	1,005	Yes	Standard mercury	
240	Denmark	2012	Copenhagen General Population Study 1	Subnational	urban	30-79	30-79	5,082	4,136	No	Digital oscillometric	
241	Denmark	2013	Copenhagen General Population Study 1	Subnational	urban	30-79	30-79	4,462	3,762	No	Digital oscillometric	
242	Denmark	2012-2015	The Danish study of Functional Disorders (DanFunD)	Subnational	urban	30-72	30-72	3,632	3,129	Yes	Aneroid	
243	Denmark	2014-2015	Copenhagen General Population Study 1	Subnational	urban	30-79	30-79	2,661	2,065	No	Digital oscillometric	
244	Denmark	2017	Copenhagen General Population Study 2	Subnational	urban	30-79	30-79	4,054	2,930	No	Digital oscillometric	
245	Dominica	2007	STEPS	National	both	30-64	30-64	388	283	Yes	Digital oscillometric	
246	Dominican Republic	1996-1998	Estudio factores de riesgo cardiovascular y sindrome metabolico en la Republica Dominicana I (EFRICARD I)	National	both	30-75	30-75	3,503	1,859	Yes	Aneroid	
247	Dominican Republic	2010-2012	Estudio factores de riesgo cardiovascular y sindrome metabolico en la Republica Dominicana II (EFRICARD II)	National	both	30-75	30-75	2,561	1,346	Yes	Aneroid	
248	DR Congo	2005	STEPS	Subnational	urban	30-79	30-79	606	394	Yes	Digital oscillometric	
249	Ecuador	2004-2005	CArdiovascular Risk factors Multiple Evaluation in Latin America (CARMELA)	Community	urban	25-64	25-64	825	813	Yes	Standard mercury	1
250	Ecuador	2009-2010	National Survey on Health, Well-Being, and Aging (SABE)	National	both	60-79	60-79	2,156	1,978	Yes	Standard mercury	
251	Ecuador	2011-2013	Encuesta Nacional de Salud y Nutrición (ENSANUT)	National	both	30-59	30-59	10,674	8,096	Yes	Digital oscillometric	
252	Ecuador	2018	STEPS	National	both	30-69	30-69	1,944	1,389	Yes	Digital oscillometric	
253	Egypt	1991-1993	The Egyptian National Hypertension Study	National	both	30-79	30-79	3,269	2,513	Yes	Standard mercury	
254	Egypt	2002	National Survey of Smoking, Obesity, Blood Pressure and Blood Glucose	National	both	30-79	30-79	1,713	1,462	Yes	Standard mercury	
255	Egypt	2003-2004	Metabolic and cardiovascular risk profiles and hepatitis C virus infection in rural Egypt	Community	rural	30-79	30-79	379	275	Yes	Standard mercury	
256	Egypt	2005	STEPS	National	both	30-65	30-65	2,352	2,501	Yes	Digital oscillometric	
257	Egypt	2007-2009	Hepatitis C infection and clearance: impact on atherosclerosis and cardiometabolic risk factors	Community	rural	35-79	35-79	833	633	Yes	Digital oscillometric	
258	Egypt	2008	Demographic and Health Survey Egypt 2008	National	both	30-59	30-59	3,226	2,856	Yes	Digital oscillometric	
259	Egypt	2011	STEPS	National	both	30-65	30-65	2,131	1,243	Yes	Digital oscillometric	
260	Egypt	2015	Demographic and Health Survey Egypt 2015	National	both	30-59	30-59	4,980	4,325	Yes	Digital oscillometric	
261	Egypt	2017	STEPS	National	both	30-69	30-69	2,958	1,639	Yes	Digital oscillometric	
262	El Salvador	2004	CAMDI	Community	urban	30-79	30-79	624	287	Yes	Unknown	
263	El Salvador	2014-2015	Encuesta Nacional de Enfermedades Crónicas (ENECA-ELS)	National	both	30-79	30-79	2,157	1,220	Yes	Digital oscillometric	
264	Eritrea	2004	STEPS	National	both	30-64	30-64	747	774	Yes	Digital oscillometric	
265	Eritrea	2010	STEPS	National	both	30-74	30-74	3,493	1,566	Yes	Digital oscillometric	
266	Estonia	2003	The European Male Ageing Study	Community	both		40-79		413	Yes	Digital oscillometric	
267	Estonia	2002-2004	Estonian Biobank	National	both	30-79	30-79	5,334	2,444	Yes	Digital oscillometric	
268	Estonia	2008	The European Male Ageing Study	Community	both		40-79		205	Yes	Digital oscillometric	
269	Estonia	2007-2008	Estonian Biobank	National	both	30-79	30-79	9,609	4,149	Yes	Digital oscillometric	
270	Estonia	2009-2010	Estonian Biobank	National	both	30-79	30-79	9,952	5,292	Yes	Digital oscillometric	
271	Eswatini	2007	STEPS	National	both	30-64	30-64	668	323	Yes	Digital oscillometric	
272	Eswatini	2014	STEPS	National	both	30-69	30-69	1,245	603	Yes	Digital oscillometric	
273	Ethiopia	2004	STEPS; Tesfaye et al., Hum Hypertens 2007; 21: 28-37	Subnational	both	25-64	25-64	2,284	1,763	No	Unknown	1
274	Ethiopia	2006	Surveillance of NCD Risk Factors in Addis Ababa (STEPS)	Subnational	urban	30-64	30-64	1,865	1,205	Yes	Digital oscillometric	

	Country	Data years	Survey/Study name/Citation	Level of representative-ness	Rural, urban, or both	Age range used in the global analysis		Sample size used in the global analysis		Information on diagnosis	Type of blood pressure measurement device*	Note
						Female	Male	Female	Male			
275	Ethiopia	2015	STEPS	National	both	30-69	30-69	3,138	2,492	Yes	Digital oscillometric	
276	Fiji	2002	STEPS	National	both	30-64	30-64	2,497	1,695	Yes	Digital oscillometric	
277	Fiji	2011	STEPS	National	both	30-64	30-64	1,237	974	Yes	Digital oscillometric	
278	Finland	1992	The National FINRISK Study	Subnational	both	30-64	30-64	2,837	2,551	Yes	Standard mercury	
279	Finland	1991-1993	Kuopio Ischaemic Heart Disease Risk Factor Study	Subnational	both		46-65		1,024	Yes	Standard mercury	
280	Finland	1997	North Finland Birth Cohort 1966	Community	both	30-31	30-31	149	1,035	Yes	Digital oscillometric	
281	Finland	1996-1998	Oulu 35 Study	Community	urban	60-63	60-63	334	233	Yes	Standard mercury	
282	Finland	1996-1998	Savitaipale Study, Baseline	Community	rural	40-66	40-66	555	548	Yes	Standard mercury	
283	Finland	1997	The National FINRISK Study	National	both	30-74	30-74	3,654	3,721	Yes	Standard mercury	
284	Finland	1998-2001	Kuopio Ischaemic Heart Disease Risk Factor Study	Subnational	both	53-73	53-73	920	832	Yes	Standard mercury	
285	Finland	2001	Young Finns Study 2001 urban	National	urban	30-39	30-39	491	440	Yes	Random-zero mercury	
286	Finland	2001	Young Finns Study 2001 rural	National	rural	30-39	30-39	304	261	Yes	Random-zero mercury	
287	Finland	2000-2001	Health 2000 Survey	National	both	30-79	30-79	3,228	2,817	Yes	Standard mercury	
288	Finland	2001-2003	Oulu 45 Study	Community	urban	55-58	55-58	526	418	Yes	Digital oscillometric	
289	Finland	2002	The National FINRISK Study	National	both	30-74	30-74	4,124	3,607	Yes	Standard mercury	
290	Finland	2005-2008	Kuopio Ischaemic Heart Disease Risk Factor Study	Subnational	both	60-79	60-79	617	1,196	Yes	Standard mercury	
291	Finland	2007	Oulu 35 Study	Community	urban	72-72	72-72	230	157	Yes	Standard mercury	
292	Finland	2007	Young Finns Study 2007 rural	National	rural	30-45	30-45	435	375	Yes	Random-zero mercury	
293	Finland	2007	The National FINRISK Study	National	both	30-74	30-74	2,946	2,653	Yes	Standard mercury	
294	Finland	2007	Young Finns Study 2007 urban	National	urban	30-45	30-45	715	586	Yes	Random-zero mercury	
295	Finland	2008	Control group for Finnish male former elite athletes	National	both		61-79		194	Yes	Standard mercury	
296	Finland	2007-2008	Savitaipale Study, Follow-up	Community	rural	51-75	51-75	467	413	No	Standard mercury	
297	Finland	2011	Young Finns Study 2011 urban	National	urban	34-49	34-49	610	487	Yes	Random-zero mercury	
298	Finland	2011	Young Finns Study 2011 rural	National	rural	34-49	34-49	407	350	Yes	Random-zero mercury	
299	Finland	2012	The National FINRISK Study	National	both	30-74	30-74	2,733	2,522	Yes	Standard mercury	
300	Finland	2012	North Finland Birth Cohort 1966	Community	both	45-47	45-47	2,692	1,683	Yes	Digital oscillometric	
301	Finland	2011-2012	Health 2011 Survey	National	both	30-79	30-79	2,280	1,857	Yes	Standard mercury	
302	Finland	2017	The FinHealth Survey	National	both	30-79	30-79	2,669	2,339	Yes	Standard mercury	
303	France	1988-1991	MONICA, Toulouse	Subnational	both		35-64		586	Yes	Random-zero mercury	
304	France	1994-1996	MONICA, Toulouse	Subnational	both	35-64	35-64	566	609	Yes	Random-zero mercury	
305	France	1995-1997	MONICA, Strasbourg	Subnational	both	35-64	35-64	530	524	Yes	Digital oscillometric	
306	France	1995-1997	MONICA, Lille	Community	urban	36-66	36-67	594	597	Yes	Standard mercury	
307	France	1999-2001	The Three City Study	Community	urban	65-79	65-79	4,444	2,902	Yes	Digital oscillometric	
308	France	2001-2003	The Three City Study	Community	urban	66-79	66-79	3,600	2,329	Yes	Digital oscillometric	
309	France	2003-2005	The Three City Study	Community	urban	68-79	68-79	2,815	1,821	Yes	Digital oscillometric	
310	France	2004-2006	National Monitoring of Arterial Risk in Lille (MONA LISA Lille)	Community	urban	35-75	35-75	796	797	Yes	Digital oscillometric	
311	France	2005-2007	National Monitoring of Arterial Risk in Toulouse (MONA LISA Toulouse)	Subnational	both	35-74	35-74	792	828	Yes	Digital oscillometric	
312	France	2005-2007	National Monitoring of Arterial Risk in Bas-Rhin (MONA LISA Bas-Rhin)	Subnational	both	35-74	35-74	785	781	Yes	Digital oscillometric	
313	France	2006-2007	Etude Nationale Nutrition Santé	National	both	30-74	30-74	1,158	686	Yes	Digital oscillometric	
314	France	2006-2008	The Three City Study	Community	urban	72-79	72-79	633	447	Yes	Digital oscillometric	
315	France	2008-2010	The Three City Study	Community	urban	73-79	73-79	557	369	Yes	Digital oscillometric	
316	France	2010-2012	The Three City Study	Community	urban	76-79	76-79	249	196	Yes	Digital oscillometric	
317	France	2011-2013	Enquête LIttoral Souffle Air Biologie EnvironnemenT (ELISABET) Lille	Community	urban	40-64	40-64	852	751	Yes	Digital oscillometric	
318	France	2011-2013	Enquête LIttoral Souffle Air Biologie EnvironnemenT (ELISABET) Dunkerque	Community	urban	40-64	40-64	793	750	Yes	Digital oscillometric	
319	France	2014-2016	Esteban	National	both	30-74	30-74	1,082	889	Yes	Digital oscillometric	
320	French Polynesia	2010	STEPS	National	both	30-64	30-64	980	752	Yes	Digital oscillometric	
321	Gabon	2009	STEPS	Subnational	urban	30-64	30-64	908	732	Yes	Digital oscillometric	
322	Gambia	2010	STEPS	National	both	30-64	30-64	1,291	1,347	Yes	Digital oscillometric	
323	Georgia	2010	STEPS	National	both	30-64	30-64	3,846	1,454	Yes	Digital oscillometric	
324	Georgia	2016	STEPS	National	both	30-69	30-69	2,439	988	Yes	Standard mercury	
325	Germany	1989-1990	MONICA, Augsburg	Community	both	30-64	30-64	1,742	1,709	Yes	Random-zero mercury	
326	Germany	1991-1992	MONICA, Bremen Center/South/East	Community	urban	30-69	30-69	472	463	Yes	Random-zero mercury	
327	Germany	1991-1992	MONICA, Bremen North/West	Community	urban	30-69	30-69	542	461	Yes	Random-zero mercury	
328	Germany	1991-1992	German Cardiovascular Prevention Study (GCP) - National Health Survey 1991	Subnational	both	30-69	30-69	2,168	1,932	Yes	Standard mercury	
329	Germany	1991-1992	First National Examination of life conditions, Environment and Health in East Germany 1991/92	Subnational	both	30-69	30-69	1,011	938	Yes	Standard mercury	
330	Germany	1991-1992	MONICA, Erfurt	Community	urban	30-64	30-64	489	445	Yes	Standard mercury	

	Country	Data years	Survey/Study name/Citation	Level of representative-ness	Rural, urban, or both	Age range used in the global analysis		Sample size used in the global analysis		Information on diagnosis	Type of blood pressure measurement device*	Note
						Female	Male	Female	Male			
331	Germany	1993-1994	MONICA, Chemnitz	Community	urban	30-64	30-64	391	368	Yes	Standard mercury	
332	Germany	1993-1994	MONICA, Zwickau	Community	urban	30-64	30-64	166	126	Yes	Standard mercury	
333	Germany	1994-1995	MONICA, Augsburg	Community	both	30-64	30-64	1,776	1,711	Yes	Random-zero mercury	
334	Germany	1994-1998	EPIC Heidelberg	Community	urban	35-64	40-64	5,362	4,389	Yes	Digital oscillometric	
335	Germany	1994-1998	EPIC Potsdam	Community	urban	35-64	40-64	15,210	9,509	Yes	Digital oscillometric	
336	Germany	1997-1999	German National Health Interview and Examination Survey (GNHIES98)	National	both	30-79	30-79	2,984	2,791	Yes	Standard mercury	
337	Germany	1997-2001	Study of Health in Pomerania (SHIP-0) baseline study	Subnational	both	30-79	30-79	1,852	1,811	No	Digital oscillometric	
338	Germany	1999-2001	KORA S4 Study: Kooperative Research in the Region of Augsburg Survey 4	Community	both	30-75	30-75	1,981	1,927	No	Random-zero mercury	
339	Germany	2000-2002	Epidemiological study of the chances of prevention, early recognition and optimal treatment of chronic diseases in an elderly population (ESTHER)	Subnational	both	50-75	50-75	5,301	4,311	Yes	Unknown	
340	Germany	2000-2003	Heinz Nixdorf Recall Study	Subnational	urban	45-75	45-75	2,384	2,343	Yes	Digital oscillometric	6
341	Germany	2005-2008	Heinz Nixdorf Recall Study	Subnational	both	50-79	50-79	2,024	1,978	Yes	Digital oscillometric	6
342	Germany	2006-2008	KORA F4 Study: Kooperative Research in the Region of Augsburg Follow-Up of Survey 4	Community	both	31-79	32-79	1,560	1,454	No	Random-zero mercury	
343	Germany	2008-2011	Epidemiological study of the chances of prevention, early recognition and optimal treatment of chronic diseases in an elderly population (ESTHER)	Subnational	both	58-79	58-79	985	879	Yes	Aneroid	
344	Germany	2008-2011	German Health Interview and Examination Survey for adults 2008-11 (DEGS1)	National	both	30-79	30-79	3,108	2,838	Yes	Digital oscillometric	
345	Germany	2008-2012	Study of Health in Pomerania, second cohort (SHIP-TREND)	Subnational	both	30-79	30-79	2,033	1,901	No	Digital oscillometric	
346	Germany	2011-2014	Heinz Nixdorf Recall Study	Subnational	both	56-79	56-79	1,428	1,379	Yes	Digital oscillometric	6
347	Ghana	2001	Cappuccio et al., Hypertension 2004; 43: 1017-22	Community	both	40-75	40-75	628	385	No	Unknown	
348	Ghana	2003	Women's Health Study of Accra (WHSA-I)	Community	urban	30-79		887		Yes	Aneroid	
349	Ghana	2006	STEPS	Community	urban	30-69	30-69	1,385	715	Yes	Digital oscillometric	
350	Ghana	2007-2008	SAGE	National	both	50-79	50-79	1,812	2,009	Yes	Digital oscillometric	
351	Ghana	2008-2010	Women's Health Study of Accra (WHSA-II)	Community	urban	30-79		1,990		Yes	Aneroid	
352	Ghana	2012	Williams et al., Diabetes Metab Syndr 2013; 7: 123-8	Subnational	rural	35-79	35-79	253	143	Yes	Standard mercury	
353	Ghana	2013	Williams et al., J Hypertens 2014; 3: 147	Community	rural	35-79	35-79	241	138	Yes	Aneroid	
354	Ghana	2012-2014	Research on Obesity and Diabetes among African Migrants (RODAM), control group	Subnational	urban	30-79	30-79	898	359	Yes	Digital oscillometric	
355	Ghana	2012-2014	Research on Obesity and Diabetes among African Migrants (RODAM), control group	Subnational	rural	30-79	30-79	554	349	Yes	Digital oscillometric	
356	Ghana	2014	Demographic and Health Survey Ghana 2014	National	both	30-49	30-59	2,134	2,282	Yes	Digital oscillometric	
357	Greece	1991-1999	EPIC	National	both	30-79	30-79	15,478	10,835	Yes	Standard mercury	
358	Greece	1997	The Didima Study	Community	rural	30-79	30-79	312	232	Yes	Standard mercury	
359	Greece	2006	Paliouri Study	Community	rural	65-79	65-79	58	77	Yes	Unknown	
360	Greece	2013-2015	Hellenic National Nutrition and Health Survey (HNNHS)	National	both	30-79	30-79	425	272	Yes	Digital oscillometric	
361	Greece	2013-2016	National Survey of Morbidity and Risk Factors (EMENO)	National	both	30-79	30-79	2,248	1,637	Yes	Digital oscillometric	
362	Greece	2016	SKG-Elderly	Community	urban	60-79	60-79	51	36	Yes	Digital oscillometric	
363	Greece	2018	STEPS-Thessaloniki	Community	both	60-79	60-79	108	242	Yes	Digital oscillometric	
364	Greenland	2005-2010	Population Health Survey in Greenland	National	both	30-79	30-79	1,336	1,066	Yes	Digital oscillometric	
365	Grenada	2011	STEPS	National	both	30-64	30-64	547	365	Yes	Digital oscillometric	
366	Guatemala	2001-2002	CAMDI	Community	urban	30-79	30-79	477	233	Yes	Unknown	
367	Guatemala	2003-2005	The Institute of Nutrition of Central America and Panama Nutrition Supplementation Trial Cohort	Community	both	30-41	30-41	191	152	Yes	Digital oscillometric	
368	Guatemala	2015	STEPS	Subnational	urban	30-79	30-79	1,142	303	Yes	Digital oscillometric	
369	Guinea	2009	STEPS	Subnational	both	30-64	30-64	643	697	Yes	Digital oscillometric	
370	Guyana	2016	STEPS	National	both	30-69	30-69	1,129	788	Yes	Digital oscillometric	
371	Haiti	2015-2016	Haiti Health Study (Thomonde)	Community	rural	30-65	30-65	359	213	Yes	Digital oscillometric	
372	Haiti	2016-2017	Demographic and Health Survey Haiti 2016-2017	National	both	35-64	35-64	2,482	2,083	Yes	Unknown	
373	Honduras	2003-2004	CAMDI	Community	urban	30-79	30-79	547	303	Yes	Unknown	
374	Hungary	2003	The European Male Ageing Study	Community	both		40-79		420	Yes	Digital oscillometric	
375	Hungary	2008	The European Male Ageing Study	Community	both		40-79		214	Yes	Digital oscillometric	
376	Iceland	1993-1994	MONICA, Arnes County	Community	rural	30-64	30-64	427	379	Yes	Standard mercury	
377	Iceland	1993-1994	MONICA, Reykjavik	Subnational	urban	30-64	30-64	409	427	Yes	Standard mercury	
378	Iceland	2002-2006	AGES-Reykjavik Study	Subnational	urban	66-79	66-79	2,140	1,591	Yes	Standard mercury	
379	Iceland	2005-2011	Risk Evaluation For INfarct Estimates (REFINE)	Subnational	urban	30-73	30-73	3,206	3,062	Yes	Digital oscillometric	
380	Iceland	2007-2011	AGES-Reykjavik Study - follow up visit	Subnational	urban	71-79	71-79	930	649	Yes	Standard mercury	
381	Iceland	2010-2012	Risk Evaluation For INfarct Estimates (REFINE) follow-up visit (REFINELO)	Subnational	urban	30-74	30-74	638	633	Yes	Digital oscillometric	
382	Iceland	2012-2013	Risk Evaluation For INfarct Estimates (REFINE) follow-up visit (REFLOCT)	Subnational	urban	55-73	55-73	547	502	Yes	Digital oscillometric	
383	India	1992-1994	Jaipur Heart Watch 1	Community	urban	30-79	30-79	641	875	Yes	Standard mercury	

	Country	Data years	Survey/Study name/Citation	Level of representative-ness	Rural, urban, or both	Age range used in the global analysis		Sample size used in the global analysis		Information on diagnosis	Type of blood pressure measurement device*	Note
						Female	Male	Female	Male			
384	India	1995	Malhotra et al., J Hum Hypertens 1999; 13: 467-72	Community	both	31-70	31-70	701	656	No	Unknown	
385	India	1995-1996	Epidemiology of blood pressure across cross-cultural populations of Visakhapatnam district, Andhra Pradesh, India	Community	rural	30-76	30-76	174	162	Yes	Standard mercury	
386	India	1996-1999	Chennai Urban Population Study	Community	urban	30-79	30-79	558	410	Yes	Standard mercury	
387	India	1999	Swami et al., Public Health 2002; 116: 45-9	Community	both	65-74	65-74	153	88	No	Unknown	
388	India	2000	Barucha and Kuruvilla, BMC Public Health 2003; 3: 1	Community	urban	30+	30+	1,146	947	No	Unknown	1
389	India	2001	Gupta et al., BMC Cardiovasc Disord 2009; 9: 28	Community	urban	30-39	30-39	311	133	No	Unknown	
390	India	1999-2002	New Delhi Birth Cohort	Community	urban	30-33	30-33	179	250	Yes	Digital oscillometric	
391	India	2002-2003	Blood pressure epidemiology in tribal, rural and urban communities of Orissa with special reference to physical and social parameters	Community	rural	30-79	30-79	126	134	Yes	Standard mercury	
392	India	2001-2004	Chennai Urban Rural Epidemiology Study	Community	urban	30-79	30-79	924	849	Yes	Standard mercury	
393	India	2003-2005	India STEPS, Trivandrum (semiurban)	Subnational	urban	30-69	30-69	889	883	Yes	Digital oscillometric	
394	India	2003-2005	India STEPS, Trivandrum	Subnational	urban	30-69	30-69	896	893	Yes	Digital oscillometric	
395	India	2003-2005	India STEPS, Trivandrum	Subnational	rural	30-69	30-69	923	872	Yes	Digital oscillometric	
396	India	2003-2005	India STEPS, Dibrugarh	Subnational	rural	30-69	30-69	938	1,053	Yes	Digital oscillometric	
397	India	2003-2005	India STEPS, Ballabgarh	Subnational	rural	30-69	30-69	973	883	Yes	Digital oscillometric	
398	India	2003-2005	India STEPS, Chennai	Subnational	rural	30-69	30-69	940	969	Yes	Digital oscillometric	
399	India	2003-2005	India STEPS, Delhi	Subnational	urban	30-69	30-69	907	852	Yes	Digital oscillometric	
400	India	2003-2005	India STEPS, Nagpur	Subnational	urban	30-69	30-69	886	866	Yes	Digital oscillometric	
401	India	2003-2005	India STEPS, Nagpur (semiurban)	Subnational	urban	30-69	30-69	989	846	Yes	Digital oscillometric	
402	India	2003-2005	India STEPS, Ballabgarh (semiurban)	Subnational	urban	30-69	30-69	905	891	Yes	Digital oscillometric	
403	India	2003-2005	India STEPS, Ballabgarh	Subnational	urban	30-69	30-69	936	899	Yes	Digital oscillometric	
404	India	2003-2005	India STEPS, Delhi (semiurban)	Subnational	urban	30-69	30-69	919	869	Yes	Digital oscillometric	
405	India	2002-2005	The Chennai Glaucoma Study	Subnational	urban	40-79	40-79	1,881	1,489	No	Standard mercury	
406	India	2003-2005	India STEPS, Nagpur	Subnational	rural	30-69	30-69	863	866	Yes	Digital oscillometric	
407	India	2003-2005	India STEPS, Chennai	Subnational	urban	30-69	30-69	891	893	Yes	Digital oscillometric	
408	India	2003-2005	India STEPS, Dibrugarh (semiurban)	Subnational	urban	30-69	30-69	1,026	909	Yes	Digital oscillometric	
409	India	2003-2005	India STEPS, Dibrugarh	Subnational	urban	30-69	30-69	886	891	Yes	Digital oscillometric	
410	India	2003-2005	India STEPS, Chennai (semiurban)	Subnational	urban	30-69	30-69	924	866	Yes	Digital oscillometric	
411	India	2005-2007	Prevalence of cardiovascular risk factors in rural Tamil Nadu	Community	rural	30-64	30-64	4,474	3,902	Yes	Digital oscillometric	
412	India	2005-2006	Risk factor profile for chronic non-communicable diseases: Results of a community-based study in Kerala, India	Community	both	30-64	30-64	2,106	1,999	Yes	Digital oscillometric	
413	India	2005-2006	National Nutrition Monitoring Bureau Rural Survey	National	rural	30-79	30-79	9,996	8,982	Yes	Standard mercury	
414	India	2006-2008	Central India Eye and Medical Study	Community	rural	30-79	30-79	2,499	2,133	Yes	Digital oscillometric	
415	India	2007-2008	Integrated Disease Surveillance Project Non-communicable Disease Risk Factors Survey	Subnational	both	30-65	30-65	2,087	1,378	Yes	Digital oscillometric	
416	India	2007-2008	Integrated Disease Surveillance Project Non-communicable Disease Risk Factors Survey	Subnational	both	30-65	30-65	2,050	1,502	Yes	Digital oscillometric	
417	India	2007-2008	Integrated Disease Surveillance Project Non-communicable Disease Risk Factors Survey	Subnational	both	30-65	30-65	2,004	1,837	Yes	Digital oscillometric	
418	India	2007-2008	Integrated Disease Surveillance Project Non-communicable Disease Risk Factors Survey	Subnational	both	30-65	30-65	1,226	1,449	Yes	Digital oscillometric	
419	India	2006-2009	New Delhi Birth Cohort	Community	urban	33-38	33-38	442	644	Yes	Digital oscillometric	
420	India	2007-2008	Integrated Disease Surveillance Project Non-communicable Disease Risk Factors Survey	Subnational	both	30-65	30-65	2,326	1,921	Yes	Digital oscillometric	
421	India	2007-2008	Integrated Disease Surveillance Project Non-communicable Disease Risk Factors Survey	Subnational	both	30-65	30-65	2,050	2,147	Yes	Digital oscillometric	
422	India	2007-2009	Prevalence of NCD risk factor in people above 15 year in Rural area Nagpur using WHO STEP approach	Community	rural	30-79	30-79	1,188	1,261	No	Digital oscillometric	
423	India	2007-2008	Integrated Disease Surveillance Project Non-communicable Disease Risk Factors Survey	Subnational	both	30-65	30-65	2,390	1,263	Yes	Digital oscillometric	
424	India	2007-2008	SAGE	National	both	50-79	50-79	3,045	3,093	Yes	Digital oscillometric	
425	India	2008-2010	ICMR India Diabetes Study, Phase I	National	both	30-79	30-79	4,949	5,091	Yes	Digital oscillometric	
426	India	2009-2010	Baseline Survey for the assessment of prevalence of risk factors of NCDs in Gandhinagar District	Community	rural	30-64	30-64	551	571	Yes	Digital oscillometric	
427	India	2009-2010	Baseline Survey for the assessment of prevalence of risk factors of NCDs in Gandhinagar District	Community	urban	30-64	30-64	623	608	Yes	Digital oscillometric	
428	India	2010	Kerala 2010 follow-up	Community	rural	30-70	30-70	205	173	Yes	Digital oscillometric	
429	India	2011-2012	National Nutrition Monitoring Bureau Rural Survey	National	rural	30-79	30-79	20,027	16,069	Yes	Digital oscillometric	
430	India	2012-2013	ICMR India Diabetes Study, Phase II	National	both	30-79	30-79	8,320	6,342	Yes	Digital oscillometric	
431	India	2012-2015	ICMR India Diabetes Study, North East Phase	Subnational	both	30-79	30-79	9,479	7,951	Yes	Digital oscillometric	
432	India	2015-2016	Demographic and Health Survey India 2015-2016	National	both	30-49	30-54	326,125	58,601	Yes	Digital oscillometric	
433	India	2015-2016	Diet and nutritional status of urban population and prevalence of hypertension	National	urban	30-79	30-79	41,951	32,212	Yes	Digital oscillometric	
434	Indonesia	2001	STEPS; Ng et al., Bull World Health Organ 2006; 84: 305-13	Community	both	25-74	25-74	1,234	1,261	No	Unknown	1

	Country	Data years	Survey/Study name/Citation	Level of representative-ness	Rural, urban, or both	Age range used in the global analysis		Sample size used in the global analysis		Information on diagnosis	Type of blood pressure measurement device*	Note
						Female	Male	Female	Male			
435	Indonesia	2000-2001	Indonesian Family Life Surveys	National	both	30-79	30-79	7,483	6,740	No	Digital oscillometric	
436	Indonesia	2003	A genetic-ecological study of the risk factors for lifestyle-related diseases in Oceanian populations, Study B	Community	rural	30-79	30-79	82	55	No	Digital oscillometric	
437	Indonesia	2003	A genetic-ecological study of the risk factors for lifestyle-related diseases in Oceanian populations, Study A	Community	rural	30-79	30-79	62	76	No	Digital oscillometric	
438	Indonesia	2003	STEPS; Tesfaye et al., J Hum Hypertens 2007; 21: 28-37	Subnational	both	25-64	25-64	960	984	No	Unknown	1
439	Indonesia	2007-2008	Indonesian Family Life Surveys	National	both	30-79	30-79	5,485	4,886	Yes	Digital oscillometric	
440	Indonesia	2014-2015	Indonesian Family Life Surveys	National	both	30-79	30-79	10,918	10,131	Yes	Digital oscillometric	
441	Indonesia	2018	Indonesian Basic Health Survey 2018	National	both	30-79	30-79	11,534	6,973	Yes	Digital oscillometric	
442	Iran	1999-2001	Tehran Lipid and Glucose Study	Community	both	30-79	30-79	4,423	3,391	Yes	Standard mercury	
443	Iran	2001	Isfahan Healthy Heart Program (IHHP), Arak	Community	urban	30-79	30-79	1,481	1,398	Yes	Standard mercury	
444	Iran	2001	Isfahan Healthy Heart Program (IHHP), Arak	Community	rural	30-79	30-79	735	675	Yes	Standard mercury	
445	Iran	2001	Isfahan Healthy Heart Program (IHHP), Najaf Abad	Community	urban	30-79	30-79	395	387	Yes	Standard mercury	
446	Iran	2001	Isfahan Healthy Heart Program (IHHP), Najaf Abad	Community	rural	30-79	30-79	297	266	Yes	Standard mercury	
447	Iran	2001	Isfahan Healthy Heart Program (IHHP), Isfahan	Community	urban	30-79	30-79	1,328	1,168	Yes	Standard mercury	
448	Iran	2001	Isfahan Healthy Heart Program (IHHP), Isfahan	Community	rural	30-79	30-79	155	149	Yes	Standard mercury	
449	Iran	2002-2005	Tehran Lipid and Glucose Study	Community	both	30-79	30-79	2,082	1,618	Yes	Standard mercury	
450	Iran	2003-2004	The Persian Gulf Healthy Heart Study	Subnational	urban	30-75	30-75	1,621	1,412	Yes	Standard mercury	
451	Iran	2005	Provincial Non-Communicable Disease Surveillance Survey 2005	National	both	30-64	30-64	27,614	28,228	Yes	Digital oscillometric	
452	Iran	2006	Provincial Non-Communicable Disease Surveillance Survey 2006	National	both	30-65	30-65	10,377	10,443	Yes	Digital oscillometric	
453	Iran	2004-2008	Golestan Cohort Study Main Phase	Community	urban	40-75	40-75	6,098	3,933	Yes	Standard mercury	
454	Iran	2004-2008	Golestan Cohort Study Main Phase	Subnational	rural	40-75	40-75	22,681	17,281	Yes	Standard mercury	
455	Iran	2005-2008	Tehran Lipid and Glucose Study	Community	both	30-79	30-79	2,320	1,796	Yes	Standard mercury	
456	Iran	2007	Provincial Non-Communicable Disease Surveillance Survey 2007	National	both	30-64	30-64	10,207	10,244	Yes	Digital oscillometric	
457	Iran	2007	National Non-Communicable Disease Surveillance Survey 2007	National	both	30-64	30-64	1,626	1,633	Yes	Digital oscillometric	
458	Iran	2007	Isfahan Healthy Heart Program (IHHP), Arak	Community	urban	30-79	30-79	879	917	Yes	Standard mercury	
459	Iran	2007	Isfahan Healthy Heart Program (IHHP), Arak	Community	rural	30-79	30-79	739	620	Yes	Standard mercury	
460	Iran	2007	Isfahan Healthy Heart Program (IHHP), Najaf Abad	Community	urban	30-79	30-79	330	325	Yes	Standard mercury	
461	Iran	2007	Isfahan Healthy Heart Program (IHHP), Najaf Abad	Community	rural	30-79	30-79	162	155	Yes	Standard mercury	
462	Iran	2007	Isfahan Healthy Heart Program (IHHP), Isfahan	Community	urban	30-79	30-79	933	869	Yes	Standard mercury	
463	Iran	2007	Isfahan Healthy Heart Program (IHHP), Isfahan	Community	rural	30-79	30-79	102	93	Yes	Standard mercury	
464	Iran	2008	Provincial Non-Communicable Disease Surveillance Survey 2008	National	both	30-64	30-64	10,014	10,231	Yes	Digital oscillometric	
465	Iran	2009	Provincial Non-Communicable Disease Surveillance Survey 2009	National	both	30-64	30-64	10,137	10,165	Yes	Digital oscillometric	
466	Iran	2008-2011	Tehran Lipid and Glucose Study	Community	urban	30-79	30-79	4,553	3,478	Yes	Standard mercury	
467	Iran	2009-2010	The Persian Gulf Healthy Heart Study	Subnational	urban	31-79	31-79	1,014	834	Yes	Standard mercury	
468	Iran	2010-2011	The Yazd Eye Study	Subnational	both	40-79	40-79	878	735	Yes	Standard mercury	
469	Iran	2010-2012	Golestan Cohort Study Second Phase	Subnational	rural	43-79	43-79	4,913	4,308	Yes	Standard mercury	
470	Iran	2010-2012	Golestan Cohort Study Second Phase	Community	urban	43-79	43-79	1,061	1,086	Yes	Standard mercury	
471	Iran	2011	Provincial Non-Communicable Disease Surveillance Survey 2011	National	both	30-69	30-69	3,085	1,786	Yes	Digital oscillometric	
472	Iran	2011-2012	Amol county study	Community	rural	30-79	30-79	767	1,282	Yes	Standard mercury	
473	Iran	2011-2012	Amol county study	Community	urban	30-79	30-79	1,162	1,136	Yes	Standard mercury	
474	Iran	2012-2013	Tehran City	Community	urban	30-79	30-79	410	307	Yes	Standard mercury	
475	Iran	2011-2014	Tehran Lipid and Glucose Study	Community	both	30-79	30-79	2,655	2,046	Yes	Standard mercury	
476	Iran	2012-2014	Pars Cohort Study	Community	rural	40-79	40-79	4,953	4,216	Yes	Standard mercury	
477	Iran	2012-2013	Zahedan City	Community	urban	30-79	30-79	731	792	Yes	Standard mercury	
478	Iran	2013-2014	Gilan Eye Study	Subnational	both	50-79	50-79	1,359	955	Yes	Standard mercury	
479	Iran	2013-2014	Bushehr Elderly Health Program (BEH)	Community	urban	60-79	60-79	1,366	1,262	Yes	Standard mercury	
480	Iran	2014-2016	The PERSIAN Kermanshah Cohort Study	Community	urban	35-69	35-69	2,975	2,947	Yes	Standard mercury	
481	Iran	2014-2016	The PERSIAN Kharameh Cohort Study	Community	rural	35-70	35-70	3,861	2,914	Yes	Standard mercury	
482	Iran	2014-2016	The PERSIAN Guilan Cohort Study	Community	rural	35-70	35-70	3,261	2,646	Yes	Standard mercury	
483	Iran	2014-2016	The PERSIAN Guilan Cohort Study	Community	urban	35-69	35-70	2,350	2,236	Yes	Standard mercury	
484	Iran	2014-2016	The PERSIAN Kermanshah Cohort Study	Community	rural	35-69	35-69	2,198	1,809	Yes	Standard mercury	
485	Iran	2014-2016	The PERSIAN Tabriz Cohort Study	Community	urban	35-70	35-70	5,592	4,670	Yes	Standard mercury	
486	Iran	2014-2016	The PERSIAN Tabriz Cohort Study	Community	rural	35-70	35-70	2,547	1,974	Yes	Standard mercury	
487	Iran	2014-2016	The PERSIAN Fasa Cohort Study	Community	rural	35-70	35-70	4,579	3,747	Yes	Standard mercury	
488	Iran	2014-2016	The PERSIAN Fasa Cohort Study	Community	urban	35-70	35-70	812	720	Yes	Standard mercury	
489	Iran	2014-2016	The PERSIAN Kharameh Cohort Study	Community	urban	35-70	35-70	2,001	1,795	Yes	Standard mercury	

	Country	Data years	Survey/Study name/Citation	Level of representative-ness	Rural, urban, or both	Age range used in the global analysis		Sample size used in the global analysis		Information on diagnosis	Type of blood pressure measurement device*	Note
						Female	Male	Female	Male			
490	Iran	2014-2017	Tehran Lipid and Glucose Study	Community	both	30-79	30-79	2,542	1,943	Yes	Standard mercury	
491	Iran	2016	Non-Communicable Disease Surveillance Survey 2016	National	both	30-79	30-79	11,753	10,887	Yes	Digital oscillometric	
492	Iran	2015-2017	The PERSIAN Yazd Cohort Study	Community	urban	30-70	30-70	4,750	4,822	Yes	Standard mercury	
493	Iran	2015-2017	The PERSIAN Rafsanjan Cohort Study	Community	rural	35-69	35-69	1,024	1,550	Yes	Standard mercury	
494	Iran	2015-2017	The PERSIAN Rafsanjan Cohort Study	Community	urban	35-70	35-70	4,254	3,567	Yes	Standard mercury	
495	Iran	2015-2017	The PERSIAN Mazandaran Cohort Study	Community	rural	35-70	35-70	1,608	936	Yes	Standard mercury	
496	Iran	2015-2017	The PERSIAN Mazandaran Cohort Study	Community	urban	35-70	35-70	4,421	3,179	Yes	Standard mercury	
497	Iran	2016-2018	The PERSIAN Urmia Cohort Study	Community	rural	35-70	35-70	2,326	1,776	Yes	Standard mercury	
498	Iran	2016-2018	The PERSIAN Urmia Cohort Study	Community	urban	35-70	35-70	480	412	Yes	Standard mercury	
499	Iran	2015-2018	The PERSIAN Zahedan Cohort Study	Community	urban	35-70	35-70	5,987	3,893	Yes	Standard mercury	
500	Iran	2016-2018	The PERSIAN BandarKong Cohort Study	Community	rural	35-69	35-69	366	232	Yes	Standard mercury	
501	Iran	2016-2018	The PERSIAN BandarKong Cohort Study	Community	urban	35-70	35-70	1,889	1,471	Yes	Standard mercury	
502	Iran	2016-2018	The PERSIAN Ahvaz Cohort Study	Community	rural	35-70	35-70	2,281	1,479	Yes	Standard mercury	
503	Iran	2016-2018	The PERSIAN Ahvaz Cohort Study	Community	urban	35-70	35-70	3,554	2,512	Yes	Standard mercury	
504	Iran	2016-2019	The PERSIAN Shahrekord Cohort Study	Community	rural	35-70	35-70	985	771	Yes	Standard mercury	
505	Iran	2016-2019	The PERSIAN Shahrekord Cohort Study	Community	urban	35-70	35-70	3,421	3,402	Yes	Standard mercury	
506	Iran	2016-2019	The PERSIAN Ardabil Cohort Study	Community	urban	35-70	35-70	7,921	6,650	Yes	Standard mercury	
507	Iran	2018-2019	Prevalence of risk factors for cardiovascular disease among a rural population in eastern Iran	Subnational	rural	30-69	30-69	126	118	No	Digital oscillometric	
508	Iran	2017-2018	The PERSIAN Kavar Cohort Study	Community	urban	35-70	35-70	2,539	2,418	Yes	Standard mercury	
509	Iran	2017-2018	PERSIAN Elderly Component-Iranian Longitudinal Study on Ageing	Subnational	both	50-79	50-79	3,489	3,038	Yes	Standard mercury	
510	Iran	2016-2019	The Khuzestan comprehensive health study: A platform for NCDs, blood borne and mental diseases research	Subnational	both	30-65	30-65	15,657	8,929	Yes	Standard mercury	
511	Iraq	2015	STEPS	National	both	30-79	30-79	1,560	974	Yes	Standard mercury	
512	Ireland	2006-2007	Survey of Lifestyle, Attitudes and Nutrition in Ireland	National	both	45-79	45-79	679	526	Yes	Digital oscillometric	
513	Ireland	2009-2011	The Irish Longitudinal Study on Ageing	National	both	50-79	50-79	2,977	2,542	Yes	Digital oscillometric	
514	Israel	1990-1991	The Jerusalem Longitudinal Cohort Study	Community	urban	69-70	69-70	204	248	Yes	Standard mercury	
515	Israel	1997-1998	The Jerusalem Longitudinal Cohort Study	Community	urban	76-77	76-77	429	434	Yes	Standard mercury	
516	Israel	1999-2005	The Israel Glucose Intolerance, Obesity and Hypertension Study	National	urban	58-79	58-79	405	424	Yes	Standard mercury	
517	Israel	2002-2007	Hadera District Study	Subnational	urban	30-78	30-78	349	367	Yes	Standard mercury	
518	Israel	2005-2006	Mabat Zahav National Health and Nutrition Survey	National	urban	65-79	65-79	671	595	Yes	Digital oscillometric	
519	Israel	2014-2016	Second National Health and Nutrition Survey, Adults Ages 18-64	National	both	30-64	30-64	896	798	Yes	Digital oscillometric	
520	Israel	2014-2015	Mabat Zahav Second National Health and Nutrition Survey ages in 65 and over	National	both	65-79	65-79	315	291	Yes	Digital oscillometric	
521	Italy	1989-1990	MONICA, Brianza	Subnational	urban	30-64	30-64	703	681	Yes	Standard mercury	
522	Italy	1990	Bruneck Study	Community	rural	40-79	40-79	450	469	Yes	Standard mercury	
523	Italy	1992-1993	Italian Longitudinal Study on Aging	National	both	65-79	65-79	1,237	1,352	Yes	Standard mercury	
524	Italy	1993-1994	MONICA, Brianza	Subnational	urban	30-64	30-64	749	706	Yes	Standard mercury	
525	Italy	1994	MONICA, Friuli	Subnational	both	30-64	30-64	790	781	Yes	Standard mercury	
526	Italy	1993-1996	Malattie cardiovascolari ATerosclerotiche Istituto Superiore di Sanità (MATISS)	Community	rural	30-77	30-77	788	771	Yes	Unknown	
527	Italy	1995	Bruneck Study	Community	rural	45-79	45-79	375	374	Yes	Standard mercury	
528	Italy	1995-1996	Friuli Studio Emostatico	Community	urban	45-64	45-64	198	198	No	Standard mercury	
529	Italy	1995-1996	Italian Longitudinal Study on Aging	National	both	68-79	68-79	634	745	Yes	Standard mercury	
530	Italy	1993-1998	EPIC Florence	Community	urban	30-69	30-69	9,339	3,248	Yes	Standard mercury	
531	Italy	1995-1999	PROgetto Veneto Anziani (PROVA)	Subnational	both	65-79	65-79	1,205	800	Yes	Standard mercury	
532	Italy	1998-1999	Progetto VIP	Community	both	30-74	30-74	538	538	Yes	Digital oscillometric	
533	Italy	1998-2002	Osservatorio Epidemiologico Cardiovascolare (OEC)	National	both	35-74	35-74	4,770	4,873	Yes	Unknown	
534	Italy	2000	Bruneck Study	Community	rural	50-79	50-79	309	295	Yes	Standard mercury	
535	Italy	2000-2001	Italian Longitudinal Study on Aging	National	both	73-79	73-79	344	361	Yes	Standard mercury	
536	Italy	2001-2003	The Study of Asti	Community	both	45-64	45-64	878	780	No	Standard mercury	
537	Italy	2000-2003	PROgetto Veneto Anziani (PROVA)	Subnational	both	67-79	67-79	761	446	Yes	Standard mercury	
538	Italy	2003	The European Male Ageing Study	Community	both		40-79		426	Yes	Digital oscillometric	
539	Italy	2002-2005	PROgetto Veneto Anziani (PROVA)	Subnational	both	70-79	70-79	573	326	No	Standard mercury	
540	Italy	2004	Menotti et al., J Hypertens 2009; 27: 266-74	Community	urban	20-79	20-79	2,055	1,761	No	Unknown	1
541	Italy	2004-2005	Italian Project on the Epidemiology of Alzheimer's Disease	National	both	65-79	65-79	954	1,020	Yes	Standard mercury	
542	Italy	2005	Bruneck Study	Community	rural	55-79	55-79	238	233	Yes	Standard mercury	
543	Italy	2004-2005	Vobarno study	Community	rural	55-74	55-74	113	99	No	Standard mercury	
544	Italy	2005-2007	Moli-family Study	Subnational	both	30-79	30-79	159	137	Yes	Digital oscillometric	

	Country	Data years	Survey/Study name/Citation	Level of representative-ness	Rural, urban, or both	Age range used in the global analysis		Sample size used in the global analysis		Information on diagnosis	Type of blood pressure measurement device*	Note
						Female	Male	Female	Male			
545	Italy	2005-2010	Moli-sani Study	Subnational	both	35-79	35-79	12,226	11,292	Yes	Digital oscillometric	
546	Italy	2008	The European Male Ageing Study	Community	both		40-79		236	Yes	Digital oscillometric	
547	Italy	2008-2009	Progetto VIP	Community	both	30-74	30-74	551	554	Yes	Digital oscillometric	
548	Italy	2008-2012	Osservatorio Epidemiologico Cardiovascolare/Health Examination Survey (OEC/HES)	National	both	35-79	35-79	4,311	4,349	Yes	Unknown	
549	Italy	2009-2010	Grosso et al., J Epidemiol 2014; 24: 327-33	Community	both	30-79	30-79	952	634	No	Unknown	
550	Italy	2010	Bruneck Study	Community	rural	60-79	60-79	184	180	Yes	Standard mercury	
551	Italy	2010-2012	CArdiovascular risk MEtabolic syndrome LIver and Autoimmunity diseases (CA.ME.LI.A)	Community	both	30-75	30-75	410	382	Yes	Aneroid	
552	Italy	2011-2012	Vobarno study	Community	rural	49-62	49-62	143	104	No	Standard mercury	
553	Italy	2015	Bruneck Study	Community	rural	65-79	65-79	122	134	Yes	Standard mercury	
554	Italy	2014-2016	Mediterranean healthy Eating, Aging and Lifestyles (MEAL) study	Subnational	urban	30-79	30-79	889	616	No	Aneroid	
555	Italy	2018-2019	Progetto VIP	Community	both	30-74	30-74	536	540	Yes	Digital oscillometric	
556	Italy	2017-2020	Moli-sani Study re-examination	Subnational	both	47-79	47-79	1,323	1,065	Yes	Digital oscillometric	
557	Jamaica	2000-2001	Jamaica Health and Lifestyle Survey	National	both	30-74	30-74	866	437	Yes	Standard mercury	
558	Jamaica	2007-2008	Jamaica Health and Lifestyle Survey	National	both	30-74	30-74	1,224	494	Yes	Standard mercury	
559	Jamaica	2012	Older Persons in Jamaica 2012	National	both	60-79	60-79	156	123	Yes	Aneroid	7
560	Japan	1987	National Nutrition Survey	National	both	30-79	30-79	5,040	3,375	No	Standard mercury	2
561	Japan	1988	National Nutrition Survey	National	both	30-79	30-79	4,810	3,420	No	Standard mercury	2
562	Japan	1989	National Nutrition Survey	National	both	30-79	30-79	4,425	3,063	No	Standard mercury	2
563	Japan	1990	National Nutrition Survey and National Cardiovascular Survey	National	both	30-79	30-79	4,737	3,434	Yes	Standard mercury	
564	Japan	1991	National Nutrition Survey	National	both	30-79	30-79	4,378	3,167	No	Standard mercury	
565	Japan	1990-1994	Japan Public Health Center-based prospective Study (JPHC Study), Cohort I	Subnational	both	40-59	40-59	14,486	8,746	Yes	Standard mercury	
566	Japan	1992	National Nutrition Survey	National	both	30-79	30-79	4,192	2,998	No	Standard mercury	
567	Japan	1993	National Nutrition Survey	National	both	30-79	30-79	3,912	2,707	No	Standard mercury	
568	Japan	1994	National Nutrition Survey	National	both	30-79	30-79	3,681	2,577	No	Standard mercury	
569	Japan	1993-1994	Japan Public Health Center-based prospective Study (JPHC Study), Cohort II	Subnational	both	40-69	40-69	16,226	8,567	Yes	Standard mercury	
570	Japan	1995	National Nutrition Survey	National	both	30-79	30-79	3,644	2,479	No	Standard mercury	
571	Japan	1996	National Nutrition Survey	National	both	30-79	30-79	3,545	2,394	No	Standard mercury	
572	Japan	1996-1997	INTERMAP, AitoTown	Community	rural	40-59	40-59	129	130	Yes	Random-zero mercury	
573	Japan	1997	National Nutrition Survey	National	both	30-79	30-79	3,490	2,380	No	Standard mercury	
574	Japan	1997-1998	INTERMAP, Sapporo	Community	urban	40-59	40-59	148	149	Yes	Random-zero mercury	
575	Japan	1997-1998	INTERMAP, Toyama	Community	urban	40-59	40-59	150	149	Yes	Random-zero mercury	
576	Japan	1998	National Nutrition Survey	National	both	30-79	30-79	3,716	2,584	No	Standard mercury	
577	Japan	1997-1998	INTERMAP, Wakayama	Community	urban	40-59	40-59	144	146	Yes	Random-zero mercury	
578	Japan	1998	Niigata Study	Community	urban	70-70	70-70	294	305	Yes	Standard mercury	
579	Japan	1999	National Nutrition Survey	National	both	30-79	30-79	3,059	2,054	No	Standard mercury	
580	Japan	1999	Niigata Study	Community	urban	71-71	71-71	216	243	Yes	Standard mercury	
581	Japan	2000	National Nutrition Survey and National Cardiovascular Survey	National	both	30-79	30-79	3,089	2,149	Yes	Standard mercury	
582	Japan	2000	Niigata Study	Community	urban	72-72	72-72	201	234	Yes	Standard mercury	
583	Japan	2001	National Nutrition Survey	National	both	30-79	30-79	3,106	1,990	No	Standard mercury	
584	Japan	2001	Niigata Study	Community	urban	73-73	73-73	197	235	Yes	Standard mercury	
585	Japan	2002	National Nutrition Survey	National	both	30-79	30-79	2,872	1,965	No	Standard mercury	
586	Japan	2002	Niigata Study	Community	urban	74-74	74-74	202	225	Yes	Standard mercury	
587	Japan	2002-2003	The Hisayama Study	Community	rural	40-79	40-79	1,671	1,313	Yes	Digital oscillometric	
588	Japan	2003	National Health and Nutrition Survey	National	both	30-79	30-79	2,876	1,895	No	Standard mercury	
589	Japan	2004	National Health and Nutrition Survey	National	both	30-79	30-79	2,167	1,418	No	Standard mercury	
590	Japan	2005	National Health and Nutrition Survey	National	both	30-79	30-79	2,069	1,415	No	Standard mercury	
591	Japan	2006	National Health and Nutrition Survey	National	both	30-79	30-79	2,348	1,592	No	Standard mercury	
592	Japan	2007	National Health and Nutrition Survey	National	both	30-79	30-79	2,232	1,512	No	Standard mercury	
593	Japan	2008	National Health and Nutrition Survey	National	both	30-79	30-79	2,371	1,651	No	Standard mercury	
594	Japan	2009	National Health and Nutrition Survey	National	both	30-79	30-79	2,286	1,576	No	Standard mercury	
595	Japan	2010	National Health and Nutrition Survey	National	both	30-79	30-79	2,109	1,466	Yes	Standard mercury	
596	Japan	2011	The Tokyo Health Service Association Database	Community	urban	30-79	30-79	35,187	64,206	No	Digital oscillometric	
597	Japan	2011	National Health and Nutrition Survey	National	both	30-79	30-79	1,905	1,342	No	Standard mercury	
598	Japan	2012	National Health and Nutrition Survey	National	both	30-79	30-79	7,704	5,303	No	Standard mercury	
599	Japan	2013	National Health and Nutrition Survey	National	both	30-79	30-79	1,788	1,259	No	Standard mercury	
600	Japan	2014-2015	Nagaoka Health Screening	Community	both	30-79	30-79	3,304	4,196	No	Digital oscillometric	
601	Japan	2014	National Health and Nutrition Survey	National	both	30-79	30-79	1,862	1,339	No	Standard mercury	

	Country	Data years	Survey/Study name/Citation	Level of representative-ness	Rural, urban, or both	Age range used in the global analysis		Sample size used in the global analysis		Information on diagnosis	Type of blood pressure measurement device*	Note
						Female	Male	Female	Male			
602	Japan	2012-2016	The Nagahama Study	Community	rural	35-69	35-69	5,488	2,330	Yes	Digital oscillometric	
603	Japan	2015	National Health and Nutrition Survey	National	both	30-79	30-79	1,825	1,224	No	Standard mercury	
604	Japan	2016	National Health and Nutrition Survey	National	both	30-79	30-79	6,086	4,236	No	Standard mercury	
605	Japan	2017	The Tokyo Health Service Association Database	Community	urban	30-79	30-79	32,491	50,562	No	Unknown	
606	Japan	2017	National Health and Nutrition Survey	National	both	30-79	30-79	1,564	1,079	No	Standard mercury	
607	Japan	2018	National Health and Nutrition Survey	National	both	30-79	30-79	1,551	1,097	No	Standard mercury	
608	Jordan	2004	Behavioural Risk Factor Surveillance Survey	National	rural	30-79	30-79	347	174	Yes	Standard mercury	
609	Jordan	2007	Behavioural Risk Factor Surveillance Survey	National	both	30-79	30-79	336	245	Yes	Standard mercury	
610	Jordan	2009	Metabolic abnormalities and vitamin D study	National	both	30-79	30-79	2,618	955	Yes	Standard mercury	
611	Jordan	2016-2017	National Cardiovascular Diseases and Diabetes Study (NCDDS)	National	both	30-79	30-79	2,164	1,014	Yes	Standard mercury	
612	Jordan	2019	STEPS	National	both	30-69	30-69	2,322	1,450	Yes	Digital oscillometric	
613	Kazakhstan	2015	STEPS, Shymkent	Subnational	both	30-79	30-79	674	315	Yes	Aneroid	
614	Kazakhstan	2015	STEPS, Almaty	Subnational	both	30-79	30-79	905	296	Yes	Aneroid	
615	Kazakhstan	2015-2016	STEPS, Aktobe	Subnational	both	30-69	30-69	942	260	Yes	Aneroid	
616	Kazakhstan	2019	A health status assessment of a population of Karaganda urban region	Community	urban	30-79	30-79	575	258	Yes	Aneroid	
617	Kenya	2015	STEPS	National	both	30-69	30-69	1,686	1,214	Yes	Digital oscillometric	
618	Kenya	2018	Assessing the gaps in healthcare and determining the feasibility for the setup of a social enterprise - Viwandani Lown Community Health Center, Kenya	Community	urban	30-59	30-73	85	99	Yes	Digital oscillometric	
619	Kiribati	2004	STEPS	National	both	30-64	30-64	652	518	Yes	Digital oscillometric	
620	Kiribati	2015-2016	STEPS	National	both	30-69	30-69	484	389	Yes	Digital oscillometric	
621	Kuwait	2006	STEPS	National	both	30-64	30-64	1,015	696	Yes	Standard mercury	
622	Kuwait	2008-2010	Gulf Cooperation Council World Health Survey	National	both	30-79	30-79	1,312	1,258	Yes	Standard mercury	
623	Kuwait	2011-2014	Kuwait Diabetes Epidemiology Program	National	urban	30-79	30-79	1,922	2,496	Yes	Digital oscillometric	
624	Kuwait	2014	STEPS	National	both	30-69	30-69	1,537	878	Yes	Standard mercury	
625	Kyrgyzstan	2012	Demographic and Health Survey Kyrgyzstan 2012	National	both	30-49	30-49	3,558	1,163	Yes	Digital oscillometric	
626	Kyrgyzstan	2013	STEPS	National	both	30-64	30-64	1,380	809	Yes	Digital oscillometric	
627	Lao PDR	2008	STEPS	Community	both	30-64	30-64	2,033	1,414	Yes	Digital oscillometric	
628	Lao PDR	2013	STEPS	National	both	30-64	30-64	1,080	759	Yes	Digital oscillometric	
629	Latvia	2008-2009	Cardiovascular risk factor study	National	both	25-74	25-74	2,409	1,358	Yes	Digital oscillometric	1
630	Lebanon	2008-2009	STEPS	National	both	30-79	30-79	982	823	Yes	Standard mercury	
631	Lebanon	2017	STEPS	National	both	30-69	30-69	871	633	Yes	Digital oscillometric	
632	Lesotho	2009-2010	Demographic and Health Survey Lesotho 2009-2010	National	both	30-49	30-59	1,536	1,317	Yes	Digital oscillometric	
633	Lesotho	2012	STEPS	National	both	30-64	30-64	1,195	621	Yes	Digital oscillometric	
634	Lesotho	2014	Demographic and Health Survey Lesotho 2014	National	both	30-49	30-59	1,317	1,257	Yes	Digital oscillometric	
635	Liberia	2011	STEPS	National	both	30-64	30-64	968	788	Yes	Digital oscillometric	
636	Libya	2009	STEPS	National	both	30-64	30-64	1,319	1,421	Yes	Digital oscillometric	
637	Lithuania	1992-1993	MONICA, Kaunas	Community	urban	35-64	35-64	627	609	Yes	Standard mercury	
638	Lithuania	1992-1993	Countrywide Integrated Noncommunicable Diseases Intervention Programme Survey	Subnational	rural	30-64	30-64	811	628	Yes	Standard mercury	
639	Lithuania	1998-1999	Countrywide Integrated Noncommunicable Diseases Intervention Programme Survey	Subnational	rural	30-64	30-64	959	769	Yes	Standard mercury	
640	Lithuania	2001-2002	MONICA4	Community	urban	35-64	35-64	767	615	Yes	Standard mercury	
641	Lithuania	2006-2007	Countrywide Integrated Noncommunicable Diseases Intervention Programme Survey	Subnational	rural	30-64	30-64	909	686	Yes	Standard mercury	
642	Lithuania	2006-2008	Health, Alcohol and Psychosocial factors In Eastern Europe (HAPIEE)	Community	urban	45-75	45-75	3,852	3,196	Yes	Digital oscillometric	
643	Luxembourg	2007-2009	Observation of cardiovascular risk factors in Luxembourg (ORISCAV-LUX)	National	both	30-69	30-69	619	590	Yes	Digital oscillometric	
644	Luxembourg	2013-2015	European Health Examination Survey	National	both	30-64	30-64	712	676	Yes	Digital oscillometric	
645	Luxembourg	2016-2018	Observation of cardiovascular risk factors in Luxembourg (ORISCAV-LUX) 2	National	both	30-79	30-79	740	637	Yes	Digital oscillometric	
646	Madagascar	2005	STEPS	Subnational	both	30-64	30-64	2,067	2,080	Yes	Digital oscillometric	
647	Malawi	2009	STEPS	National	both	30-64	30-64	1,922	920	Yes	Digital oscillometric	
648	Malawi	2013-2017	NCD Survey Malawi Epidemiology and Intervention Research Unit	Community	both	30-79	30-79	5,217	2,808	Yes	Digital oscillometric	
649	Malawi	2013-2017	NCD Survey Malawi Epidemiology and Intervention Research Unit	Community	both	30-79	30-79	4,914	3,486	Yes	Digital oscillometric	
650	Malawi	2017	STEPS	National	both	30-69	30-69	1,736	1,036	Yes	Digital oscillometric	
651	Malaysia	1996	National Health and Morbidity Survey (NHMS)	National	both	30-79	30-79	11,126	9,785	Yes	Digital oscillometric	
652	Malaysia	2004	Rampal et al., Public Health 2008; 122: 11-8	National	both	30-79	30-79	4,725	3,318	Yes	Standard mercury	
653	Malaysia	2006	National Health and Morbidity Survey (NHMS)	National	both	30-79	30-79	13,477	10,921	Yes	Digital oscillometric	
654	Malaysia	2011	National Health and Morbidity Survey (NHMS)	National	both	30-79	30-79	6,777	5,770	Yes	Digital oscillometric	
655	Malaysia	2015	National Health and Morbidity Survey (NHMS)	National	both	30-79	30-79	7,541	6,593	Yes	Digital oscillometric	
656	Maldives	2011	STEPS	Subnational	urban	30-64	30-64	684	346	Yes	Digital oscillometric	
657	Mali	2007	STEPS	Subnational	both	30-64	30-64	797	514	Yes	Aneroid	

	Country	Data years	Survey/Study name/Citation	Level of representative-ness	Rural, urban, or both	Age range used in the global analysis		Sample size used in the global analysis		Information on diagnosis	Type of blood pressure measurement device*	Note
						Female	Male	Female	Male			
658	Malta	2014-2016	SAHHTEK - The University of Malta Health and Wellbeing Study	National	both	30-70	30-70	860	725	Yes	Aneroid	
659	Marshall Islands	2002	STEPS	National	both	30-64	30-64	604	410	Yes	Digital oscillometric	
660	Marshall Islands	2017-2018	STEPS	National	both	30-79	30-79	1,070	966	Yes	Digital oscillometric	
661	Mauritius	1987	Mauritius Noncommunicable Disease Survey	National	both	30-74	30-74	2,242	1,978	Yes	Standard mercury	2
662	Mauritius	1992	Mauritius Noncommunicable Disease Survey	National	both	30-74	30-74	3,172	2,710	Yes	Standard mercury	
663	Mauritius	1998	Mauritius Noncommunicable Disease Survey	National	both	30-74	30-74	3,072	2,460	Yes	Standard mercury	
664	Mauritius	2009	Mauritius Noncommunicable Disease Survey	National	both	30-74	30-74	2,930	2,480	Yes	Digital oscillometric	
665	Mauritius	2015	Mauritius Noncommunicable Disease Survey	National	both	30-74	30-74	1,695	1,403	Yes	Digital oscillometric	
666	Mauritius	2015	Mauritius Noncommunicable Disease Survey (1998 Follow-up)	National	both	30-79	30-79	1,121	840	Yes	Digital oscillometric	
667	Mexico	1990-1992	Mexico City Diabetes Study	Community	urban	30-79	30-69	1,331	933	Yes	Random-zero mercury	
668	Mexico	1992	Yamamoto-Kimura et al., Arch Med Res 1998; 29: 341-9	Community	urban	30-69	30-69	63	44	No	Unknown	
669	Mexico	1992-1993	Encuesta Nacional de Enfermedades Cronicas	National	urban	30-69	30-69	5,402	3,791	Yes	Standard mercury	
670	Mexico	1993-1995	Mexico City Diabetes Study	Community	urban	34-79	34-69	1,027	702	Yes	Random-zero mercury	
671	Mexico	1995	Guerrero-Romero et al., Salud Publica Mex 1998; 40: 339-46	Community	rural	30-79	30-79	3,454	1,126	No	Unknown	
672	Mexico	1997-1999	Mexico City Diabetes Study	Community	urban	40-79	37-79	970	690	Yes	Random-zero mercury	
673	Mexico	1999-2000	The Survey on Health, Well-Being, and Aging in Latin America and the Caribbean (SABE)	Community	urban	60-79	60-79	499	316	Yes	Unknown	5
674	Mexico	2000	Encuesta Nacional de Salud	National	both	30-79	30-79	17,287	7,712	Yes	Standard mercury	
675	Mexico	2002	Encuesta Nacional Sobre Niveles de vida de los Hogares	National	both	30-79	30-79	5,348	4,268	Yes	Digital oscillometric	
676	Mexico	2005	Encuesta Nacional Sobre Niveles de vida de los Hogares	National	both	30-79	30-79	6,216	5,046	Yes	Digital oscillometric	
677	Mexico	2004-2005	CArdiovascular Risk factors Multiple Evaluation in Latin America (CARMELA)	Community	urban	25-64	25-64	889	833	Yes	Standard mercury	1
678	Mexico	2006	Encuesta Nacional de Salud Y Nutricion	National	both	30-79	30-79	14,962	10,017	Yes	Standard mercury	
679	Mexico	2007-2009	Mexico City Diabetes Study	Community	urban	51-79	51-79	689	445	Yes	Random-zero mercury	
680	Mexico	2009-2010	SAGE	National	both	50-79	50-79	1,110	710	Yes	Digital oscillometric	
681	Mexico	2009-2012	Encuesta Nacional Sobre Niveles de vida de los Hogares	National	both	30-79	30-79	6,850	5,192	Yes	Digital oscillometric	
682	Mexico	2011-2012	Encuesta Nacional de Salud Y Nutricion	National	both	30-79	30-79	5,353	3,532	Yes	Standard mercury	
683	Mexico	2012	The Mexican Health and Aging Study	National	both	50-79	50-79	1,035	733	Yes	Digital oscillometric	
684	Mexico	2016	Encuesta Nacional de Salud Y Nutricion	National	both	30-79	30-79	4,165	2,230	Yes	Unknown	
685	Mexico	2018-2019	Encuesta Nacional de Salud Y Nutricion	National	both	30-79	30-79	5,316	3,806	Yes	Unknown	
686	Micronesia (Federated States of)	2002	STEPS	Subnational	both	30-64	30-64	720	486	Yes	Digital oscillometric	
687	Micronesia (Federated States of)	2006	STEPS	Subnational	both	30-64	30-64	992	533	Yes	Digital oscillometric	
688	Micronesia (Federated States of)	2008	STEPS	Subnational	both	30-64	30-64	1,088	768	Yes	Digital oscillometric	
689	Micronesia (Federated States of)	2009	STEPS	Subnational	both	30-64	30-64	413	306	Yes	Digital oscillometric	
690	Micronesia (Federated States of)	2009	STEPS	Subnational	both	30-64	30-64	355	178	Yes	Digital oscillometric	
691	Micronesia (Federated States of)	2016	STEPS	Subnational	both	30-69	30-69	694	454	Yes	Digital oscillometric	
692	Moldova	2013	STEPS	National	both	30-69	30-69	2,341	1,410	Yes	Digital oscillometric	
693	Mongolia	1999	National Nutrition Survey; Suvd et al., Diabet Med 2002; 19: 502-8	National	both	35-64	35-64	1,317	907	No	Standard mercury	
694	Mongolia	2005	STEPS	National	both	30-64	30-64	1,207	1,153	Yes	Digital oscillometric	
695	Mongolia	2009	STEPS	National	both	30-64	30-64	2,228	1,548	Yes	Digital oscillometric	
696	Mongolia	2013	STEPS	National	both	30-64	30-64	1,935	1,582	Yes	Digital oscillometric	
697	Mongolia	2019	STEPS	National	both	30-69	30-69	2,787	2,276	Yes	Digital oscillometric	
698	Morocco	2017	STEPS	National	both	30-79	30-79	2,683	1,480	Yes	Digital oscillometric	
699	Mozambique	2005	STEPS	National	both	30-64	30-64	1,316	1,013	Yes	Digital oscillometric	
700	Mozambique	2014-2015	STEPS	National	both	30-64	30-64	982	668	Yes	Digital oscillometric	
701	Myanmar	2003-2004	STEPS	Subnational	both	30-74	30-74	2,251	1,830	Yes	Digital oscillometric	
702	Myanmar	2009	STEPS	National	both	30-64	30-64	3,460	2,126	Yes	Digital oscillometric	
703	Myanmar	2014	National survey of diabetes mellitus and risk factors for non-communicable diseases in Myanmar	National	both	30-64	30-64	4,957	2,635	Yes	Digital oscillometric	
704	Myanmar	2013-2014	STEPS 2013-2014 Yangon	Subnational	both	30-74	30-74	673	685	Yes	Digital oscillometric	
705	Namibia	2005	STEPS	National	both	30-64	30-64	1,463	1,115	No	Digital oscillometric	
706	Namibia	2013	Demographic and Health Survey Namibia 2013	National	both	30-64	30-64	2,023	1,526	Yes	Digital oscillometric	

	Country	Data years	Survey/Study name/Citation	Level of representative-ness	Rural, urban, or both	Age range used in the global analysis		Sample size used in the global analysis		Information on diagnosis	Type of blood pressure measurement device*	Note
						Female	Male	Female	Male			
707	Nauru	1987	Trends in the prevalence and incidence of non-insulin-dependent diabetes mellitus and impaired glucose tolerance	National	both	30-79	30-79	436	368	No	Standard mercury	2
708	Nauru	1994	Trends in the prevalence and incidence of non-insulin-dependent diabetes mellitus and impaired glucose tolerance	National	both	30-79	30-79	571	510	Yes	Standard mercury	
709	Nauru	2004	STEPS	National	both	30-64	30-64	742	679	Yes	Digital oscillometric	
710	Nauru	2006	STEPS	National	both	30-65	30-65	156	169	Yes	Digital oscillometric	
711	Nauru	2015	STEPS	National	rural	30-69	30-69	367	309	Yes	Digital oscillometric	
712	Nepal	2005	Noncommunicable Disease Risk Factors Survey; STEPS	Subnational	both	30-64	30-64	2,690	2,534	Yes	Digital oscillometric	
713	Nepal	2007-2008	Noncommunicable Disease Risk Factors Survey; STEPS	National	both	30-64	30-64	1,577	1,214	Yes	Digital oscillometric	
714	Nepal	2006-2011	Early detection and management of Kidney disease, Hypertension, Diabetes and Cardiovascular disease (KHDC Nepal), Tarahara	Community	rural	30-79	30-79	1,668	888	Yes	Standard mercury	
715	Nepal	2006-2011	Early detection and management of Kidney disease, Hypertension, Diabetes and Cardiovascular disease (KHDC Nepal), Damak	Community	urban	30-79	30-79	1,188	830	Yes	Standard mercury	
716	Nepal	2006-2011	Early detection and management of Kidney disease, Hypertension, Diabetes and Cardiovascular disease (KHDC Nepal), Dharan	Community	urban	30-79	30-79	3,980	2,664	Yes	Standard mercury	
717	Nepal	2012-2013	STEPS	National	both	30-69	30-69	2,108	1,040	Yes	Digital oscillometric	
718	Nepal	2015	Community based intervention for prevention and control of non-communicable diseases risk factors (CIPCON) baseline survey, Dhankuta	Subnational	rural	30-69	30-69	629	483	Yes	Digital oscillometric	
719	Nepal	2015	Community based intervention for prevention and control of non-communicable diseases risk factors (CIPCON) baseline survey, Ilam	Subnational	rural	30-69	30-69	569	461	Yes	Digital oscillometric	
720	Nepal	2016	Demographic and Health Survey Nepal 2016	National	both	30-49	30-49	2,866	1,928	Yes	Digital oscillometric	
721	Nepal	2016-2018	The Population Based Prevalence of Selected Non-Communicable Diseases In Nepal	National	both	30-79	30-79	6,266	4,226	No	Digital oscillometric	
722	Nepal	2019	STEPS	National	both	30-69	30-69	2,562	1,548	Yes	Digital oscillometric	
723	Netherlands	1990	Zutphen Elderly Study	Community	urban		69-79		446	Yes	Random-zero mercury	
724	Netherlands	1989-1993	The Rotterdam Study, first subcohort	Community	urban	55-79	55-79	3,370	2,493	No	Random-zero mercury	
725	Netherlands	1993-1995	The Rotterdam Study, first subcohort	Community	urban	56-79	56-79	2,553	1,957	No	Random-zero mercury	
726	Netherlands	1993-1997	EPIC Bilthoven	Community	urban	30-59	30-59	8,728	7,171	Yes	Random-zero mercury	
727	Netherlands	1993-1997	EPIC Utrecht	Community	both	49-70		16,852		Yes	Digital oscillometric	
728	Netherlands	1995-1996	The Longitudinal Aging Study Amsterdam (LASA)	Subnational	both	65-79	65-79	516	485	Yes	Digital oscillometric	8
729	Netherlands	1997-1999	The Rotterdam Study, first subcohort	Community	urban	61-79	61-79	1,822	1,406	No	Random-zero mercury	
730	Netherlands	1998-1999	The Longitudinal Aging Study Amsterdam (LASA)	Subnational	both	61-79	61-79	497	415	Yes	Digital oscillometric	8
731	Netherlands	2000	MPCDRF; Schelleman et al., J Hum Hypertens 2004; 18: 317-24	Community	urban	30-59	30-59	5,816	5,004	No	Unknown	
732	Netherlands	1998-2001	Regenboog Project	National	both	30-79	30-79	2,111	2,235	Yes	Digital oscillometric	
733	Netherlands	2000-2001	The Rotterdam Study, second subcohort	Community	urban	55-79	55-79	1,352	1,151	No	Random-zero mercury	
734	Netherlands	2001-2002	The Longitudinal Aging Study Amsterdam (LASA)	Subnational	both	64-79	64-79	493	433	Yes	Digital oscillometric	8
735	Netherlands	2001-2003	Surinamese in the Netherlands: Study on Ethnicity and Health (SUNSET)	Community	urban	35-60	35-60	257	250	Yes	Digital oscillometric	
736	Netherlands	2002	Utrecht Health Project; Scheltens et al., J Hum Hypertens 2007; 21: 99-106	Community	urban	30-59	30-59	1,763	1,592	No	Unknown	
737	Netherlands	2002-2003	The Longitudinal Aging Study Amsterdam (LASA)	Subnational	both	54-65	54-65	479	430	Yes	Digital oscillometric	8
738	Netherlands	2002-2004	The Rotterdam Study, first subcohort	Community	urban	65-79	65-79	1,342	1,010	No	Random-zero mercury	
739	Netherlands	2004-2005	The Rotterdam Study, second subcohort	Community	urban	58-79	58-79	1,143	909	No	Random-zero mercury	
740	Netherlands	2005-2006	The Longitudinal Aging Study Amsterdam (LASA)	Subnational	both	57-79	57-79	775	686	Yes	Digital oscillometric	8
741	Netherlands	2006-2008	The Rotterdam Study, third subcohort	Community	urban	45-79	45-79	2,014	1,544	No	Digital oscillometric	
742	Netherlands	2008-2009	The Longitudinal Aging Study Amsterdam (LASA)	Subnational	both	60-79	60-79	624	535	Yes	Digital oscillometric	8
743	Netherlands	2009-2010	Measuring the Netherlands (NL de Maat)	Subnational	both	30-70	30-70	1,950	1,755	Yes	Digital oscillometric	
744	Netherlands	2009-2011	The Rotterdam Study, first subcohort	Community	urban	72-79	72-79	573	427	No	Digital oscillometric	
745	Netherlands	2011-2012	The Rotterdam Study, second subcohort	Community	urban	65-79	65-79	821	651	No	Digital oscillometric	
746	Netherlands	2011-2012	The Longitudinal Aging Study Amsterdam (LASA)	Subnational	both	63-79	63-79	474	417	Yes	Digital oscillometric	8
747	Netherlands	2011-2015	Healthy Life in an Urban Setting (HELIUS)	Community	urban	30-71	30-71	1,999	1,797	No	Digital oscillometric	
748	Netherlands	2012-2013	The Longitudinal Aging Study Amsterdam (LASA)	Subnational	both	55-65	55-65	447	429	Yes	Digital oscillometric	8
749	Netherlands	2012-2014	The Rotterdam Study, third subcohort	Community	urban	51-79	51-79	1,600	1,232	No	Digital oscillometric	
750	New Zealand	1993-1994	MONICA, Auckland	Community	urban	35-64	35-64	725	745	Yes	Random-zero mercury	
751	New Zealand	2008-2009	New Zealand Adult Nutrition Survey	National	both	30-79	30-79	1,515	1,246	Yes	Digital oscillometric	
752	New Zealand	2012-2013	New Zealand Health Survey	National	both	30-79	30-79	4,991	3,680	No	Digital oscillometric	
753	New Zealand	2013-2014	New Zealand Health Survey	National	both	30-79	30-79	5,133	4,117	Yes	Digital oscillometric	
754	New Zealand	2014-2015	New Zealand Health Survey	National	both	30-79	30-79	5,111	4,175	Yes	Digital oscillometric	
755	New Zealand	2015-2016	New Zealand Health Survey	National	both	30-79	30-79	5,177	4,172	Yes	Digital oscillometric	
756	Nicaragua	2003-2004	CAMDI	Community	urban	30-79	30-79	706	520	Yes	Unknown	

	Country	Data years	Survey/Study name/Citation	Level of representative-ness	Rural, urban, or both	Age range used in the global analysis		Sample size used in the global analysis		Information on diagnosis	Type of blood pressure measurement device*	Note
						Female	Male	Female	Male			
757	Niger	2007	STEPS	National	both	30-64	30-64	739	1,032	Yes	Digital oscillometric	
758	Nigeria	2007	Ibadan Study of Ageing	Subnational	both	60-79	60-79	630	482	Yes	Digital oscillometric	
759	Nigeria	2008	Ibadan Study of Ageing	Subnational	both	61-79	61-79	440	339	Yes	Digital oscillometric	
760	Nigeria	2009	Ibadan Study of Ageing	Subnational	both	62-79	62-79	391	301	Yes	Digital oscillometric	
761	Nigeria	2011	Community Health Plan - Kwara Central Survey	Community	rural	30-79	30-79	359	281	Yes	Digital oscillometric	
762	Nigeria	2013	Community Health Plan - Kwara Central Survey	Community	rural	30-79	30-79	329	250	No	Digital oscillometric	
763	Nigeria	2018	Hypertension Prevalence, Awareness, Treatment and Control in Rural Area, Nigeria	Community	rural	30-79	30-79	122	95	Yes	Digital oscillometric	
764	Nigeria	2017-2019	Removing the Mask on Hypertension (REMAH)	National	both	30-79	30-79	1,785	1,332	Yes	Standard mercury	
765	Niue	2011	STEPS	National	both	30-79	30-79	345	284	Yes	Digital oscillometric	
766	North Korea	2007	STEPS	Community	rural	25-64	25-64	1,176	1,155	No	Digital oscillometric	1
767	North Korea	2008	STEPS	Subnational	both	25-64	25-64	2,924	2,818	No	Unknown	1
768	Norway	1992-1993	The Hordaland Homocysteine Study 1925-1927 birth cohort	Community	urban	65-67	65-67	2,620	2,117	No	Digital oscillometric	
769	Norway	1992-1993	The Hordaland Homocysteine Study 1928-1949 birth cohort	Community	urban	43-64	43-64	347	334	No	Digital oscillometric	
770	Norway	1992-1993	The Hordaland Homocysteine Study 1950-1952 birth cohort	Subnational	urban	40-42	40-42	6,435	6,065	No	Digital oscillometric	
771	Norway	1994-1995	The Tromsø Study: Tromsø 4	Community	both	30-79	30-79	11,794	11,033	No	Digital oscillometric	
772	Norway	1995-1997	HUNT2 study	Subnational	rural	30-79	30-79	27,620	25,140	No	Digital oscillometric	
773	Norway	1997-1999	The Hordaland Health Study (HUSK) 1925-1927 birth cohort	Community	urban	70-74	70-74	1,842	1,468	No	Digital oscillometric	
774	Norway	2000-2003	The Oslo cohort (HUBRO), the Oppland and Hedmark cohort (OPPHED), and the Troms and Finnmark cohort (TROFINN) of Cohort of Norway (CONOR)	Subnational	both	30-76	30-76	19,979	16,589	No	Digital oscillometric	
775	Norway	2001-2002	The Tromsø Study: Tromsø 5, Tromsø Study Panel	Community	both	30-79	30-79	3,365	2,366	No	Digital oscillometric	
776	Norway	2006-2008	HUNT3 Study	Subnational	rural	30-79	30-79	23,389	20,052	No	Digital oscillometric	
777	Norway	2007-2008	The Tromsø Study: Tromsø 6	Community	both	30-79	30-79	6,463	5,744	No	Digital oscillometric	
778	Occupied Palestinian Territory	1996-1998	Kobar, rural	Community	rural	30-64	30-64	250	205	Yes	Standard mercury	
779	Occupied Palestinian Territory	1996-1998	Old Ramallah, urban	Community	urban	30-64	30-64	282	182	Yes	Standard mercury	
780	Occupied Palestinian Territory	1999-2000	The First National Health and Nutrition Survey	National	both	30-64	30-64	1,080	1,190	Yes	Random-zero mercury	
781	Occupied Palestinian Territory	2010	STEPS	National	both	30-64	30-64	3,003	1,594	Yes	Digital oscillometric	
782	Oman	2008	Gulf Cooperation Council World Health Survey	National	both	30-79	30-79	1,349	1,497	Yes	Digital oscillometric	
783	Oman	2017	STEPS	National	both	30-79	30-79	2,212	2,510	Yes	Digital oscillometric	
784	Pakistan	2005	STEPS	National	both	30-65	30-65	681	458	Yes	Digital oscillometric	
785	Pakistan	2014	STEPS	Subnational	both	30-69	30-69	2,295	2,031	Yes	Digital oscillometric	
786	Palau	2011-2013	STEPS	National	both	30-64	30-64	1,033	943	Yes	Digital oscillometric	
787	Palau	2016	STEPS	National	both	30-79	30-79	623	611	Yes	Digital oscillometric	
788	Panama	2010-2011	Prevalencia de factores de riesgo asociados a enfermedad cardiovascular	Subnational	both	30-79	30-79	1,922	867	Yes	Digital oscillometric	
789	Papua New Guinea	2007	STEPS	National	both	30-64	30-64	811	851	Yes	Digital oscillometric	
790	Paraguay	2011	Primera Encuesta Nacional de Factores de Riesgo de Enfermedades No Transmisibles en Poblacion General	National	both	30-75	30-75	1,042	579	Yes	Digital oscillometric	
791	Peru	2003	Factores de Riesgo de Enfermedades No Transmisibles (FRENT)	Community	urban	30-79	30-79	391	219	Yes	Standard mercury	
792	Peru	2004	Factores de Riesgo de Enfermedades No Transmisibles (FRENT)	Community	urban	30-79	30-79	308	139	Yes	Standard mercury	
793	Peru	2005	Factores de Riesgo de Enfermedades No Transmisibles (FRENT)	Community	urban	30-79	30-79	376	124	Yes	Standard mercury	
794	Peru	2004-2005	CArdiovascular Risk factors Multiple Evaluation in Latin America (CARMELA)	Community	urban	25-64	25-64	883	769	Yes	Standard mercury	1
795	Peru	2004-2005	Encuesta Nacional de Indicadores Nutricionales, Bioquimicos, Socioeconomicos y Culturales Relacionados con las Enfermedades Cronicas Degenerativas (ENIN)	National	both	30-79	30-79	1,517	1,573	Yes	Standard mercury	
796	Peru	2006	Factores de Riesgo de Enfermedades No Transmisibles (FRENT)	Community	urban	30-79	30-79	770	397	Yes	Standard mercury	
797	Peru	2007-2008	PERU MIGRANT Study	Community	both	30-79	30-79	516	460	Yes	Digital oscillometric	
798	Peru	2009-2012	CRONICAS Cohort Study	Subnational	both	35-79	35-79	1,609	1,494	Yes	Digital oscillometric	
799	Peru	2010-2013	CRONICAS Cohort Study	Subnational	both	35-79	35-79	1,412	1,314	No	Digital oscillometric	
800	Peru	2012	Demographic and Health Survey Peru 2012	National	both	40-79	40-79	14,872	13,488	Yes	Digital oscillometric	
801	Peru	2013	Demographic and Health Survey Peru 2013	National	both	30-79	30-79	2,182	2,015	Yes	Digital oscillometric	
802	Peru	2012-2013	PERU MIGRANT Study	Community	both	35-79	35-79	456	370	No	Digital oscillometric	
803	Peru	2014	Demographic and Health Survey Peru 2014	National	both	30-79	30-79	9,733	8,643	Yes	Digital oscillometric	
804	Peru	2014	Launching a salt substitute to reduce blood pressure at the population level: a cluster randomized stepped wedge trial in Peru	Community	both	30-79	30-79	821	830	Yes	Digital oscillometric	
805	Peru	2013-2014	CRONICAS Cohort Study	Subnational	both	36-79	36-79	1,303	1,226	No	Digital oscillometric	

	Country	Data years	Survey/Study name/Citation	Level of representative-ness	Rural, urban, or both	Age range used in the global analysis		Sample size used in the global analysis		Information on diagnosis	Type of blood pressure measurement device*	Note
						Female	Male	Female	Male			
806	Peru	2015	Demographic and Health Survey Peru 2015	National	both	30-79	30-79	11,055	9,688	Yes	Digital oscillometric	
807	Peru	2016	Demographic and Health Survey Peru 2016	National	both	30-79	30-79	11,119	9,364	Yes	Digital oscillometric	
808	Peru	2015-2016	PERU MIGRANT Study	Community	both	38-79	38-79	421	328	Yes	Digital oscillometric	
809	Peru	2017	Demographic and Health Survey Peru 2017	National	both	30-79	30-79	11,391	9,552	Yes	Digital oscillometric	
810	Peru	2016-2017	Screening of T2DM	Community	urban	30-69	30-69	809	798	Yes	Digital oscillometric	
811	Peru	2018	Demographic and Health Survey Peru 2018	National	both	30-79	30-79	12,295	10,150	Yes	Digital oscillometric	
812	Peru	2019	Demographic and Health Survey Peru 2019	National	both	30-79	30-79	11,997	9,700	Yes	Digital oscillometric	
813	Philippines	2002	Cebu Longitudinal Health and Nutrition Survey 2002 Mother Follow-up	Community	both	32-66		2,079		Yes	Standard mercury	
814	Philippines	2003	6th National Nutrition Survey	National	both	30-79	30-79	2,019	1,758	Yes	Standard mercury	
815	Philippines	2005	Cebu Longitudinal Health and Nutrition Survey 2005 Mother Follow-up	Community	both	35-69		2,000		Yes	Standard mercury	
816	Philippines	2013-2014	8th National Nutrition Survey	National	both	30-79	30-79	7,746	6,856	Yes	Digital oscillometric	
817	Philippines	2015	2015 Updating of Nutritional Status of Filipino Children and Other Population Groups	National	both	30-79	30-79	35,553	29,991	Yes	Digital oscillometric	
818	Poland	1993	MONICA, Warsaw	Community	urban	35-64	35-64	762	751	Yes	Standard mercury	
819	Poland	1992-1993	MONICA, Tarnobrzeg Voivodship	Community	rural	35-64	35-64	663	576	Yes	Standard mercury	
820	Poland	1995-1996	Polish Program CINDI (CINDI Łodz 1995)	Community	urban	30-64	30-64	1,060	675	Yes	Digital oscillometric	
821	Poland	1997	NATPOL	National	both	30-79	30-79	566	491	Yes	Standard mercury	
822	Poland	2000	The health status, risk factors of chronic diseases and health behaviors of residents of Torun (CINDI Torun 2000)	Community	urban	30-79	30-79	788	730	Yes	Digital oscillometric	
823	Poland	2001-2002	The health status, risk factors of chronic diseases and health behaviors of residents of Lodz (CINDI Łodz 2001)	Community	urban	30-64	30-64	580	736	Yes	Digital oscillometric	
824	Poland	2002	The health status, risk factors of chronic diseases and health behaviors of residents of Lodz - seniors (CINDI Łodz 2002)	Community	urban	65-79	65-79	508	274	Yes	Digital oscillometric	
825	Poland	2002	NATPOL	National	both	30-79	30-79	1,059	798	Yes	Digital oscillometric	
826	Poland	2003	The European Male Ageing Study	Community	both		40-79		390	Yes	Digital oscillometric	
827	Poland	2002-2005	Health, Alcohol and Psychosocial factors In Eastern Europe (HAPIEE)	Community	urban	45-70	45-70	4,717	4,464	Yes	Digital oscillometric	
828	Poland	2003-2005	National Multicenter Health Survey in Poland. Project WOBASZ	National	both	30-74	30-74	5,608	5,054	Yes	Digital oscillometric	
829	Poland	2003-2006	Mogielica Human Ecology Study Site	Community	rural	30-79	30-79	247	96	Yes	Digital oscillometric	
830	Poland	2006	The health, risk factors for chronic diseases, attitudes and behaviors of health residents of Torun (CINDI Torun 2006)	Community	urban	30-65	30-65	889	595	Yes	Digital oscillometric	
831	Poland	2008	The European Male Ageing Study	Community	both		40-79		204	Yes	Digital oscillometric	
832	Poland	2007-2011	Medical, psychological and socioeconomic aspects of aging in Poland	National	both	55-79	55-79	1,602	1,587	Yes	Digital oscillometric	
833	Poland	2007-2010	Mogielica Human Ecology Study Site	Community	rural	30-79	30-79	233	117	Yes	Digital oscillometric	
834	Poland	2011	NATPOL	National	both	30-79	30-79	977	928	Yes	Digital oscillometric	
835	Poland	2011-2014	Mogielica Human Ecology Study Site	Community	rural	30-79	30-79	386	132	Yes	Digital oscillometric	
836	Poland	2013-2014	National Multicenter Health Survey in Poland. Project WOBASZ II	National	both	30-79	30-79	2,816	2,266	Yes	Digital oscillometric	
837	Poland	2015-2016	LIPIDOGRAM2015 & LIPIDOGEN2015 Study - National epidemiological study of lipid disorders and selected risk factors of cardiovascular disease in primary health care in Poland	National	both	30-79	30-79	8,055	4,683	Yes	Standard mercury	
838	Poland	2018	Mogielica Human Ecology Study Site	Community	rural	30-79	30-69	85	27	Yes	Digital oscillometric	
839	Portugal	1999-2003	EPIPorto Study	Community	urban	30-79	30-79	1,281	773	Yes	Aneroid	
840	Portugal	2015	Inquérito Nacional de Saúde com Exame Físico (INSEF)	National	both	30-74	30-74	2,453	2,108	Yes	Digital oscillometric	
841	Qatar	2006	World Health Survey	National	both	30-79	30-79	1,501	1,478	Yes	Unknown	
842	Qatar	2012	STEPS	National	both	30-64	30-64	985	743	Yes	Digital oscillometric	
843	Romania	2011-2012	Study for the Evaluation of Prevalence of Hypertension and cArdiovascular Risk among the Adult Population of Romania - SEPHAR II	National	both	30-79	30-79	905	798	Yes	Digital oscillometric	
844	Romania	2015-2016	Study for the Evaluation of Prevalence of Hypertension and cArdiovascular Risk among the Adult Population of Romania - SEPHAR III	National	both	30-79	30-79	837	769	Yes	Digital oscillometric	
845	Russian Federation	1992	Russian Karelia Survey in Pitkaranta	Community	both	30-64	30-64	414	348	Yes	Standard mercury	
846	Russian Federation	1992-1995	MONICA, Moscow, Leninsky district	Community	urban	35-64	35-64	858	537	Yes	Standard mercury	
847	Russian Federation	1992-1995	MONICA, Moscow (control)	Community	urban	35-64	35-64	527	557	Yes	Standard mercury	
848	Russian Federation	1994-1995	MONICA, Novosibirsk (intervention)	Community	urban	30-64	30-64	764	723	Yes	Standard mercury	
849	Russian Federation	1995	MONICA, Novosibirsk, Kirowsky district	Community	urban	30-64	30-64	666	674	Yes	Standard mercury	
850	Russian Federation	1997	Russian Karelia Survey in Pitkaranta	Community	both	30-64	30-64	381	273	Yes	Standard mercury	
851	Russian Federation	2002	Russian Karelia Survey in Pitkaranta	Community	both	30-64	30-64	288	201	Yes	Standard mercury	
852	Russian Federation	2002-2005	Health, Alcohol and Psychosocial factors In Eastern Europe (HAPIEE)	Community	urban	43-73	43-73	5,091	4,258	Yes	Digital oscillometric	
853	Russian Federation	2007	Russian Karelia Survey in Pitkaranta	Community	both	30-64	30-64	246	155	Yes	Standard mercury	
854	Russian Federation	2007-2010	SAGE	National	both	50-79	50-79	2,175	1,243	Yes	Digital oscillometric	

	Country	Data years	Survey/Study name/Citation	Level of representative-ness	Rural, urban, or both	Age range used in the global analysis		Sample size used in the global analysis		Information on diagnosis	Type of blood pressure measurement device*	Note
						Female	Male	Female	Male			
855	Russian Federation	2008-2009	Izhevsk Family Study II	Community	urban		25-60		1,061	Yes	Digital oscillometric	1
856	Russian Federation	2012-2014	Epidemiology of Cardiovascular Diseases in Different Regions of Russia (ESSE-RF)	National	both	30-64	30-64	10,899	6,211	Yes	Digital oscillometric	
857	Russian Federation	2015-2017	Ural Eye and Medical Study (UEMS)	Community	urban	40-79	40-79	1,361	1,005	Yes	Digital oscillometric	
858	Russian Federation	2015-2017	Ural Eye and Medical Study (UEMS)	Subnational	rural	40-79	40-79	1,827	1,495	Yes	Digital oscillometric	
859	Russian Federation	2017	Epidemiology of Cardiovascular Diseases in Different Regions of Russia - 2 (ESSE-RF-2)	Subnational	both	30-64	30-64	3,239	2,547	Yes	Digital oscillometric	
860	Rwanda	2012	STEPS	National	both	30-64	30-64	2,718	1,649	Yes	Digital oscillometric	
861	Saint Kitts and Nevis	2007	STEPS	Subnational	both	30-64	30-64	665	380	Yes	Digital oscillometric	
862	Saint Lucia	2012	STEPS	National	both	30-64	30-64	986	596	Yes	Digital oscillometric	
863	Saint Vincent and the Grenadines	2013-2014	STEPS	National	both	30-69	30-69	1,452	1,226	Yes	Digital oscillometric	
864	Samoa	1995	McGarvey, Pac Health Dialog 2001; 8: 157-62	National	both	30-59	30-59	148	139	Yes	Standard mercury	
865	Samoa	2002	STEPS	National	both	30-64	30-64	1,107	927	Yes	Digital oscillometric	
866	Samoa	2010	Samoan Genome-Wide Association Study	National	both	30-65	30-65	1,795	1,218	Yes	Digital oscillometric	
867	Samoa	2013	STEPS	National	both	30-64	30-64	641	402	Yes	Digital oscillometric	
868	Sao Tome and Principe	2009	STEPS	National	both	30-64	30-64	995	738	Yes	Digital oscillometric	
869	Sao Tome and Principe	2019	STEPS	National	both	30-69	30-69	948	648	Yes	Digital oscillometric	
870	Saudi Arabia	2007	Gulf Cooperation Council World Health Survey	National	both	30-79	30-79	2,351	3,499	Yes	Unknown	
871	Saudi Arabia	2011-2013	Jeddah City Study	Community	urban	30-79	30-79	425	351	Yes	Standard mercury	
872	Saudi Arabia	2013	Saudi Health Information Survey	National	both	30-79	30-79	3,395	3,361	No	Digital oscillometric	
873	Senegal	2015	Les maladies chroniques au Sénégal: Une écologie de la santé comparative entre Dakar et Widou Thiengoly	Community	both	30-79	30-79	437	423	Yes	Digital oscillometric	
874	Serbia	1994-1995	MONICA, Novi Sad	Community	urban	30-64	30-64	601	533	Yes	Standard mercury	
875	Serbia	2000	Health Status, Health Needs and Utilization of Health Care of the Population of Serbia	National	both	30-79	30-79	4,131	3,371	Yes	Unknown	
876	Serbia	2006	The 2006 National Health Survey for the Population of Serbia	National	both	30-79	30-79	6,077	5,415	Yes	Standard mercury	
877	Serbia	2013	The National Health Survey of the Republic of Serbia, 2013	National	both	30-79	30-79	5,916	5,050	Yes	Digital oscillometric	
878	Seychelles	1989	Seychelles Heart Survey I	National	both	30-64	30-64	496	459	Yes	Standard mercury	2
879	Seychelles	1994	Seychelles Heart Survey II	National	both	30-64	30-64	496	444	Yes	Standard mercury	
880	Seychelles	2004	Seychelles Heart Survey III	National	both	30-64	30-64	617	513	Yes	Standard mercury	
881	Seychelles	2013-2014	Seychelles Heart Survey IV	National	both	30-64	30-64	631	486	Yes	Standard mercury	
882	Sierra Leone	2009	STEPS	National	both	30-64	30-64	1,657	1,785	Yes	Digital oscillometric	
883	Singapore	1993-1995	NUH Heart Study	National	both	30-79	30-79	475	476	No	Standard mercury	
884	Singapore	2004-2007	Combined Follow-up of Singapore Cardiovascular Cohort Study and Singapore Prospective Study	National	both	30-79	30-79	2,484	2,304	Yes	Standard mercury	
885	Singapore	2009	Social Isolation, Health and Lifestyles Survey (SIHLS) 2009	National	both	60-79	60-79	1,922	1,674	Yes	Digital oscillometric	
886	Singapore	2009-2011	The Singapore Chinese Eye Study	Community	both	40-79	40-79	1,627	1,624	Yes	Digital oscillometric	
887	Singapore	2012-2013	Singapore Health Study 2012	National	both	30-79	30-79	797	721	Yes	Standard mercury	
888	Singapore	2014-2015	Singapore Health 2	National	urban	30-79	30-79	818	656	Yes	Digital oscillometric	
889	Singapore	2015-2017	The Singapore Chinese Eye Study Follow-Up	Community	urban	50-79	50-79	1,277	1,207	Yes	Digital oscillometric	
890	Singapore	2016-2017	Transitions in Health, Employment, Social Engagement and Inter-generational Transfers in Singapore Study (THE SIGNS Study)	National	urban	60-79	60-79	1,776	1,656	Yes	Digital oscillometric	
891	Slovakia	2011-2012	European Health Examination Survey	National	both	30-64	30-64	841	672	Yes	Digital oscillometric	
892	Solomon Islands	2004	A genetic-ecological study of the risk factors for lifestyle-related diseases in Oceanian populations	Community	rural	30-74	30-74	69	67	Yes	Digital oscillometric	
893	Solomon Islands	2004	A genetic-ecological study of the risk factors for lifestyle-related diseases in Oceanian populations	Community	urban	30-79	30-79	77	80	Yes	Digital oscillometric	
894	Solomon Islands	2006	STEPS	Subnational	both	30-64	30-64	778	549	Yes	Digital oscillometric	
895	Solomon Islands	2009-2010	Influences of the large-scale disaster and related socioecological changes on people's nutritional status and health in the Western Solomon Islands	Subnational	urban	30-69	30-69	50	32	Yes	Digital oscillometric	
896	Solomon Islands	2009-2010	Influences of the large-scale disaster and related socioecological changes on people's nutritional status and health in the Western Solomon Islands	Subnational	rural	30-79	30-79	162	127	Yes	Digital oscillometric	
897	Solomon Islands	2015	STEPS	National	both	30-69	30-69	769	650	Yes	Digital oscillometric	
898	Somalia	2016	The prevalence of selected risk factors for non-communicable diseases in Hargeisa, Somaliland: a cross-sectional study	Community	urban	30-69	30-69	653	90	Yes	Digital oscillometric	
899	South Africa	1998	Demographic and Health Survey South Africa 1998	National	both	30-79	30-79	4,707	3,097	Yes	Digital oscillometric	
900	South Africa	2003	Demographic and Health Survey South Africa 2003	National	both	30-79	30-79	2,778	1,755	Yes	Digital oscillometric	
901	South Africa	2003-2004	Africa Centre Biomeasure Survey	Community	rural	30-49	30-49	1,422	615	No	Unknown	
902	South Africa	2007-2008	SAGE	National	both	50-79	50-79	1,938	1,440	Yes	Digital oscillometric	
903	South Africa	2008	National Income Dynamics Study Wave I	National	both	30-79	30-79	4,918	2,926	Yes	Digital oscillometric	

	Country	Data years	Survey/Study name/Citation	Level of representative-ness	Rural, urban, or both	Age range used in the global analysis		Sample size used in the global analysis		Information on diagnosis	Type of blood pressure measurement device*	Note
						Female	Male	Female	Male			
904	South Africa	2008-2009	Cape Town Bellville South Cohort Study - Baseline evaluation I	Community	urban	35-65	35-65	491	140	Yes	Digital oscillometric	
905	South Africa	2010-2011	National Income Dynamics Study Wave II	National	both	30-79	30-79	5,014	2,991	Yes	Digital oscillometric	
906	South Africa	2012	National Income Dynamics Study Wave III	National	both	30-79	30-79	6,216	3,673	Yes	Digital oscillometric	
907	South Africa	2014-2015	Health and Aging in Africa: A Longitudinal Study of an INDEPTH Community in South Africa (HAALSI)	Community	rural	40-79	40-79	2,273	2,019	Yes	Digital oscillometric	
908	South Africa	2014-2015	National Income Dynamics Study Wave IV	National	both	30-79	30-79	5,802	4,086	Yes	Digital oscillometric	
909	South Africa	2016	Demographic and Health Survey South Africa 2016	National	both	30-49	30-59	1,609	1,378	Yes	Digital oscillometric	
910	South Africa	2017	National Income Dynamics Study Wave V	National	both	30-79	30-79	7,044	4,585	Yes	Digital oscillometric	
911	South Korea	1998	Korea National Health and Nutrition Examination Survey	National	both	30-79	30-79	3,410	2,897	Yes	Standard mercury	
912	South Korea	2001	Korea National Health and Nutrition Examination Survey	National	both	30-79	30-79	2,746	2,152	Yes	Standard mercury	
913	South Korea	2002-2003	Korean National Health Insurance	National	both	40-79	40-79	2,452,337	2,967,098	No	Unknown	
914	South Korea	2005	Korea National Health and Nutrition Examination Survey	National	both	30-79	30-79	2,649	2,028	Yes	Standard mercury	
915	South Korea	2004-2005	Korean National Health Insurance	National	both	40-79	40-79	3,215,141	3,564,859	No	Unknown	
916	South Korea	2007	Korea National Health and Nutrition Examination Survey	National	both	30-79	30-79	1,442	1,066	Yes	Standard mercury	
917	South Korea	2006-2007	Korean National Health Insurance	National	both	40-79	40-79	4,537,140	4,509,556	No	Unknown	
918	South Korea	2008	Korea National Health and Nutrition Examination Survey	National	both	30-79	30-79	3,267	2,412	Yes	Standard mercury	
919	South Korea	2008-2009	Korean National Health Insurance	National	both	40-79	40-79	5,979,345	5,685,147	No	Unknown	
920	South Korea	2009	Korea National Health and Nutrition Examination Survey	National	both	30-79	30-79	3,536	2,677	Yes	Standard mercury	
921	South Korea	2010	Korea National Health and Nutrition Examination Survey	National	both	30-79	30-79	2,975	2,314	Yes	Standard mercury	
922	South Korea	2011	Korea National Health and Nutrition Examination Survey	National	both	30-79	30-79	2,936	2,206	Yes	Standard mercury	
923	South Korea	2010-2011	Korean National Health Insurance	National	both	40-79	40-79	6,970,325	6,566,814	No	Unknown	
924	South Korea	2012	Korea National Health and Nutrition Examination Survey	National	both	30-79	30-79	2,762	1,991	Yes	Standard mercury	
925	South Korea	2013	Korea National Health and Nutrition Examination Survey	National	both	30-79	30-79	2,576	1,892	Yes	Standard mercury	
926	South Korea	2012-2013	Korean National Health Insurance	National	both	40-79	40-79	7,584,522	7,126,274	No	Unknown	
927	South Korea	2014	Korea National Health and Nutrition Examination Survey	National	both	30-79	30-79	2,469	1,787	Yes	Standard mercury	
928	South Korea	2015	Korea National Health and Nutrition Examination Survey	National	both	30-79	30-79	2,483	1,858	Yes	Standard mercury	
929	South Korea	2014-2015	Korean National Health Insurance	National	both	40-79	40-79	8,095,759	7,697,138	No	Unknown	
930	South Korea	2016	Korea National Health and Nutrition Examination Survey	National	both	30-79	30-79	2,893	2,220	Yes	Standard mercury	
931	South Korea	2017	Korea National Health and Nutrition Examination Survey	National	both	30-79	30-79	2,850	2,277	Yes	Standard mercury	
932	South Korea	2016-2017	Korean National Health Insurance	National	both	40-79	40-79	8,754,456	8,313,873	No	Unknown	
933	Spain	1990	Banegas et al., Hypertension 1998; 32: 998-1002	National	both	35-65	35-65	1,211	810	Yes	Random-zero mercury	
934	Spain	1990-1992	MONICA, Catalonia	Subnational	both	30-64	30-64	990	1,527	Yes	Random-zero mercury	
935	Spain	1991-1993	Encuesta de Factores de Riesgo Cardiovascular en la Región de Murcia (Cardiovascular Risk Factors Survey)	Subnational	both	30-69	30-69	1,171	1,090	Yes	Standard mercury	
936	Spain	1994-1995	Encuesta de Nutrición y Salud Comunidad Valenciana 1994-95 (ENCV)	Subnational	urban	30-79	30-79	609	509	Yes	Digital oscillometric	
937	Spain	1994-1996	MONICA, Catalonia	Subnational	both	30-64	30-64	1,440	1,638	Yes	Random-zero mercury	
938	Spain	1996	de Pablos-Velasco et al., J Hypertens 2002; 20: 1965-71	Community	rural	30+	30+	385	305	No	Unknown	1
939	Spain	1999-2000	Factores de riesgo en las islas Baleares: Estudio CORSAIB	Subnational	both	35-74	35-74	855	801	Yes	Digital oscillometric	
940	Spain	2000-2001	Regidor et al., J Hum Hypertens 2006; 20: 73-82	National	both	60-79	60-79	1,986	1,184	Yes	Unknown	
941	Spain	2000-2001	EUREYE Study	Subnational	both	65-79	65-79	253	234	Yes	Digital oscillometric	
942	Spain	2001-2003	DIabetes, Nutrición y Obesidad en la población adulta de la Región de Murcia (DINO)	Subnational	both	30-79	30-79	591	497	Yes	Digital oscillometric	
943	Spain	2001-2002	Catalan Health Interview Survey	Subnational	both	30-74	30-74	602	500	Yes	Digital oscillometric	
944	Spain	2000-2005	CDC of the Canary Islands	Subnational	both	30-75	30-75	3,085	2,390	Yes	Digital oscillometric	
945	Spain	2003	The European Male Ageing Study	Community	both		40-79		402	Yes	Digital oscillometric	
946	Spain	2004	Vioque J et al., Obesity 2008; 16: 664-70	Community	urban	30-79	30-79	85	71	Yes	Digital oscillometric	
947	Spain	2003-2005	Registre Gironi del Cor (REGICOR)	Subnational	both	35-79	35-79	3,204	2,883	Yes	Digital oscillometric	
948	Spain	2004	Cardiovascular Risk Study in Castilla y León (RECCyL)	Subnational	both	30-79	30-79	1,505	1,426	Yes	Digital oscillometric	
949	Spain	2004-2006	PREVICTUS	National	both	60-79	60-79	3,322	2,987	No	Digital oscillometric	
950	Spain	2008	The European Male Ageing Study	Community	both		40-79		194	Yes	Digital oscillometric	
951	Spain	2007-2009	Harmonizing Equation of Risk in Mediterranean countries Extremadura (HERMEX)	Subnational	both	30-79	30-79	1,397	1,226	Yes	Digital oscillometric	
952	Spain	2009	Cardiovascular Risk Study in Castilla y León (RECCyL)	Subnational	both	30-79	30-79	1,220	1,019	Yes	Digital oscillometric	
953	Spain	2008-2010	Study on Nutrition and Cardiovascular Risk in Spain (ENRICA)	National	both	30-79	30-79	4,789	4,368	Yes	Digital oscillometric	
954	Spain	2014	Cardiovascular Risk Study in Castilla y León (RECCyL)	Subnational	both	30-79	30-79	1,129	943	Yes	Digital oscillometric	
955	Spain	2015	Study on Nutrition and Cardiovascular Risk in Spain (ENRICA)	National	both	65-79	65-79	654	611	Yes	Digital oscillometric	
956	Spain	2016-2017	Estudio de Nutrición y Riesgo Cardiovascular en España (ENRICA)-Seniors cohort	Subnational	urban	65-79	65-79	1,332	1,242	Yes	Unknown	
957	Sri Lanka	2006	STEPS	National	both	30-64	30-64	4,373	4,313	No	Digital oscillometric	
958	Sri Lanka	2014	STEPS	National	both	30-69	30-69	2,509	1,632	Yes	Digital oscillometric	

	Country	Data years	Survey/Study name/Citation	Level of representative-ness	Rural, urban, or both	Age range used in the global analysis		Sample size used in the global analysis		Information on diagnosis	Type of blood pressure measurement device*	Note
						Female	Male	Female	Male			
959	Sudan	2016	STEPS	National	both	30-69	30-69	3,113	1,963	Yes	Digital oscillometric	
960	Suriname	2013-2015	The Healthy Life in Suriname Study (HELISUR)	Subnational	urban	30-70	30-70	550	303	Yes	Digital oscillometric	
961	Sweden	1990	MONICA Northern Sweden	Subnational	both	30-64	30-64	718	690	Yes	Random-zero mercury	
962	Sweden	1990	MONICA Gothenburg	Community	urban	30-64	30-64	691	694	Yes	Random-zero mercury	
963	Sweden	1985-1996	EPIC Umea	Subnational	both	30-72	30-72	12,353	11,523	Yes	Standard mercury	
964	Sweden	1992-1993	Population Study of Women in Gothenburg	Community	urban	62-78		798		Yes	Standard mercury	
965	Sweden	1991-1995	Uppsala Longitudinal Study of Adult Men	Community	both		70-70		1,207	Yes	Standard mercury	
966	Sweden	1991-1996	Malmö Diet and Cancer	Community	urban	45-73	45-73	15,778	10,204	Yes	Standard mercury	
967	Sweden	1994	MONICA Northern Sweden	Subnational	both	30-74	30-74	872	843	Yes	Random-zero mercury	
968	Sweden	1995	MONICA Gothenburg	Community	urban	30-64	30-64	778	676	Yes	Random-zero mercury	
969	Sweden	1999	MONICA Northern Sweden	Subnational	both	30-74	30-74	859	815	Yes	Random-zero mercury	
970	Sweden	1997-2001	Uppsala Longitudinal Study of Adult Men	Community	both		77-77		764	No	Standard mercury	
971	Sweden	2003	The European Male Ageing Study	Community	both		40-79		387	Yes	Digital oscillometric	
972	Sweden	2001-2004	Prospective Investigation of the Vasculature in Uppsala Seniors (PIVUS)	Community	both	70-70	70-70	499	504	No	Standard mercury	
973	Sweden	2001-2004	Swedish INTERGENE Cohort Study	Subnational	both	30-76	30-76	1,799	1,609	Yes	Digital oscillometric	
974	Sweden	2004	Welin et al., BMC Public Health 2008; 8: 403	Community	urban	50-50	50-60	667	1,250	No	Unknown	
975	Sweden	2004	MONICA Northern Sweden	Subnational	both	30-75	30-75	911	855	Yes	Random-zero mercury	
976	Sweden	2004-2005	Population Study of Women in Gothenburg	Community	urban	38-50		461		Yes	Standard mercury	
977	Sweden	2006-2009	Prospective Investigation of the Vasculature in Uppsala Seniors (PIVUS)	Community	both	75-75	75-75	419	404	No	Standard mercury	
978	Sweden	2008	The European Male Ageing Study	Community	both		40-79		263	Yes	Digital oscillometric	
979	Sweden	2009	MONICA Northern Sweden	Subnational	both	30-74	30-74	757	761	Yes	Digital oscillometric	
980	Sweden	2011-2014	EpiHealth	National	both	45-75	45-75	6,054	4,730	No	Digital oscillometric	
981	Sweden	2014	MONICA Northern Sweden	Subnational	both	30-74	30-74	732	672	Yes	Digital oscillometric	
982	Sweden	2014-2016	Swedish INTERGENE Cohort Study	Subnational	urban	37-79	37-79	601	565	Yes	Digital oscillometric	
983	Sweden	2016-2017	Population Study of Women in Gothenburg	Community	urban	38-50		572		Yes	Standard mercury	
984	Switzerland	1992-1993	The Swiss MONICA Study Wave III	Subnational	both	30-74	30-74	1,532	1,414	Yes	Random-zero mercury	
985	Switzerland	2003-2006	Cohorte Lausannoise (CoLaus)	Community	urban	35-75	35-75	3,288	2,978	Yes	Digital oscillometric	
986	Switzerland	2007-2012	Bus Santé Study	Subnational	urban	30-74	30-74	2,437	2,354	Yes	Digital oscillometric	
987	Switzerland	2009-2012	Cohorte Lausannoise (CoLaus)	Community	urban	40-79	40-79	2,664	2,327	Yes	Digital oscillometric	
988	Switzerland	2013-2016	Bus Santé	Subnational	urban	30-74	30-74	1,806	1,667	Yes	Digital oscillometric	
989	Switzerland	2014-2017	Cohorte Lausannoise (CoLaus)	Community	urban	45-79	45-79	2,119	1,760	Yes	Digital oscillometric	
990	Syrian Arab Republic	2002	National survey on non-communicable diseases and factors affecting their development	National	both	30-64	30-64	3,072	2,397	No	Unknown	
991	Taiwan	1995	Lu et al., Gerontol A Biol Sci Med Sci 2000; 55: M463-8	Community	urban	65-79	65-79	331	426	No	Unknown	
992	Taiwan	1993-1996	Nutrition and Health Survey in Taiwan 1993-1996	National	both	30-79	30-79	1,955	1,983	Yes	Standard mercury	
993	Taiwan	2000	Social Environment and Biomarkers of Aging Study	National	both	50-79	50-79	392	538	Yes	Digital oscillometric	
994	Taiwan	1999-2000	Nutrition and Health Survey in Taiwan 1999-2000	National	both	65-79	65-79	674	723	Yes	Digital oscillometric	
995	Taiwan	2002	Taiwanese Survey on Hypertension, Hyperglycemia and Hyperlipidemia	National	both	30-79	30-79	2,633	2,393	No	Standard mercury	
996	Taiwan	2006	Social Environment and Biomarkers of Aging Study	National	both	53-79	53-79	420	483	Yes	Digital oscillometric	
997	Taiwan	2005-2008	Nutrition and Health Survey in Taiwan 2005-2008	National	both	30-79	30-79	1,100	1,060	Yes	Digital oscillometric	
998	Taiwan	2007	Taiwanese Survey on Hypertension, Hyperglycemia and Hyperlipidemia	National	both	30-79	30-79	2,025	1,713	No	Digital oscillometric	
999	Taiwan	2013-2016	Nutrition and Health Survey in Taiwan	National	both	30-79	30-79	1,268	1,203	Yes	Digital oscillometric	
1000	Tajikistan	2016	STEPS	National	both	30-69	30-69	1,160	781	Yes	Digital oscillometric	
1001	Tajikistan	2017	Demographic and Health Survey Tajikistan 2017	National	both	30-49		4,816		Yes	Digital oscillometric	
1002	Tanzania	1996	Edwards et al., J Hypertens 2000; 18: 145-52	Community	urban	35-54	35-54	89	89	No	Unknown	
1003	Tanzania	1997	Edwards et al., J Hypertens 2000; 18: 145-52	Community	rural	35-54	35-54	201	138	No	Unknown	
1004	Tanzania	1998-1999	Bovet et al., Int J Epidemiol 2002; 31: 240-7	Community	urban	30-64	30-64	3,193	2,423	Yes	Digital oscillometric	
1005	Tanzania	2011	STEPS	Subnational	both	30-64	30-64	1,231	814	Yes	Digital oscillometric	
1006	Tanzania	2012	STEPS	National	both	30-64	30-64	2,350	2,173	Yes	Digital oscillometric	
1007	Tanzania	2014	Dar es Salaam Urban Cohort Hypertension Study	Community	urban	40-79	40-79	1,247	989	Yes	Digital oscillometric	
1008	Tanzania	2018	A health needs assessment survey for the design and establishment of a Lown Community Health Center in Ukonga	Community	urban	30-79	30-79	260	87	Yes	Digital oscillometric	
1009	Thailand	1991	Thailand National Health Examination Survey I	National	both	30-79	30-79	5,566	4,067	Yes	Standard mercury	
1010	Thailand	2000	InterASIA	National	both	35-79	35-79	3,191	2,074	Yes	Standard mercury	
1011	Thailand	2004	Thailand National Health Examination Survey III	National	both	30-79	30-79	17,707	16,005	Yes	Standard mercury	
1012	Thailand	2009	Thailand National Health Examination Survey IV	National	both	30-79	30-79	8,869	7,930	Yes	Digital oscillometric	
1013	Timor-Leste	2014	STEPS	National	both	30-69	30-69	1,042	842	Yes	Digital oscillometric	
1014	Togo	2010	STEPS	National	both	30-64	30-64	1,156	1,192	Yes	Digital oscillometric	

	Country	Data years	Survey/Study name/Citation	Level of representative-ness	Rural, urban, or both	Age range used in the global analysis		Sample size used in the global analysis		Information on diagnosis	Type of blood pressure measurement device*	Note
						Female	Male	Female	Male			
1015	Tokelau	2005	STEPS	National	both	30-64	30-64	199	169	Yes	Digital oscillometric	
1016	Tokelau	2014	STEPS	National	both	30-64	30-64	196	167	Yes	Digital oscillometric	
1017	Tonga	2004	STEPS	National	both	30-64	30-64	447	307	Yes	Digital oscillometric	
1018	Tonga	2011	STEPS	National	both	30-64	30-64	1,256	804	Yes	Digital oscillometric	
1019	Tonga	2017	STEPS	National	both	30-69	30-69	1,838	1,014	Yes	Digital oscillometric	
1020	Trinidad and Tobago	2001	Adult Survey	National	rural	30-79	30-79	216	177	Yes	Digital oscillometric	
1021	Trinidad and Tobago	2011	STEPS	National	both	30-64	30-64	1,146	721	Yes	Digital oscillometric	
1022	Tunisia	1996-1997	Tunisian National Nutrition Survey 1996-1997	National	both	30-79	30-79	1,899	1,077	Yes	Aneroid	
1023	Tunisia	1996-1997	Ariana Healthy Project 1997	Community	both	35-65	35-65	2,709	2,642	Yes	Standard mercury	
1024	Tunisia	2000	Laouani et al., Tunis Med 2004; 82: 1001-5	Community	urban	60-79	60-79	257	224	No	Unknown	
1025	Tunisia	2001	Ben Romdhane et al., Tunis Med 2005; 83 Suppl 5: 41-6	Community	both	40-69	40-69	919	917	No	Unknown	
1026	Tunisia	2005	Tunisian National Survey 2005 (TAHINA)	National	both	35-71	35-71	4,409	3,301	Yes	Standard mercury	
1027	Tunisia	2009-2010	ObeMaghreb	Subnational	urban	30-49	30-49	189	693	Yes	Aneroid	
1028	Tunisia	2016	Tunisian Health Examination Survey	Community	both	30-79	30-79	3,810	3,425	Yes	Digital oscillometric	
1029	Turkey	1998	Turkish Adult Risk Factor Study	National	both	30-79	30-79	740	685	Yes	Standard mercury	
1030	Turkey	1999	Tugay Aytekin et al., Health Soc Care Community 2002; 10: 394-401	Subnational	urban	30-79	30-79	1,017	932	No	Unknown	
1031	Turkey	2000	Turkish Adult Risk Factor Study	National	both	30-79	30-79	931	879	Yes	Standard mercury	
1032	Turkey	2001-2002	Turkish Adult Risk Factor Study	National	both	32-79	32-79	1,226	1,103	Yes	Aneroid	
1033	Turkey	2003	Prevalence, awareness, treatment and control of hypertension in Turkey in 2003	National	both	30-79	30-79	2,047	1,425	Yes	Random-zero mercury	
1034	Turkey	2003-2004	Turkish Adult Risk Factor Study	National	both	34-79	34-79	1,093	1,008	Yes	Aneroid	
1035	Turkey	2003-2005	Prevalence of prehypertension and associated risk factors among Turkish adults: Trabzon Hypertension Study	Subnational	both	30-79	30-79	1,889	1,590	Yes	Aneroid	
1036	Turkey	2005-2006	Turkish Adult Risk Factor Study	National	both	35-79	35-79	1,017	942	Yes	Aneroid	
1037	Turkey	2007-2008	Turkish Adult Risk Factor Study	National	both	37-79	37-79	1,068	1,043	Yes	Aneroid	
1038	Turkey	2009-2010	Turkish Adult Risk Factor Study	National	both	39-79	39-79	1,429	1,343	Yes	Aneroid	
1039	Turkey	2011	Chronic Diseases and Risk Factors Survey in Turkey	National	both	30-79	30-79	5,994	5,422	Yes	Digital oscillometric	
1040	Turkey	2012-2013	Turkish Adult Risk Factor Study	National	both	40-79	37-79	1,058	977	No	Aneroid	
1041	Turkey	2017	STEPS	National	both	30-79	30-79	2,705	1,758	Yes	Digital oscillometric	
1042	Turkmenistan	2013	STEPS	National	both	30-64	30-64	1,998	1,333	Yes	Digital oscillometric	
1043	Turkmenistan	2018	STEPS	National	both	30-69	30-69	1,717	1,283	Yes	Digital oscillometric	
1044	Tuvalu	2015	STEPS	National	both	30-69	30-69	443	361	Yes	Digital oscillometric	
1045	Uganda	2011-2012	The Prevalence and Distribution of Non-communicable Diseases and Their Risk Factors in Kasese District, Uganda	Subnational	both	30-79	30-79	187	225	Yes	Digital oscillometric	
1046	Uganda	2014	STEPS	National	both	30-69	30-69	1,333	927	Yes	Digital oscillometric	
1047	Ukraine	2007	Demographic and Health Survey Ukraine 2007	National	both	30-49	30-49	3,256	1,393	Yes	Digital oscillometric	
1048	Ukraine	2019	STEPS	National	both	30-69	30-69	2,283	1,292	Yes	Digital oscillometric	
1049	United Arab Emirates	2009	Gulf Cooperation Council World Health Survey	National	both	30-79	30-79	475	456	Yes	Unknown	
1050	United Arab Emirates	2017-2018	STEPS	National	both	30-79	30-79	1,823	1,781	Yes	Digital oscillometric	
1051	United Kingdom	1986-1987	Dietary and Nutritional Survey of British Adults 1986-1987	National	both	30-64	30-64	601	696	No	Digital oscillometric	2
1052	United Kingdom	1989	MRC National Survey of Health and Development	National	both	42-44	42-44	1,557	1,571	Yes	Random-zero mercury	2
1053	United Kingdom	1992	MONICA, Glasgow	Community	urban	30-64	30-64	688	622	Yes	Random-zero mercury	
1054	United Kingdom	1991-1992	MONICA, Belfast	Subnational	both	30-64	30-64	900	923	Yes	Random-zero mercury	
1055	United Kingdom	1991-1992	Health Survey for England (HSE)	National	both	30-79	30-79	2,426	2,155	Yes	Digital oscillometric	
1056	United Kingdom	1993	Health Survey for England (HSE)	National	both	30-79	30-79	5,532	5,016	Yes	Digital oscillometric	
1057	United Kingdom	1992-1994	Edinburgh Artery Study	Community	urban	60-79	60-79	560	551	No	Random-zero mercury	
1058	United Kingdom	1994	Health Survey for England (HSE)	National	both	30-79	30-79	5,195	4,612	Yes	Digital oscillometric	
1059	United Kingdom	1995	MONICA, Glasgow	Community	urban	30-64	30-64	831	759	Yes	Random-zero mercury	
1060	United Kingdom	1994-1995	Hertfordshire Ageing Study	Subnational	both	63-73	63-73	302	403	Yes	Digital oscillometric	
1061	United Kingdom	1995	Scottish Health Survey (SHeS)	Subnational	both	30-64	30-64	2,578	2,330	Yes	Digital oscillometric	
1062	United Kingdom	1998	Health Survey for England (HSE)	National	both	30-79	30-79	5,500	4,702	Yes	Digital oscillometric	
1063	United Kingdom	1997-1999	INTERMAP, West Bromwich	Community	urban	40-59	40-59	138	141	Yes	Random-zero mercury	
1064	United Kingdom	1998	Scottish Health Survey (SHeS)	Subnational	both	30-74	30-74	3,139	2,660	Yes	Digital oscillometric	
1065	United Kingdom	1998-1999	INTERMAP, Belfast	Community	urban	40-59	40-59	97	125	Yes	Random-zero mercury	
1066	United Kingdom	1998-2000	The British Regional Heart Study	National	urban		60-79		4,063	Yes	Digital oscillometric	
1067	United Kingdom	1999	MRC National Survey of Health and Development	National	both	53-54	53-54	1,478	1,451	Yes	Digital oscillometric	
1068	United Kingdom	2000	Health Survey for England (HSE)	National	both	65-79	65-79	132	105	Yes	Digital oscillometric	
1069	United Kingdom	1999-2001	British Women's Heart and Health Study	National	both	60-79		3,791		Yes	Digital oscillometric	9

	Country	Data years	Survey/Study name/Citation	Level of representative-ness	Rural, urban, or both	Age range used in the global analysis		Sample size used in the global analysis		Information on diagnosis	Type of blood pressure measurement device*	Note
						Female	Male	Female	Male			
1070	United Kingdom	1999-2001	Edinburgh Artery Study	Community	urban	66-79	66-79	270	255	No	Random-zero mercury	
1071	United Kingdom	2001	Health Survey for England (HSE)	National	both	30-79	30-79	5,121	4,416	No	Digital oscillometric	
1072	United Kingdom	2000-2001	National Diet and Nutrition Survey 2000-2001	National	both	30-64	30-64	779	659	No	Digital oscillometric	
1073	United Kingdom	1999-2004	Hertfordshire Cohort Study	Subnational	both	60-73	59-73	1,405	1,561	Yes	Digital oscillometric	
1074	United Kingdom	2002	Health Survey for England (HSE)	National	both	30-79	30-79	2,606	2,021	No	Digital oscillometric	
1075	United Kingdom	2003	Scottish Health Survey (SHeS)	Subnational	both	30-79	30-79	2,323	1,936	Yes	Digital oscillometric	
1076	United Kingdom	2003	The European Male Ageing Study	Community	both		40-79		388	Yes	Digital oscillometric	
1077	United Kingdom	2003	Health Survey for England (HSE)	National	both	30-79	30-79	4,806	4,040	Yes	Digital oscillometric	
1078	United Kingdom	2003-2005	Hertfordshire Ageing Study	Subnational	both	72-79	72-79	116	163	Yes	Digital oscillometric	
1079	United Kingdom	2005	Health Survey for England (HSE)	National	both	65-79	65-79	1,256	1,153	Yes	Digital oscillometric	
1080	United Kingdom	2004-2005	English Longitudinal Study of Ageing Wave 2 2004-2005	National	both	52-79	52-79	2,737	2,296	Yes	Digital oscillometric	
1081	United Kingdom	2006	Health Survey for England (HSE)	National	both	30-79	30-79	3,961	3,296	Yes	Digital oscillometric	
1082	United Kingdom	2007	Health Survey for England (HSE)	National	both	30-79	30-79	2,107	1,736	No	Digital oscillometric	
1083	United Kingdom	2008	The European Male Ageing Study	Community	both		40-79		205	Yes	Digital oscillometric	
1084	United Kingdom	2008	Health Survey for England (HSE)	National	both	30-79	30-79	4,495	3,705	No	Digital oscillometric	
1085	United Kingdom	2008	Scottish Health Survey (SHeS)	Subnational	both	30-79	30-79	462	399	Yes	Digital oscillometric	
1086	United Kingdom	2009	Scottish Health Survey (SHeS)	Subnational	both	30-79	30-79	496	396	Yes	Digital oscillometric	
1087	United Kingdom	2009	Health Survey for England (HSE)	National	both	30-79	30-79	1,354	1,180	Yes	Digital oscillometric	
1088	United Kingdom	2006-2010	MRC National Survey of Health and Development	National	both	60-65	60-65	1,033	934	Yes	Digital oscillometric	
1089	United Kingdom	2010	Health Survey for England (HSE)	National	both	30-79	30-79	2,421	1,892	Yes	Digital oscillometric	
1090	United Kingdom	2008-2012	National Diet and Nutrition Survey (NDNS)	National	both	30-79	30-79	1,195	879	No	Digital oscillometric	
1091	United Kingdom	2010	Scottish Health Survey (SHeS)	Subnational	both	30-79	30-79	448	364	Yes	Digital oscillometric	
1092	United Kingdom	2011	Health Survey for England (HSE)	National	both	30-79	30-79	2,457	1,980	Yes	Digital oscillometric	
1093	United Kingdom	2011	Scottish Health Survey (SHeS)	Subnational	both	30-79	30-79	424	343	Yes	Digital oscillometric	
1094	United Kingdom	2012	Scottish Health Survey (SHeS)	Subnational	both	30-79	30-79	400	341	Yes	Digital oscillometric	
1095	United Kingdom	2012	Health Survey for England (HSE)	National	both	30-79	30-79	2,346	1,896	Yes	Digital oscillometric	
1096	United Kingdom	2013	Health Survey for England (HSE)	National	both	30-79	30-79	2,689	2,167	Yes	Digital oscillometric	
1097	United Kingdom	2013	Scottish Health Survey (SHeS)	Subnational	both	30-79	30-79	505	389	Yes	Digital oscillometric	
1098	United Kingdom	2013-2014	National Diet and Nutrition Survey (NDNS)	National	both	30-79	30-79	505	336	No	Digital oscillometric	
1099	United Kingdom	2014	Scottish Health Survey (SHeS)	Subnational	both	30-79	30-79	556	432	Yes	Digital oscillometric	
1100	United Kingdom	2014	Health Survey for England (HSE)	National	both	30-79	30-79	2,389	1,915	Yes	Digital oscillometric	
1101	United Kingdom	2015	Scottish Health Survey (SHeS)	Subnational	both	30-79	30-79	375	293	Yes	Digital oscillometric	
1102	United Kingdom	2015	Health Survey for England (HSE)	National	both	30-79	30-79	2,335	1,911	Yes	Digital oscillometric	
1103	United Kingdom	2016	Scottish Health Survey (SHeS)	Subnational	both	30-79	30-79	392	314	Yes	Digital oscillometric	
1104	United Kingdom	2016	Health Survey for England (HSE)	National	both	30-79	30-79	2,211	1,753	Yes	Digital oscillometric	
1105	United Kingdom	2015-2016	National Diet and Nutrition Survey (NDNS)	National	both	30-79	30-79	449	357	No	Digital oscillometric	
1106	United Kingdom	2017	Health Survey for England (HSE)	National	both	30-79	30-79	2,358	1,788	Yes	Digital oscillometric	
1107	United Kingdom	2016-2017	National Diet and Nutrition Survey (NDNS)	National	both	30-79	30-79	206	156	No	Digital oscillometric	
1108	United Kingdom	2017	Scottish Health Survey (SHeS)	Subnational	both	30-79	30-79	395	306	Yes	Digital oscillometric	
1109	United Kingdom	2018	Health Survey for England (HSE)	National	both	30-79	30-79	2,155	1,690	Yes	Digital oscillometric	
1110	United Kingdom	2018	Scottish Health Survey (SHeS)	Subnational	both	30-79	30-79	527	404	Yes	Digital oscillometric	
1111	United Kingdom	2019	Scottish Health Survey (SHeS)	Subnational	both	30-79	30-79	544	402	Yes	Digital oscillometric	
1112	United States of America	1989-1990	MONICA, Stanford	Subnational	urban	30-64	30-64	630	524	Yes	Standard mercury	10
1113	United States of America	1989-1990	Cardiovascular Health Study	Subnational	both	65-79	65-79	2,976	2,141	Yes	Random-zero mercury	
1114	United States of America	1988-1992	Framingham Heart Study (FHS)	Community	urban	68-79	69-79	476	295	No	Standard mercury	
1115	United States of America	1990-1991	Coronary Artery Risk Development in Young Adults (CARDIA)	Subnational	urban	30-35	30-35	1,343	1,097	Yes	Random-zero mercury	
1116	United States of America	1990-1992	Atherosclerosis Risk in Communities Study	Subnational	both	46-69	46-69	5,607	4,529	Yes	Random-zero mercury	
1117	United States of America	1990-1991	Cardiovascular Health Study	Subnational	both	65-79	65-79	2,360	1,724	Yes	Random-zero mercury	
1118	United States of America	1988-1994	US NHANES III	National	both	30-79	30-79	6,637	5,907	Yes	Standard mercury	
1119	United States of America	1990-1994	Framingham Heart Study (FHS)	Community	urban	70-79	72-79	357	204	No	Standard mercury	
1120	United States of America	1991-1992	Cardiovascular Health Study	Subnational	both	65-79	65-79	2,149	1,526	Yes	Random-zero mercury	
1121	United States of America	1991-1995	Framingham Heart Study (FHS) - Offspring Cohort	Community	urban	31-78	31-78	1,961	1,736	No	Standard mercury	
1122	United States of America	1992-1993	Cardiovascular Health Study	Subnational	both	65-79	65-79	352	215	Yes	Random-zero mercury	
1123	United States of America	1992-1993	Coronary Artery Risk Development in Young Adults (CARDIA)	Subnational	urban	30-37	30-37	1,641	1,321	Yes	Random-zero mercury	
1124	United States of America	1992-1996	Framingham Heart Study (FHS)	Community	urban	72-79	74-79	271	154	No	Standard mercury	
1125	United States of America	1993-1995	Atherosclerosis Risk in Communities Study	Subnational	both	50-73	50-73	4,987	3,977	Yes	Random-zero mercury	

	Country	Data years	Survey/Study name/Citation	Level of representative-ness	Rural, urban, or both	Age range used in the global analysis		Sample size used in the global analysis		Information on diagnosis	Type of blood pressure measurement device*	Note
						Female	Male	Female	Male			
1126	United States of America	1993-1994	Cardiovascular Health Study	Subnational	both	65-79	65-79	2,013	1,363	No	Random-zero mercury	
1127	United States of America	1994-1995	Cardiovascular Health Study	Subnational	both	66-79	66-79	1,961	1,296	No	Random-zero mercury	
1128	United States of America	1995-1996	Coronary Artery Risk Development in Young Adults (CARDIA)	Subnational	urban	30-40	30-40	1,938	1,576	Yes	Random-zero mercury	
1129	United States of America	1993-1998	Women's Health Initiative - Observational Study	National	both	49-79		92,271		Yes	Standard mercury	
1130	United States of America	1997-1998	INTERMAP, Chicago	Community	urban	40-59	40-59	159	156	Yes	Random-zero mercury	
1131	United States of America	1996-1997	INTERMAP, Jackson	Community	urban	40-59	40-59	134	132	Yes	Random-zero mercury	
1132	United States of America	1995-1998	Framingham Heart Study (FHS) - Offspring Cohort	Community	urban	37-79	37-79	1,813	1,584	No	Standard mercury	
1133	United States of America	1997-1998	INTERMAP, Corpus Christi	Community	urban	40-59	40-59	276	270	Yes	Random-zero mercury	
1134	United States of America	1996-1997	Study of Women's Health Across the Nation	Subnational	both	40-55		3,231		No	Standard mercury	11
1135	United States of America	1996-1997	INTERMAP, Baltimore	Community	urban	40-59	40-59	134	146	Yes	Random-zero mercury	
1136	United States of America	1996-1997	INTERMAP, Pittsburgh	Community	urban	40-59	40-59	128	132	Yes	Random-zero mercury	
1137	United States of America	1996-1998	INTERMAP, Minneapolis	Community	urban	40-59	40-59	130	130	Yes	Random-zero mercury	
1138	United States of America	1996-1998	Atherosclerosis Risk in Communities Study	Subnational	both	50-75	50-75	4,479	3,540	No	Random-zero mercury	
1139	United States of America	1996-1997	Cardiovascular Health Study	Subnational	both	67-80	67-80	1,520	982	No	Random-zero mercury	1
1140	United States of America	1995-1998	Framingham Heart Study (FHS)	Community	urban	74-79	76-79	131	72	No	Standard mercury	
1141	United States of America	1997-1999	Study of Women's Health Across the Nation	Subnational	both	40-55		2,837		Yes	Standard mercury	11
1142	United States of America	1997-1999	Framingham Heart Study (FHS)	Community	urban	76-79	78-79	54	24	No	Standard mercury	
1143	United States of America	1997-1998	Cardiovascular Health Study	Subnational	both	68-79	68-79	1,250	757	No	Random-zero mercury	
1144	United States of America	1998-1999	Cardiovascular Health Study	Subnational	both	69-80	69-80	1,191	717	No	Random-zero mercury	1
1145	United States of America	1998-2000	Study of Women's Health Across the Nation	Subnational	both	40-55		2,660		Yes	Standard mercury	11
1146	United States of America	1999-2001	Framingham Heart Study (FHS)	Community	urban	78-79		2		No	Standard mercury	
1147	United States of America	1998-2001	Framingham Heart Study (FHS) - Offspring Cohort	Community	urban	33-79	37-79	1,842	1,565	No	Standard mercury	
1148	United States of America	1999-2001	Study of Women's Health Across the Nation	Subnational	both	40-56		2,526		Yes	Standard mercury	11
1149	United States of America	1999-2000	US NHANES 1999-2000	National	both	30-79	30-79	1,536	1,514	Yes	Standard mercury	
1150	United States of America	2000-2001	Coronary Artery Risk Development in Young Adults (CARDIA)	Subnational	urban	33-45	33-45	1,961	1,554	Yes	Random-zero mercury	
1151	United States of America	2000-2002	Study of Women's Health Across the Nation	Subnational	both	40-57		2,457		Yes	Standard mercury	11
1152	United States of America	2001-2002	US NHANES 2001-2002	National	both	30-79	30-79	1,634	1,682	Yes	Standard mercury	
1153	United States of America	2002-2005	Framingham Heart Study (FHS) - Third Generation Cohort	Community	urban	30-69	30-69	1,884	1,649	No	Standard mercury	
1154	United States of America	2003-2004	US NHANES 2003-2004	National	both	30-79	30-79	1,497	1,547	Yes	Standard mercury	
1155	United States of America	2005-2006	Cardiovascular Health Study	Subnational	both	70-80	70-80	531	290	No	Random-zero mercury	1
1156	United States of America	2005-2006	US NHANES 2005-2006	National	both	30-79	30-79	1,487	1,611	Yes	Standard mercury	
1157	United States of America	2005-2006	Coronary Artery Risk Development in Young Adults (CARDIA)	Subnational	urban	38-50	38-50	1,994	1,507	Yes	Random-zero mercury	
1158	United States of America	2005-2008	Framingham Heart Study (FHS) - Offspring Cohort	Community	urban	40-79	43-79	1,455	1,206	No	Standard mercury	
1159	United States of America	2007-2008	US NHANES 2007-2008	National	both	30-79	30-79	2,127	2,073	Yes	Standard mercury	
1160	United States of America	2009-2010	US NHANES 2009-2010	National	both	30-79	30-79	2,211	2,173	Yes	Standard mercury	
1161	United States of America	2010-2011	Coronary Artery Risk Development in Young Adults (CARDIA)	Subnational	urban	43-55	43-55	1,956	1,495	Yes	Random-zero mercury	
1162	United States of America	2011-2013	Atherosclerosis Risk in Communities Study	Subnational	both	67-79	67-79	1,906	1,340	No	Random-zero mercury	
1163	United States of America	2011-2012	US NHANES 2011-2012	National	both	30-79	30-79	1,913	1,903	Yes	Standard mercury	
1164	United States of America	2013-2014	US NHANES 2013-2014	National	both	30-79	30-79	2,162	1,990	Yes	Standard mercury	
1165	United States of America	2015-2016	US NHANES 2015-2016	National	both	30-79	30-79	2,072	1,973	Yes	Standard mercury	
1166	United States of America	2017-2018	US NHANES 2017-2018	National	both	30-79	30-79	1,986	1,891	Yes	Standard mercury	
1167	Uruguay	2006	STEPS	National	both	30-64	30-64	563	223	Yes	Digital oscillometric	
1168	Uruguay	2011-2012	CESCAS Study	Community	urban	30-79	30-79	839	609	Yes	Unknown	
1169	Uruguay	2013	STEPS	National	urban	30-64	30-64	1,087	618	Yes	Digital oscillometric	
1170	Uruguay	2012-2016	Genotype, Phenotype and Environment of Hypertension in Uruguay (GEFA-HT-UY)	Community	urban	30-79	30-79	166	106	Yes	Standard mercury	
1171	Uzbekistan	2002	Demographic and Health Survey Uzbekistan 2002	National	both	30-49	30-59	2,421	1,118	Yes	Standard mercury	
1172	Uzbekistan	2014	STEPS	National	both	30-64	30-64	1,593	1,076	Yes	Digital oscillometric	
1173	Uzbekistan	2019	STEPS	National	both	30-69	30-69	1,977	1,286	Yes	Digital oscillometric	
1174	Vanuatu	2005	STEPS	Subnational	both	30-60	30-60	369	287	No	Digital oscillometric	
1175	Vanuatu	2011	STEPS	National	both	30-64	30-64	1,785	1,885	Yes	Digital oscillometric	
1176	Venezuela	1998	Sulbaran et al., J Hum Hypertens 2000; 14 Suppl 1: S6-9	Community	urban	30-59	30-59	2,120	1,912	No	Unknown	
1177	Venezuela	2004-2005	CArdiovascular Risk factors Multiple Evaluation in Latin America (CARMELA)	Community	urban	25-64	25-64	1,135	713	Yes	Standard mercury	1
1178	Venezuela	2005-2006	Brajkovich et al., Rev Ven Endoc Metab 2006; 4: 31-2	Community	urban	30-65	30-65	196	90	Yes	Aneroid	
1179	Venezuela	2007-2008	Venezuelan Study of Metabolic Syndrome, Obesity and Lifestyle (VEMSOLS)	Community	urban	30-79	30-79	204	87	Yes	Aneroid	
1180	Venezuela	2008-2009	Venezuelan Study of Metabolic Syndrome, Obesity and Lifestyle (VEMSOLS)	Community	rural	30-79	30-79	58	33	No	Aneroid	

	Country	Data years	Survey/Study name/Citation	Level of representative-ness	Rural, urban, or both	Age range used in the global analysis		Sample size used in the global analysis		Information on diagnosis	Type of blood pressure measurement device*	Note
						Female	Male	Female	Male			
1181	Venezuela	2010-2011	Venezuelan Study of Metabolic Syndrome, Obesity and Lifestyle (VEMSOLS)	Community	urban	30-79	30-79	152	46	Yes	Aneroid	
1182	Venezuela	2015-2017	Cardio-Metabolic Health Venezuelan Study (EVESCAM)	National	both	30-79	30-79	1,955	879	Yes	Digital oscillometric	
1183	Viet Nam	2001-2003	The National Epidemiological Survey on Hypertension and Its Risk Factors (North)	Subnational	both	30-74	30-74	3,181	2,124	Yes	Digital oscillometric	
1184	Viet Nam	2004	The Hypertension Management Programme in Rural Communes (Hanoi)	Community	rural	30-74	30-74	1,204	795	Yes	Digital oscillometric	
1185	Viet Nam	2003-2004	The Survey on Heart Failure and Its Risk Factors	Subnational	both	30-74	30-74	2,346	1,662	Yes	Digital oscillometric	
1186	Viet Nam	2005	The Survey on Non-Communicable Disease Risk Factors	Subnational	both	30-74	30-74	1,117	1,031	Yes	Digital oscillometric	
1187	Viet Nam	2005	Non-communicable disease risk factors in Ho Chi Minh City	Community	urban	30-64	30-64	958	796	No	Digital oscillometric	
1188	Viet Nam	2005	National Adult Overweight Survey	National	both	30-65	30-65	8,579	8,385	Yes	Standard mercury	
1189	Viet Nam	2006	The Hypertension Management Programme in Rural Communes (Bavi)	Community	rural	30-74	30-74	599	378	Yes	Digital oscillometric	
1190	Viet Nam	2006-2008	The National Epidemiological Survey on Hypertension and Its Risk Factors (South)	Subnational	both	30-74	30-74	1,909	1,203	Yes	Digital oscillometric	
1191	Viet Nam	2007	The Hypertension Management Programme in Rural Communes (Phu Phuong)	Community	rural	30-74	30-74	565	352	Yes	Digital oscillometric	
1192	Viet Nam	2009	STEPS	National	both	30-64	30-64	6,951	6,054	Yes	Digital oscillometric	
1193	Viet Nam	2009	The Hypertension Management Programme in Rural Communes (Phu Cuong)	Community	rural	30-74	30-74	635	356	Yes	Digital oscillometric	
1194	Viet Nam	2008-2009	The survey on diabetes and its risk factors (DM-S)	Subnational	both	30-79	30-79	1,332	763	Yes	Digital oscillometric	
1195	Viet Nam	2012	National Survey of Diabetes in Vietnam	National	both	30-69	30-69	5,854	5,319	Yes	Digital oscillometric	
1196	Viet Nam	2015	STEPS	National	both	30-69	30-69	1,470	1,096	Yes	Digital oscillometric	
1197	Yemen	2007-2009	Hypertension and Diabetes in Yemen (HYDY)	National	urban	30-70	30-70	1,731	1,694	Yes	Digital oscillometric	
1198	Yemen	2007-2009	Hypertension and Diabetes in Yemen (HYDY)	National	rural	30-70	30-70	1,713	1,702	Yes	Digital oscillometric	
1199	Zambia	2008	STEPS	Subnational	urban	30-79	30-79	812	414	Yes	Digital oscillometric	
1200	Zambia	2017	STEPS	National	both	30-69	30-69	1,553	973	Yes	Digital oscillometric	
1201	Zimbabwe	1995	Mufunda et al., J Hum Hypertens 2000; 14: 65-73	Community	both	25-54	25-54	298	284	No	Unknown	1

* Blood pressure measurement devices include:
Standard: standard mercury sphygmomanometer or aneroid sphygmomanometer; Random-zero: random-zero mercury sphygmomanometer; Digital: digital oscillometric device; Unknown: information unavailable or multiple types of devices used.

1. The first age group started from <30 years old and/or the last age group ended ≥80 years, but had a mean age of 30-79 years.

2. National studies for the 3 years prior to 1990 were assigned to 1990 so that they can inform the estimates in countries with slightly earlier national data.

3. This research uses data from Australia Health Survey (AHS). We thank the Health Section, Australian Bureau of Statistics, Belconnen, ACT, Australia for support for AHS 2011-2012.

4. This research uses data from China Health and Nutrition Survey (CHNS). We thank the National Institute of Nutrition and Food Safety, China Center for Disease Control and Prevention, Carolina Population Center (5 R24 HD050924), the University of North Carolina at Chapel Hill, the NIH (R01 HD30880, DK056350, R24HD050924, and R01 HD38700) and the Fogarty International Center, NIH for financial support for the CHNS data collection and analysis files from 1989 to 2011 and future surveys, and the China Japan Friendship Hospital, Ministry of Health for support for CHNS 2009.

5. The bibliographic citation for this data source is: Pelaez, Martha, Alberto Palloni, Cecilia Albala, Juan C. Alfonso, Roberto Ham-Chande, Anselm Hennis, Maria Lucia Lebrao, Esther Lesn-Diaz, Edith Pantelides, and Omar Prats. SABE - SURVEY ON HEALTH, WELL-BEING, AND AGING IN LATIN AMERICA AND THE CARIBBEAN, 2000 [Computer file]. ICPSR version. Washington, D.C.: Pan American Health Organization/World Health Organization (PAHO/WHO) [producers], 2004. Ann Arbor, MI: Inter-university Consortium for Political and Social Research [distributor], 2005.

6. The authors thank the Heinz Nixdorf Foundation [Chairman: Martin Nixdorf; Past Chairman: Dr jur. Gerhard Schmidt], for their generous support of this study. Parts of the study were also supported by the German Research Council (DFG) [DFG project: EI 969/2-3, ER 155/6-1;6-2, HO 3314/2-1;2-2;2-3;4-3, INST 58219/32-1, JO 170/8-1, KN 885/3-1, PE 2309/2-1, SI 236/8-1;9-1;10-1,], the German Ministry of Education and Science [BMBF project: 01EG0401, 01GI0856, 01GI0860, 01GS0820_WB2-C, 01ER1001D, 01GI0205], the Ministry of Innovation, Science, Research and Technology, North Rhine-Westphalia (MIWFT-NRW), the Else Kröner-Fresenius-Stiftung [project: 2015_A119] and the German Social Accident Insurance [DGUV project: FF-FP295]. Furthermore the study was supported by the Competence Network for HIV/AIDS, the deanship of the University Hospital and IFORES of the University Duisburg-Essen, the European Union, the German Competence Network Heart Failure, Kulturstiftung Essen, the Protein Research Unit within Europe (PURE), the Dr. Werner-Jackstädt Stiftung and the following companies: Celgene GmbH München, Imatron/GE-Imatron, Janssen, Merck KG, Philips, ResMed Foundation, Roche Diagnostics, Sarstedt AG&Co, Siemens HealthCare Diagnostics, Volkswagen Foundation. The authors express their gratitude to all study participants of the Heinz Nixdorf Recall (HNR) Study, the personnel of the HNR study center and the EBT-scanner facilities, the investigative group and all former employees of the HNR study. The authors also thank the Advisory Board of the HNR Study: T. Meinertz, Hamburg, Germany (Chair); C. Bode, Freiburg, Germany; P.J. de Feyter, Rotterdam, Netherlands; B. Güntert, Hall i.T., Austria; F. Gutzwiller, Bern, Switzerland; H. Heinen, Bonn, Germany; O. Hess (†), Bern, Switzerland; B. Klein (†), Essen, Germany; H. Löwel, Neuherberg, Germany; M. Reiser, Munich, Germany; G. Schmidt (†), Essen, Germany; M. Schwaiger, Munich, Germany; C. Steinmüller, Bonn, Germany; T. Theorell, Stockholm, Sweden; and S.N Willich, Berlin, Germany.

7. The Older Persons in Jamaica Study was funded by the National Health Fund, Jamaica.

8. The Longitudinal Aging Study Amsterdam is supported by a grant from the Netherlands Ministry of Health Welfare and Sports, Directorate of Long-Term Care. The data collection in 2012-2013 and 2013-2014 was financially supported by the Netherlands Organization for Scientific Research (NWO) in the framework of the project "New Cohorts of young old in the 21st century" (file number 480-10-014).

9. The British Women's Heart and Health Study is supported by the British Heart Foundation (PG/13/66/30442). British Women's Heart and Health Study data are available to bona fide researchers for research purposes. Please refer to the BWHHS data sharing policy at http://www.ucl.ac.uk/british-womens-heart-health-study.

10. Prof Stephen Fortmann contributed data from the Stanford Five-City Project.

11. The bibliographic citation for this data source is: Sutton-Tyrrell, Kim, Faith Selzer, MaryFran Sowers, Robert Neer, Lynda Powell, Ellen Gold, Gail Greendale, Gerson Weiss, Karen Matthews, and Sonja McKinlay. Study of Women's Health Across the Nation (SWAN), 1996-1997: Baseline Dataset. ICPSR28762-v2. Ann Arbor, MI: Inter-university Consortium for Political and Social Research[distributor], 2014-02-04. http://doi.org/10.3886/ICPSR28762.v2.

Appendix Table 2. List of analysis regions and "super-regions", and countries in each region. The hierarchical structure of the statistical model consisted of country, region, super-region, and world.

Super-region	Region
**Sub-Saharan Africa (49)**	**Central Africa (6)**: Angola, Central African Republic, Congo, DR Congo, Equatorial Guinea, Gabon
	**East Africa (18)**: Burundi, Comoros, Djibouti, Eritrea, Ethiopia, Kenya, Madagascar, Malawi, Mauritius, Mozambique, Rwanda, Seychelles, Somalia, South Sudan, Sudan, Tanzania, Uganda, Zambia
	**Southern Africa (6)**: Botswana, Eswatini, Lesotho, Namibia, South Africa, Zimbabwe
	**West Africa (19)**: Benin, Burkina Faso, Cabo Verde, Cameroon, Chad, Cote d'Ivoire, Gambia, Ghana, Guinea, Guinea Bissau, Liberia, Mali, Mauritania, Niger, Nigeria, Sao Tome and Principe, Senegal, Sierra Leone, Togo
**Central Asia, Middle East and North Africa (28)**	**Central Asia (9)**: Armenia, Azerbaijan, Georgia, Kazakhstan, Kyrgyzstan, Mongolia, Tajikistan, Turkmenistan, Uzbekistan
	**Middle East and North Africa (19)**: Algeria, Bahrain, Egypt, Iran, Iraq, Jordan, Kuwait, Lebanon, Libya, Morocco, Occupied Palestinian Territory, Oman, Qatar, Saudi Arabia, Syrian Arab Republic, Tunisia, Turkey, United Arab Emirates, Yemen
**South Asia (6)**	**South Asia (6)**: Afghanistan, Bangladesh, Bhutan, India, Nepal, Pakistan, Sri Lanka
**East and Southeast Asia (15)**	**East Asia (3)**: China, North Korea, Taiwan
	**Southeast Asia (12)**: Brunei Darussalam, Cambodia, Indonesia, Lao PDR, Malaysia, Maldives, Myanmar, Philippines, Thailand, Timor-Leste, Viet Nam
**Oceania (17)**	**Polynesia and Micronesia (13)**: American Samoa, Cook Islands, French Polynesia, Kiribati, Marshall Islands, Micronesia (Federated States of), Nauru, Niue, Palau, Samoa, Tokelau, Tonga, Tuvalu
	**Melanesia (4):** Fiji, Papua New Guinea, Solomon Islands, Vanuatu
**High-income Asia Pacific (3)**	**High-income Asia Pacific (3)**: Japan, Singapore, South Korea
**Latin America and Caribbean (35)**	**Andean Latin America (3)**: Bolivia, Ecuador, Peru
	**Caribbean (18)**: Antigua and Barbuda, Bahamas, Barbados, Belize, Bermuda, Cuba, Dominica, Dominican Republic, Grenada, Guyana, Haiti, Jamaica, Puerto Rico, Saint Kitts and Nevis, Saint Lucia, Saint Vincent and the Grenadines, Suriname, Trinidad and Tobago
	**Central Latin America (9)**: Colombia, Costa Rica, El Salvador, Guatemala, Honduras, Mexico, Nicaragua, Panama, Venezuela

	**Southern Latin America (5)**: Argentina, Brazil, Chile, Paraguay, Uruguay
**High-income western (27)**	**High-income English-speaking countries* (6)**: Australia, Canada, Ireland, New Zealand, United Kingdom, United States of America
	**North Western Europe (12)**: Austria, Belgium, Denmark, Finland, Germany, Greenland, Iceland, Luxembourg, Netherlands, Norway, Sweden, Switzerland
	**South Western Europe (9)**: Andorra, Cyprus, France, Greece, Israel, Italy, Malta, Portugal, Spain
**Central and Eastern Europe (20)**	**Central Europe (13)**: Albania, Bosnia and Herzegovina, Bulgaria, Croatia, Czechia, Hungary, Montenegro, North Macedonia, Poland, Romania, Serbia, Slovakia, Slovenia
	**Eastern Europe (7)**: Belarus, Estonia, Latvia, Lithuania, Moldova, Russian Federation, Ukraine

* Although high-income English-speaking countries are geographically separated, they exhibit remarkably similar trends in cardiometabolic risk factors and outcomes.[1,15-18] They were therefore grouped together so that the statistical model shares information amongst them more than it does with other countries that are geographically closer but epidemiologically more distinct.

We did not have data on population by age group for American Samoa, Bermuda, Greenland, and Tokelau. Country-specific estimates were made but were not used in calculation of regional and global prevalences because the latter requires weighting by age-specific population.

Appendix Table 3. Estimated age-standardised prevalence of hypertension and proportions of people with hypertension who reported a diagnosis, who used treatment, and whose blood pressure was effectively controlled, by country in 2019. Numbers in parentheses are the 95% credible intervals.

Women aged 30-79 years	**Hypertension (%)**	**Detection/ awareness (%)**	**Treatment (%)**	**Control (%)**
**Central and Eastern Europe**				
Central Europe				
Albania	40.8 (32.7-48.9)	54.4 (41.1-67.0)	43.7 (29.7-57.7)	11.5 (4.6-21.9)
Bosnia and Herzegovina	41.3 (30.2-53.2)	64.5 (48.0-79.3)	56.7 (37.5-74.7)	21.5 (6.5-43.4)
Bulgaria	40.7 (26.9-55.6)	71.1 (48.2-89.3)	60.4 (31.7-84.4)	28.1 (6.7-59.7)
Croatia	45.3 (35.0-56.2)	78.9 (63.4-90.2)	62.1 (45.3-76.8)	24.7 (9.9-44.4)
Czechia	34.0 (27.3-41.2)	78.3 (68.7-86.5)	68.2 (56.1-78.5)	42.4 (27.4-58.2)
Hungary	40.9 (27.5-56.0)	71.2 (47.9-89.2)	60.2 (32.9-83.4)	28.3 (6.8-58.5)
Montenegro	40.8 (26.9-55.8)	71.3 (48.2-89.0)	60.4 (32.4-84.2)	28.5 (6.8-58.6)
North Macedonia	40.7 (27.4-55.7)	71.5 (47.5-89.1)	60.6 (32.4-84.3)	28.1 (6.7-59.7)
Poland	42.7 (35.5-50.1)	74.0 (64.0-82.7)	67.6 (56.4-77.4)	37.4 (23.0-52.9)
Romania	43.9 (35.1-52.8)	74.6 (61.8-85.4)	65.7 (50.8-78.9)	36.6 (20.3-55.0)
Serbia	42.1 (32.4-52.1)	74.5 (60.2-85.8)	67.7 (51.2-81.7)	31.3 (13.6-53.3)
Slovakia	38.0 (27.6-49.3)	78.1 (62.0-90.0)	69.9 (51.8-84.9)	40.6 (18.7-64.9)
Slovenia	40.7 (26.9-55.2)	71.1 (47.7-88.8)	60.2 (31.2-84.6)	28.4 (6.8-59.0)
Eastern Europe				
Belarus	46.6 (38.1-55.3)	75.3 (62.8-85.7)	56.6 (41.0-71.2)	13.7 (5.6-25.9)
Estonia	34.2 (23.6-46.2)	55.6 (36.7-73.3)	41.8 (23.4-62.1)	18.9 (5.6-39.3)
Latvia	38.9 (27.3-51.6)	72.8 (55.9-86.4)	57.3 (36.4-76.1)	19.9 (5.5-43.2)
Lithuania	42.0 (30.2-55.0)	74.1 (58.9-86.6)	52.9 (32.5-72.3)	15.8 (3.6-36.4)
Moldova	46.9 (35.4-58.6)	64.5 (46.7-79.8)	41.6 (24.5-59.8)	9.1 (2.2-22.1)
Russian Federation	41.2 (33.3-49.3)	80.9 (71.7-88.4)	57.0 (42.7-69.9)	21.4 (10.6-35.2)
Ukraine	41.6 (34.4-49.3)	73.1 (62.4-82.5)	58.9 (45.2-71.5)	17.3 (8.7-28.3)
**Central Asia, Middle East and North Africa**				
Central Asia				
Armenia	46.2 (36.9-55.5)	44.5 (29.9-59.0)	32.9 (19.7-47.5)	10.1 (3.3-20.7)
Azerbaijan	42.1 (33.4-51.3)	62.9 (48.6-75.8)	49.6 (34.6-64.4)	17.8 (7.8-30.9)
Georgia	42.3 (33.7-51.4)	68.4 (54.8-80.4)	54.0 (39.2-67.8)	20.5 (9.1-35.3)
Kazakhstan	42.5 (34.9-50.3)	86.4 (79.0-92.4)	74.3 (62.6-84.3)	34.5 (20.7-49.9)
Kyrgyzstan	43.2 (33.5-53.7)	61.1 (44.5-75.8)	49.0 (33.0-64.8)	11.3 (3.3-24.9)

Women aged 30-79 years	Hypertension (%)	Detection/ awareness (%)	Treatment (%)	Control (%)
Mongolia	40.7 (33.6-48.1)	76.8 (66.6-85.2)	63.8 (51.6-74.9)	32.7 (20.4-46.8)
Tajikistan	42.8 (35.3-50.4)	55.4 (42.0-67.9)	43.2 (30.2-56.5)	9.8 (4.2-18.4)
Turkmenistan	40.2 (32.9-47.7)	64.7 (53.0-74.9)	52.7 (40.3-65.1)	13.9 (6.5-24.4)
Uzbekistan	44.6 (37.3-52.2)	62.9 (51.5-73.5)	53.0 (40.1-65.5)	21.7 (12.7-33.0)
Middle East and North Africa				
Algeria	37.4 (29.6-45.7)	59.6 (46.3-72.3)	47.9 (34.1-61.6)	20.4 (9.8-34.4)
Bahrain	35.3 (23.5-49.2)	61.1 (38.1-81.8)	51.2 (24.8-76.8)	24.5 (5.5-52.6)
Egypt	40.7 (33.6-48.3)	61.4 (50.2-71.5)	52.2 (40.2-64.1)	22.9 (12.4-36.0)
Iran	25.8 (22.5-29.3)	69.2 (62.4-75.7)	57.9 (50.0-65.2)	29.8 (22.2-38.1)
Iraq	47.7 (37.3-58.2)	66.6 (51.1-79.9)	48.3 (31.6-64.4)	15.2 (5.5-28.7)
Jordan	35.7 (29.9-41.7)	71.7 (62.2-79.9)	66.0 (56.0-75.0)	35.9 (24.1-48.5)
Kuwait	35.2 (26.8-44.3)	73.4 (60.4-83.7)	66.7 (53.3-78.8)	38.6 (21.4-57.0)
Lebanon	34.2 (26.5-42.4)	61.3 (48.2-72.8)	54.6 (41.3-67.5)	31.8 (17.7-48.1)
Libya	39.4 (28.2-51.0)	56.6 (37.9-73.6)	43.1 (23.9-62.2)	15.1 (4.0-33.7)
Morocco	35.6 (27.7-44.2)	51.5 (37.5-65.6)	36.7 (23.1-51.6)	13.5 (5.8-24.6)
Occupied Palestinian Territory	37.4 (26.9-49.1)	53.2 (36.3-70.2)	50.7 (32.9-69.3)	20.7 (6.6-41.9)
Oman	38.8 (30.6-47.3)	50.7 (37.6-63.6)	44.9 (31.9-58.2)	19.9 (9.8-33.1)
Qatar	37.7 (28.0-48.3)	67.3 (52.0-80.7)	60.4 (44.0-75.7)	32.5 (14.5-54.7)
Saudi Arabia	30.2 (21.4-40.1)	60.1 (42.2-76.3)	49.5 (30.6-68.3)	25.6 (11.1-44.8)
Syrian Arab Republic	39.8 (27.7-53.2)	60.7 (36.3-81.3)	50.8 (24.3-76.5)	24.5 (5.6-53.5)
Tunisia	34.8 (25.6-45.0)	53.5 (39.2-67.5)	43.2 (27.4-59.7)	18.5 (6.5-36.2)
Turkey	34.4 (27.3-41.8)	69.4 (57.3-79.7)	63.8 (51.7-74.4)	35.9 (22.0-51.6)
United Arab Emirates	34.5 (26.9-42.4)	52.5 (39.6-65.2)	45.6 (32.4-59.0)	24.6 (13.2-38.6)
Yemen	29.6 (18.8-42.9)	49.3 (30.2-68.4)	39.3 (20.7-59.9)	19.8 (5.6-41.6)
**East and Southeast Asia**				
East Asia				
China	24.1 (18.4-30.4)	56.4 (44.7-67.2)	44.6 (32.1-56.6)	17.8 (9.2-29.0)
North Korea	25.2 (14.2-37.9)	56.8 (30.7-79.8)	46.9 (18.6-76.3)	24.8 (4.5-56.2)
Taiwan	20.8 (14.0-28.7)	67.6 (52.4-80.8)	66.0 (48.2-80.8)	46.2 (24.4-67.8)
Southeast Asia				
Brunei Darussalam	45.6 (36.4-55.0)	69.7 (55.4-82.0)	61.8 (46.1-75.9)	35.0 (18.9-53.2)
Cambodia	25.4 (15.2-37.5)	59.4 (39.9-77.9)	44.9 (24.0-66.0)	26.6 (8.5-50.9)
Indonesia	44.5 (36.8-52.6)	41.0 (29.3-52.8)	21.4 (12.9-31.1)	5.1 (2.1-9.8)
Lao PDR	31.4 (20.9-43.4)	49.3 (31.4-67.1)	35.6 (18.6-55.8)	15.5 (4.4-32.9)

Women aged 30-79 years	Hypertension (%)	Detection/ awareness (%)	Treatment (%)	Control (%)
Malaysia	41.0 (31.4-50.5)	52.3 (37.6-65.7)	45.5 (30.2-60.5)	19.1 (7.7-34.2)
Maldives	35.4 (24.0-48.2)	48.4 (28.5-68.5)	34.0 (15.6-55.4)	15.4 (3.5-35.4)
Myanmar	40.0 (29.8-50.9)	63.7 (48.5-77.5)	39.8 (24.4-55.9)	16.6 (6.4-31.8)
Philippines	32.8 (24.5-41.9)	59.1 (44.9-72.3)	42.2 (27.9-57.0)	20.8 (9.5-36.6)
Thailand	29.2 (18.8-41.0)	58.9 (42.9-74.3)	51.2 (31.5-70.5)	29.8 (10.7-54.2)
Timor-Leste	36.7 (25.5-49.1)	38.9 (22.8-57.1)	25.7 (12.2-42.4)	10.5 (2.9-23.1)
Viet Nam	26.4 (18.9-35.1)	52.9 (37.5-68.9)	34.2 (20.1-49.4)	15.0 (5.5-29.3)
**High-income Asia Pacific**				
Japan	22.5 (18.8-26.4)	68.8 (55.1-81.0)	51.2 (43.0-59.3)	30.3 (21.6-39.8)
Singapore	27.1 (21.7-32.9)	67.7 (57.2-77.6)	62.9 (51.7-73.3)	39.5 (26.1-53.9)
South Korea	21.2 (17.7-24.9)	77.5 (68.0-85.5)	77.5 (70.2-83.6)	57.3 (45.9-68.0)
**High-income western**				
High-income English-speaking countries				
Australia	26.3 (21.0-32.3)	61.3 (49.1-71.6)	49.5 (37.8-60.8)	27.0 (16.5-39.2)
Canada	19.9 (16.0-24.4)	75.1 (66.4-82.6)	70.6 (60.5-79.2)	56.9 (44.5-68.3)
Ireland	26.6 (17.3-37.7)	54.2 (36.5-71.7)	45.1 (27.5-63.8)	27.6 (10.4-50.1)
New Zealand	27.6 (21.4-34.4)	65.0 (53.9-75.3)	52.3 (40.6-63.8)	30.2 (18.1-44.1)
United Kingdom	23.0 (19.8-26.4)	58.4 (51.0-65.6)	47.9 (40.6-54.8)	29.2 (22.1-37.1)
United States of America	29.0 (23.6-34.7)	82.8 (75.6-88.7)	73.3 (64.0-81.5)	51.0 (38.2-63.8)
North Western Europe				
Austria	30.2 (19.3-42.8)	65.1 (40.6-85.0)	53.7 (31.0-76.5)	27.1 (7.7-57.0)
Belgium	26.1 (20.5-32.2)	73.4 (62.5-82.9)	65.0 (52.6-76.0)	42.3 (27.6-58.2)
Denmark	28.6 (21.7-36.0)	58.1 (43.7-72.1)	27.3 (17.5-38.4)	10.2 (4.3-19.1)
Finland	30.6 (24.1-37.4)	70.8 (59.7-80.6)	53.9 (40.4-67.0)	29.1 (16.2-43.7)
Germany	25.0 (17.0-34.2)	70.8 (57.0-83.4)	65.0 (48.6-79.8)	48.0 (25.3-70.7)
Greenland	28.4 (18.3-39.9)	59.0 (39.0-77.8)	46.3 (25.4-67.9)	29.3 (10.4-54.9)
Iceland	24.2 (16.7-32.8)	82.0 (69.4-91.2)	71.8 (55.3-85.6)	52.9 (30.3-75.2)
Luxembourg	24.2 (18.4-30.4)	59.6 (46.3-72.2)	51.3 (37.3-64.6)	33.7 (19.2-50.8)
Netherlands	24.8 (18.0-32.3)	55.0 (39.5-69.4)	46.5 (32.6-61.2)	26.5 (13.4-43.3)
Norway	25.5 (16.8-36.0)	66.4 (41.9-86.4)	45.6 (25.9-67.1)	28.7 (10.1-55.4)
Sweden	24.6 (18.5-31.4)	55.1 (42.4-67.1)	39.8 (27.1-53.0)	19.2 (8.7-33.1)
Switzerland	17.5 (11.9-24.2)	73.8 (61.0-84.7)	56.8 (40.7-72.0)	39.4 (21.6-59.5)
South Western Europe				
Andorra	26.2 (15.2-38.7)	68.5 (44.7-87.2)	58.8 (31.1-83.6)	37.5 (12.0-68.4)

Women aged 30-79 years	Hypertension (%)	Detection/ awareness (%)	Treatment (%)	Control (%)
Cyprus	26.0 (15.7-39.1)	68.4 (44.7-87.3)	58.7 (30.2-83.6)	37.6 (11.8-69.3)
France	24.4 (17.8-32.0)	68.3 (54.4-80.2)	55.7 (40.6-70.1)	34.6 (18.0-53.5)
Greece	26.2 (20.3-32.6)	70.3 (59.5-80.1)	67.1 (55.6-77.6)	39.7 (24.7-56.0)
Israel	25.3 (18.9-32.8)	66.3 (52.7-78.0)	56.6 (42.1-70.3)	31.0 (16.6-47.7)
Italy	28.6 (23.1-34.5)	65.3 (55.2-74.5)	58.0 (46.3-69.2)	32.9 (20.5-47.0)
Malta	24.8 (17.9-32.9)	73.8 (58.6-86.1)	68.6 (51.7-82.6)	48.0 (28.2-69.0)
Portugal	28.0 (20.7-36.2)	75.0 (62.1-86.1)	70.9 (55.8-83.7)	52.0 (31.8-70.9)
Spain	20.8 (15.7-26.5)	71.6 (60.6-80.7)	58.0 (45.1-70.6)	34.5 (20.0-50.5)
**Latin America and Caribbean**				
Andean Latin America				
Bolivia	27.2 (14.8-42.6)	70.7 (45.9-88.9)	59.8 (29.1-86.2)	33.7 (9.0-66.9)
Ecuador	25.1 (18.9-32.1)	74.5 (62.4-84.3)	63.0 (49.6-75.1)	40.0 (24.9-56.1)
Peru	18.4 (15.3-21.8)	60.0 (51.7-67.7)	53.7 (44.9-62.0)	31.1 (22.8-40.4)
Caribbean				
Antigua and Barbuda	43.2 (28.0-59.2)	71.8 (47.0-89.7)	57.7 (27.7-83.1)	27.4 (6.4-58.3)
Bahamas	43.6 (31.0-57.2)	73.3 (55.7-87.3)	62.9 (42.4-80.0)	27.5 (9.7-50.4)
Barbados	43.3 (31.9-56.1)	78.0 (61.7-90.0)	68.3 (48.1-84.5)	37.7 (16.0-63.1)
Belize	38.0 (24.5-53.2)	71.7 (50.8-88.0)	56.1 (31.6-78.1)	26.7 (7.2-54.7)
Bermuda	43.2 (28.3-58.9)	71.8 (47.1-90.2)	58.1 (29.7-84.0)	27.1 (6.1-58.5)
Cuba	39.5 (27.5-52.8)	76.9 (59.9-89.4)	68.6 (47.9-84.6)	38.0 (15.3-64.0)
Dominica	49.9 (35.4-64.9)	67.1 (45.9-84.9)	54.5 (31.1-76.1)	20.7 (5.0-46.0)
Dominican Republic	49.2 (36.5-62.4)	74.5 (58.4-86.8)	60.1 (40.3-78.0)	25.1 (8.0-48.9)
Grenada	45.6 (33.1-58.8)	73.3 (54.5-87.8)	59.7 (39.1-78.8)	23.8 (7.0-47.6)
Guyana	41.8 (31.8-51.9)	70.6 (55.1-83.2)	53.4 (35.9-70.2)	23.7 (10.0-41.7)
Haiti	47.8 (38.5-57.3)	66.0 (52.3-78.4)	33.1 (20.2-47.4)	8.6 (3.0-17.3)
Jamaica	47.8 (34.9-61.4)	76.8 (60.0-89.2)	63.7 (43.8-80.8)	23.6 (7.3-45.9)
Puerto Rico	43.2 (28.0-59.7)	71.7 (47.8-89.6)	58.0 (28.8-83.6)	27.0 (5.7-58.4)
Saint Kitts and Nevis	45.1 (30.7-59.7)	70.3 (49.9-86.3)	59.9 (35.7-80.4)	27.7 (7.0-55.7)
Saint Lucia	40.8 (29.5-53.0)	80.0 (64.3-91.5)	65.5 (44.4-82.3)	31.0 (11.6-55.2)
Saint Vincent and the Grenadines	41.5 (31.1-52.4)	73.0 (56.7-86.4)	56.7 (37.2-74.3)	25.4 (9.3-46.1)
Suriname	43.3 (31.2-56.3)	72.5 (55.0-86.5)	57.0 (35.8-76.7)	23.9 (7.8-45.7)
Trinidad and Tobago	41.6 (29.2-54.6)	67.6 (49.2-82.6)	53.9 (32.5-73.1)	24.5 (7.4-48.1)
Central Latin America				
Colombia	30.8 (21.8-40.6)	76.3 (62.4-87.4)	63.9 (46.6-79.3)	41.0 (20.5-64.0)

Women aged 30-79 years	Hypertension (%)	Detection/ awareness (%)	Treatment (%)	Control (%)
Costa Rica	39.4 (29.6-48.9)	81.7 (71.7-90.2)	76.1 (63.8-86.2)	53.5 (34.4-72.9)
El Salvador	33.6 (24.2-43.8)	81.6 (69.8-90.8)	71.0 (55.7-84.1)	48.3 (28.2-68.5)
Guatemala	32.6 (22.9-44.1)	62.7 (46.2-77.9)	40.4 (22.9-60.7)	22.7 (8.3-43.3)
Honduras	34.4 (22.2-48.5)	74.3 (54.9-89.5)	67.3 (43.2-86.8)	39.0 (13.5-69.1)
Mexico	31.4 (25.8-37.2)	67.5 (58.3-76.5)	59.7 (48.8-69.3)	33.7 (22.7-45.7)
Nicaragua	36.9 (23.3-52.1)	75.8 (55.1-90.5)	69.2 (44.9-88.6)	41.8 (15.1-71.5)
Panama	35.3 (23.8-48.2)	75.6 (58.1-89.2)	63.9 (41.8-82.7)	35.8 (13.9-62.0)
Venezuela	39.1 (30.5-48.5)	79.4 (67.5-89.0)	71.3 (57.5-83.2)	39.6 (22.4-58.2)
Southern Latin America				
Argentina	41.2 (33.7-49.3)	65.1 (52.9-76.1)	48.1 (35.0-60.5)	19.4 (10.1-31.6)
Brazil	42.1 (35.1-48.9)	73.4 (64.0-81.3)	69.8 (60.3-78.4)	38.9 (26.4-52.1)
Chile	33.1 (25.3-41.5)	79.7 (67.7-88.8)	68.2 (54.1-81.0)	41.8 (24.3-60.9)
Paraguay	50.9 (38.0-64.2)	67.9 (49.5-83.3)	48.9 (29.8-68.2)	17.9 (5.0-38.1)
Uruguay	38.9 (29.3-49.5)	72.7 (57.6-84.4)	62.6 (46.5-76.7)	33.4 (16.1-54.2)
**Oceania**				
Melanesia				
Fiji	40.5 (27.8-54.1)	60.9 (41.5-78.6)	40.9 (20.9-62.7)	15.6 (3.3-36.1)
Papua New Guinea	30.1 (17.2-45.5)	36.8 (17.3-60.3)	20.6 (6.0-42.4)	11.3 (1.6-31.8)
Solomon Islands	34.5 (24.8-45.1)	42.3 (25.8-60.5)	16.8 (7.0-30.9)	7.4 (1.7-18.3)
Vanuatu	41.8 (28.5-55.6)	35.8 (18.3-57.1)	16.0 (5.4-33.0)	6.1 (0.8-19.1)
Polynesia and Micronesia				
American Samoa	45.5 (31.2-59.6)	50.6 (32.4-68.6)	34.3 (15.4-56.3)	13.6 (2.6-33.7)
Cook Islands	41.1 (29.9-52.6)	59.4 (41.8-75.4)	44.6 (27.2-62.5)	18.3 (6.1-36.3)
Federated States of Micronesia	33.9 (24.1-44.4)	56.2 (39.8-71.9)	31.5 (17.2-48.6)	15.2 (5.2-31.2)
French Polynesia	41.5 (28.7-55.3)	46.7 (27.3-67.4)	32.0 (15.1-52.6)	12.8 (3.0-30.4)
Kiribati	43.8 (33.1-55.2)	35.8 (20.8-51.7)	15.0 (6.6-26.9)	7.7 (2.1-17.6)
Marshall Islands	32.9 (25.2-41.4)	49.1 (34.9-63.0)	34.0 (20.8-49.2)	17.0 (7.4-29.8)
Nauru	39.7 (29.4-50.7)	57.4 (41.3-72.7)	30.0 (15.3-47.4)	17.2 (5.9-34.1)
Niue	39.7 (27.3-53.1)	58.4 (39.5-76.4)	45.8 (26.0-66.2)	22.7 (7.0-45.8)
Palau	42.5 (33.0-51.9)	61.0 (45.8-75.2)	44.9 (30.4-60.3)	19.5 (8.5-35.2)
Samoa	37.9 (27.0-49.5)	40.2 (24.2-56.9)	20.6 (9.1-36.3)	10.0 (2.5-22.7)
Tokelau	37.5 (26.8-49.3)	53.8 (35.3-71.6)	42.8 (25.2-62.0)	17.8 (5.9-35.8)
Tonga	46.8 (37.9-55.9)	40.8 (28.0-54.5)	29.4 (18.1-42.0)	11.6 (4.8-21.2)
Tuvalu	50.8 (39.2-62.3)	35.8 (20.6-52.3)	20.9 (9.7-36.0)	6.3 (1.4-14.9)

Women aged 30-79 years	**Hypertension (%)**	**Detection/ awareness (%)**	**Treatment (%)**	**Control (%)**
**South Asia**				
Afghanistan	45.3 (35.5-55.1)	66.0 (52.0-78.5)	54.3 (39.1-69.4)	17.3 (7.9-30.2)
Bangladesh	34.2 (28.2-40.7)	53.5 (43.3-63.1)	41.5 (32.0-51.5)	15.1 (8.7-22.9)
Bhutan	43.0 (35.6-50.7)	52.4 (40.2-64.3)	31.4 (20.9-42.7)	10.7 (5.2-18.3)
India	30.5 (23.5-37.6)	41.7 (30.8-53.5)	35.1 (25.0-46.9)	18.5 (10.0-29.6)
Nepal	33.9 (28.4-39.8)	35.5 (26.5-45.1)	21.2 (15.0-28.3)	8.5 (4.7-13.4)
Pakistan	44.8 (32.4-57.7)	54.3 (37.8-70.1)	44.1 (26.3-62.5)	14.1 (4.2-29.6)
Sri Lanka	36.5 (26.7-47.5)	53.1 (36.1-68.8)	41.6 (25.9-57.9)	17.6 (6.6-33.1)
**Sub-Saharan Africa**				
Central Africa				
Angola	40.8 (27.2-56.1)	55.2 (33.6-76.2)	26.9 (10.0-49.8)	13.4 (2.8-33.2)
Central African Republic	42.8 (32.3-53.6)	40.1 (25.4-55.9)	20.9 (9.6-36.1)	6.2 (1.5-14.7)
Congo	41.5 (25.5-58.3)	45.2 (22.7-68.8)	25.9 (8.1-51.1)	11.2 (1.4-31.8)
DR Congo	35.7 (21.5-52.3)	43.9 (23.2-66.0)	26.5 (9.0-51.1)	12.4 (1.9-33.4)
Equatorial Guinea	39.9 (23.5-58.3)	48.5 (23.9-73.4)	30.1 (8.3-61.0)	13.3 (1.7-36.4)
Gabon	38.7 (24.4-54.3)	52.0 (30.2-72.5)	35.2 (15.0-59.8)	15.6 (2.9-37.6)
East Africa				
Burundi	35.6 (21.6-51.5)	47.5 (24.1-71.7)	29.3 (8.5-56.7)	13.3 (2.0-35.6)
Comoros	36.2 (24.7-49.2)	49.7 (31.4-68.6)	29.7 (13.6-49.0)	12.9 (3.1-29.3)
Djibouti	35.8 (21.4-51.6)	47.5 (23.4-71.9)	28.9 (8.5-55.9)	13.3 (2.1-35.0)
Eritrea	24.7 (14.8-36.6)	42.9 (24.9-62.7)	26.7 (11.8-45.8)	13.6 (3.2-31.2)
Ethiopia	30.1 (20.8-40.6)	37.4 (22.5-53.1)	15.6 (7.1-27.7)	6.6 (1.8-15.2)
Kenya	34.7 (25.8-44.3)	49.2 (33.1-65.5)	20.5 (10.0-33.9)	7.7 (2.2-16.6)
Madagascar	38.9 (24.9-54.2)	40.3 (21.3-61.7)	18.6 (6.0-37.2)	7.1 (1.0-21.3)
Malawi	31.6 (24.2-39.4)	45.3 (32.4-58.3)	28.9 (18.2-41.4)	13.9 (6.1-24.9)
Mauritius	32.0 (24.4-39.5)	72.2 (60.7-82.5)	66.2 (53.1-78.0)	38.7 (22.7-57.0)
Mozambique	41.9 (31.4-53.0)	35.7 (21.5-51.8)	18.9 (8.6-33.0)	9.2 (2.7-20.6)
Rwanda	30.9 (20.1-42.8)	28.6 (13.7-46.5)	11.4 (3.5-24.0)	5.8 (1.1-16.4)
Seychelles	42.3 (32.3-52.7)	74.3 (60.8-85.1)	64.5 (47.4-80.1)	35.8 (16.8-58.3)
Somalia	38.6 (26.5-51.8)	44.8 (26.0-64.7)	29.4 (13.2-49.7)	10.2 (2.1-24.4)
South Sudan	36.0 (22.2-51.5)	47.4 (23.8-71.8)	29.2 (8.8-56.8)	13.2 (1.9-35.6)
Sudan	43.5 (33.1-54.0)	39.8 (25.3-55.5)	28.2 (16.4-43.0)	9.7 (3.3-19.9)
Tanzania	35.4 (25.9-45.5)	40.4 (26.6-55.8)	16.6 (8.0-28.0)	8.0 (2.5-17.2)
Uganda	33.9 (23.6-45.3)	41.5 (26.2-58.3)	22.7 (11.0-38.0)	8.6 (2.4-19.5)

Women aged 30-79 years	Hypertension (%)	Detection/ awareness (%)	Treatment (%)	Control (%)
Zambia	33.8 (25.6-42.2)	52.1 (37.7-66.3)	28.7 (16.7-42.6)	11.9 (4.5-23.3)
Southern Africa				
Botswana	47.0 (35.0-59.4)	65.0 (48.4-80.0)	47.5 (30.2-65.3)	22.4 (8.8-41.6)
Eswatini	47.3 (35.8-58.8)	58.8 (42.0-75.1)	39.9 (23.5-57.3)	14.9 (4.6-30.7)
Lesotho	46.8 (35.8-57.7)	60.8 (43.5-76.4)	51.1 (34.5-67.8)	20.8 (7.9-38.9)
Namibia	44.8 (32.3-57.9)	57.3 (39.0-75.2)	48.1 (30.1-66.1)	24.5 (9.2-45.6)
South Africa	44.3 (36.8-51.7)	62.8 (51.8-73.2)	54.7 (43.7-65.5)	29.9 (18.4-42.6)
Zimbabwe	46.4 (30.6-63.1)	53.5 (27.6-78.1)	40.6 (14.1-70.2)	18.9 (3.3-47.4)
West Africa				
Benin	33.0 (26.1-40.4)	46.6 (33.5-59.9)	29.2 (18.8-41.5)	10.2 (4.4-18.7)
Burkina Faso	31.7 (20.9-43.2)	44.1 (26.6-62.7)	26.1 (12.3-43.2)	11.2 (3.0-25.6)
Cabo Verde	41.7 (34.4-49.2)	64.5 (51.7-76.0)	47.1 (34.0-60.4)	19.4 (9.9-31.6)
Cameroon	38.6 (27.7-50.6)	42.5 (26.9-59.2)	21.9 (9.9-37.1)	10.3 (2.7-23.3)
Chad	40.7 (26.8-56.1)	45.9 (24.6-69.3)	27.9 (10.1-52.3)	12.8 (2.1-32.6)
Cote d'Ivoire	37.6 (23.6-53.1)	44.5 (24.9-65.6)	26.6 (10.5-48.0)	11.0 (1.9-29.2)
Gambia	40.2 (27.5-53.8)	53.1 (34.1-71.8)	32.6 (16.1-52.6)	10.9 (2.4-26.1)
Ghana	36.1 (26.6-46.3)	60.0 (45.5-73.7)	44.6 (30.1-60.2)	23.7 (10.7-40.7)
Guinea	42.5 (29.3-56.7)	53.2 (33.1-72.8)	26.5 (10.4-47.4)	8.2 (1.3-23.1)
Guinea Bissau	39.7 (25.0-56.2)	50.7 (26.1-75.9)	30.8 (9.6-57.9)	13.2 (1.9-36.0)
Liberia	41.3 (28.8-54.5)	47.0 (28.4-66.0)	28.6 (13.4-47.5)	8.8 (1.8-22.2)
Mali	38.3 (24.8-53.1)	59.7 (39.5-78.6)	39.7 (19.0-63.0)	16.4 (3.7-37.4)
Mauritania	39.7 (24.6-56.6)	50.9 (25.8-75.7)	30.5 (9.9-57.4)	13.1 (2.0-34.7)
Niger	42.6 (28.5-57.4)	33.3 (16.3-54.6)	14.9 (4.8-31.4)	8.0 (1.2-22.6)
Nigeria	39.0 (31.5-47.0)	50.6 (37.9-63.3)	29.6 (19.1-41.8)	10.8 (4.9-19.0)
Sao Tome and Principe	48.0 (39.8-56.6)	61.1 (48.1-73.4)	36.1 (23.8-49.5)	11.4 (5.0-20.7)
Senegal	42.9 (30.1-56.3)	44.5 (25.0-64.8)	25.0 (9.3-45.6)	11.1 (2.3-27.6)
Sierra Leone	43.4 (30.1-57.3)	44.6 (25.6-64.3)	24.2 (10.1-43.1)	10.5 (2.1-26.2)
Togo	37.5 (25.2-51.0)	52.3 (32.8-71.9)	26.1 (11.4-45.1)	13.0 (3.0-30.9)

Men aged 30-79 years	**Hypertension (%)**	**Detection/ awareness (%)**	**Treatment (%)**	**Control (%)**
**Central and Eastern Europe**				
Central Europe				
Albania	42.6 (33.8-51.7)	28.5 (17.9-40.7)	19.3 (10.7-30.2)	5.3 (1.9-10.8)
Bosnia and Herzegovina	46.9 (34.4-59.8)	48.2 (32.3-64.3)	42.0 (25.4-59.4)	11.7 (3.0-26.5)
Bulgaria	49.4 (33.0-66.0)	56.8 (32.3-79.6)	45.2 (20.0-71.8)	18.4 (3.7-43.8)
Croatia	51.4 (40.3-62.9)	70.3 (54.3-83.8)	46.2 (30.6-61.8)	14.6 (5.1-29.5)
Czechia	49.1 (41.4-56.9)	69.5 (59.3-79.1)	59.1 (47.8-69.9)	30.7 (18.8-44.4)
Hungary	55.9 (41.2-70.7)	51.9 (32.7-70.7)	46.0 (25.9-66.6)	18.2 (4.9-40.1)
Montenegro	49.5 (33.7-66.2)	56.9 (33.3-78.9)	45.0 (20.6-71.2)	18.2 (3.6-42.8)
North Macedonia	49.4 (33.6-65.5)	57.0 (32.5-78.6)	45.2 (19.6-71.8)	18.3 (3.5-43.4)
Poland	55.5 (46.9-64.2)	64.8 (54.4-74.7)	56.2 (44.7-67.5)	28.3 (16.9-41.5)
Romania	52.6 (43.1-62.4)	61.9 (48.8-74.3)	53.3 (39.4-66.7)	23.2 (11.9-38.0)
Serbia	49.9 (38.3-61.3)	58.6 (44.1-72.2)	49.7 (34.0-65.2)	19.5 (7.6-36.3)
Slovakia	47.4 (35.8-59.0)	67.6 (51.5-81.8)	58.5 (40.6-74.6)	30.0 (12.8-51.7)
Slovenia	49.6 (33.2-66.2)	57.0 (32.7-79.2)	45.4 (21.2-71.8)	18.3 (3.6-43.2)
Eastern Europe				
Belarus	51.6 (41.9-61.1)	60.6 (46.5-73.4)	37.6 (24.1-51.6)	6.6 (2.3-13.3)
Estonia	45.9 (32.9-59.2)	47.1 (30.6-63.7)	35.9 (19.4-54.1)	13.0 (3.4-28.9)
Latvia	48.9 (34.5-63.0)	63.1 (44.4-79.9)	44.5 (25.7-63.9)	14.8 (4.0-32.7)
Lithuania	54.0 (39.5-67.8)	64.7 (47.2-80.2)	37.3 (20.2-56.6)	9.9 (2.0-25.3)
Moldova	49.3 (37.1-61.5)	53.8 (37.2-69.2)	31.2 (17.3-47.8)	7.4 (1.9-17.2)
Russian Federation	47.3 (38.2-56.3)	67.0 (56.1-77.1)	42.6 (30.6-54.8)	14.1 (6.6-24.5)
Ukraine	44.5 (36.2-53.1)	54.0 (41.4-66.4)	36.0 (24.5-48.8)	10.1 (4.5-18.2)
**Central Asia, Middle East and North Africa**				
Central Asia				
Armenia	48.5 (38.0-58.9)	35.6 (22.7-48.8)	22.5 (11.9-35.0)	7.3 (2.3-15.6)
Azerbaijan	39.6 (30.6-48.8)	46.5 (33.6-59.6)	32.5 (20.5-45.9)	8.8 (3.5-17.2)
Georgia	46.5 (36.6-56.3)	60.1 (47.4-72.0)	41.0 (27.9-54.5)	13.1 (5.5-24.3)
Kazakhstan	40.9 (32.4-49.6)	80.3 (71.3-87.7)	65.7 (53.6-76.9)	25.4 (14.4-39.0)
Kyrgyzstan	38.1 (28.2-48.6)	45.8 (31.0-60.3)	30.8 (17.7-45.8)	8.7 (2.5-19.4)
Mongolia	45.0 (37.3-52.7)	63.9 (54.1-73.1)	45.7 (34.7-56.9)	18.7 (10.7-29.0)
Tajikistan	50.9 (40.8-61.6)	39.9 (26.6-53.9)	23.3 (12.6-36.6)	5.3 (1.5-12.0)
Turkmenistan	37.6 (30.1-45.6)	52.8 (41.4-63.9)	38.2 (26.8-50.8)	8.7 (3.7-16.0)
Uzbekistan	46.7 (38.6-55.1)	44.0 (33.4-54.9)	33.6 (23.3-44.8)	11.3 (5.8-18.8)

Men aged 30-79 years	Hypertension (%)	Detection/ awareness (%)	Treatment (%)	Control (%)
Middle East and North Africa				
Algeria	35.0 (26.6-44.2)	40.3 (28.6-52.9)	30.1 (19.2-42.0)	11.1 (4.6-20.5)
Bahrain	40.1 (26.4-55.2)	47.0 (24.9-69.6)	37.6 (16.0-62.6)	16.4 (3.4-39.5)
Egypt	35.6 (28.3-43.4)	43.8 (32.9-54.5)	34.6 (24.7-45.3)	14.2 (7.4-23.6)
Iran	26.6 (22.7-30.7)	49.0 (42.3-55.9)	37.6 (30.7-44.5)	18.6 (13.2-24.8)
Iraq	48.4 (37.7-59.4)	55.9 (41.2-69.9)	38.7 (24.7-54.3)	11.3 (4.2-22.2)
Jordan	39.6 (33.1-46.5)	58.8 (49.6-67.9)	51.5 (41.7-61.2)	28.7 (19.3-39.3)
Kuwait	43.5 (33.9-53.2)	57.9 (46.0-69.7)	51.2 (38.7-63.7)	27.2 (14.3-43.1)
Lebanon	42.1 (33.1-51.6)	48.9 (36.7-61.2)	43.6 (31.2-56.8)	23.0 (12.0-36.4)
Libya	45.9 (33.1-59.0)	39.5 (24.0-56.1)	28.1 (14.3-44.4)	7.6 (1.6-19.6)
Morocco	35.0 (26.5-44.3)	33.8 (21.7-47.2)	20.3 (11.2-31.5)	6.4 (2.2-13.0)
Occupied Palestinian Territory	40.7 (29.3-52.8)	49.6 (34.0-66.5)	47.1 (30.1-64.7)	17.7 (6.2-35.1)
Oman	48.3 (38.9-57.8)	35.9 (25.3-47.4)	30.4 (20.3-41.9)	11.3 (5.3-19.7)
Qatar	41.6 (30.5-53.0)	54.6 (39.6-69.1)	47.8 (31.6-63.6)	21.2 (8.2-39.1)
Saudi Arabia	36.3 (26.6-46.7)	46.8 (30.4-63.3)	37.0 (21.1-54.3)	19.0 (7.8-34.5)
Syrian Arab Republic	42.4 (29.0-56.5)	47.1 (25.2-70.1)	37.7 (15.9-63.7)	16.6 (3.5-39.3)
Tunisia	34.5 (24.7-45.5)	36.4 (23.9-50.5)	29.6 (17.6-43.4)	10.3 (3.4-21.2)
Turkey	30.8 (24.2-38.3)	53.1 (41.4-63.7)	50.6 (39.3-61.3)	28.2 (16.8-41.6)
United Arab Emirates	43.9 (35.3-52.9)	40.5 (29.3-52.2)	35.0 (24.3-46.5)	18.3 (9.9-29.2)
Yemen	29.1 (18.5-41.4)	40.6 (24.1-58.4)	33.6 (18.1-51.6)	13.1 (3.3-30.3)
**East and Southeast Asia**				
East Asia				
China	30.2 (23.4-37.2)	47.7 (37.4-57.8)	35.1 (25.1-45.1)	13.9 (7.3-22.7)
North Korea	28.1 (16.9-40.8)	50.4 (25.5-74.8)	39.3 (14.9-67.4)	20.4 (3.9-48.7)
Taiwan	27.3 (18.9-37.2)	61.7 (47.9-74.2)	56.6 (41.4-70.7)	37.1 (20.4-55.7)
Southeast Asia				
Brunei Darussalam	47.0 (36.8-57.5)	61.8 (48.6-74.0)	52.1 (38.7-65.5)	25.6 (13.2-40.5)
Cambodia	25.8 (15.3-37.7)	40.5 (24.2-58.1)	27.0 (12.5-44.5)	16.4 (4.7-35.1)
Indonesia	35.9 (28.5-43.8)	29.0 (20.3-38.7)	15.4 (9.1-23.1)	3.6 (1.5-6.9)
Lao PDR	25.6 (16.3-36.3)	40.2 (24.7-57.4)	27.3 (13.6-44.1)	12.8 (3.6-28.0)
Malaysia	40.5 (30.4-51.0)	46.6 (33.4-59.3)	40.3 (26.9-54.2)	19.6 (9.3-33.4)
Maldives	32.6 (20.7-45.9)	39.4 (22.7-57.8)	27.7 (12.9-46.2)	14.0 (3.3-31.9)
Myanmar	35.2 (25.4-45.5)	49.2 (35.9-62.2)	27.1 (15.6-39.8)	12.8 (4.8-25.0)
Philippines	34.7 (25.7-44.6)	45.7 (33.3-58.2)	28.7 (18.2-41.1)	13.4 (6.0-24.2)

Men aged 30-79 years	**Hypertension (%)**	**Detection/ awareness (%)**	**Treatment (%)**	**Control (%)**
Thailand	29.1 (18.4-41.3)	47.1 (31.9-62.5)	37.0 (20.9-54.2)	22.0 (7.5-41.8)
Timor-Leste	33.8 (23.2-45.4)	35.9 (21.6-51.4)	22.6 (10.9-36.9)	11.9 (3.6-25.0)
Viet Nam	32.9 (23.9-42.5)	42.3 (30.4-55.3)	26.1 (15.8-38.0)	10.7 (4.1-21.1)
**High-income Asia Pacific**				
Japan	40.3 (35.0-45.8)	65.8 (51.3-79.2)	45.9 (38.8-53.1)	24.1 (17.3-31.8)
Singapore	35.4 (28.9-42.3)	64.9 (55.1-74.3)	58.9 (48.8-68.5)	36.2 (24.6-49.5)
South Korea	32.0 (27.1-37.4)	67.2 (59.0-74.8)	67.2 (60.5-73.3)	49.8 (40.2-59.2)
**High-income western**				
High-income English-speaking countries				
Australia	32.3 (26.2-39.0)	57.7 (47.2-67.1)	46.7 (36.5-57.0)	24.9 (15.6-35.8)
Canada	24.3 (20.0-29.0)	80.1 (73.1-86.2)	75.8 (67.8-82.4)	64.0 (53.6-73.8)
Ireland	38.2 (26.5-50.6)	47.5 (31.8-64.4)	37.6 (22.3-54.6)	21.1 (7.7-40.8)
New Zealand	34.4 (27.4-42.0)	61.2 (51.2-70.8)	47.0 (36.5-57.4)	24.9 (15.2-36.2)
United Kingdom	29.9 (25.9-34.0)	59.5 (53.0-65.7)	47.4 (41.1-53.7)	30.6 (24.1-37.7)
United States of America	34.1 (28.0-40.4)	78.4 (70.8-85.0)	66.3 (56.8-74.7)	44.8 (33.2-56.9)
North Western Europe				
Austria	37.5 (24.7-51.5)	61.7 (37.8-82.6)	54.3 (33.0-75.2)	27.5 (8.9-54.1)
Belgium	33.8 (26.5-41.4)	62.0 (50.3-72.7)	55.2 (44.2-66.0)	34.7 (22.8-47.9)
Denmark	43.3 (34.3-52.2)	54.1 (41.4-66.8)	25.1 (17.5-33.8)	8.8 (4.0-15.8)
Finland	41.0 (33.0-49.5)	65.9 (55.6-75.4)	48.8 (38.4-58.8)	27.0 (15.9-39.2)
Germany	34.4 (24.5-45.0)	72.2 (58.8-83.9)	61.1 (46.5-74.5)	43.2 (23.1-63.8)
Greenland	37.9 (25.2-51.2)	50.3 (33.0-68.0)	38.4 (21.9-56.7)	19.7 (5.9-40.8)
Iceland	30.9 (22.3-40.6)	82.8 (72.2-91.1)	71.0 (56.8-82.8)	50.9 (29.3-71.4)
Luxembourg	36.6 (29.0-44.4)	58.8 (47.3-70.0)	51.1 (38.9-63.1)	26.8 (15.4-40.5)
Netherlands	36.2 (27.4-44.8)	48.8 (35.4-62.6)	39.6 (28.7-51.6)	20.5 (9.9-35.4)
Norway	35.3 (24.4-47.3)	62.4 (39.6-82.6)	47.8 (30.3-66.3)	28.8 (10.1-52.5)
Sweden	35.6 (27.1-44.1)	53.1 (40.8-65.5)	39.6 (28.2-52.2)	20.5 (9.9-33.9)
Switzerland	26.4 (19.3-34.5)	72.7 (61.4-82.9)	55.8 (42.3-68.9)	35.2 (19.3-52.9)
South Western Europe				
Andorra	35.7 (22.0-50.7)	61.9 (37.1-82.2)	52.3 (27.8-75.7)	29.8 (8.7-59.1)
Cyprus	35.7 (22.5-50.7)	61.8 (38.8-81.8)	52.1 (27.2-75.9)	30.0 (8.6-57.7)
France	34.1 (26.7-42.4)	62.2 (49.9-73.4)	49.9 (36.9-63.1)	23.4 (11.4-39.3)
Greece	36.5 (29.1-44.5)	59.3 (49.2-68.9)	55.4 (45.0-65.4)	28.4 (17.1-41.3)
Israel	33.0 (25.2-41.8)	60.5 (47.9-72.1)	49.6 (36.8-61.9)	24.1 (12.4-38.4)

Men aged 30-79 years	Hypertension (%)	Detection/ awareness (%)	Treatment (%)	Control (%)
Italy	39.1 (32.0-46.3)	59.3 (49.2-68.6)	51.0 (41.5-60.4)	24.6 (14.8-36.0)
Malta	34.1 (25.4-43.5)	69.5 (55.3-81.3)	65.1 (50.2-78.3)	42.9 (24.5-61.6)
Portugal	37.0 (28.0-46.8)	64.1 (50.3-76.6)	57.2 (42.1-71.4)	38.4 (21.5-57.4)
Spain	33.5 (26.2-41.4)	61.4 (50.9-71.4)	51.0 (40.3-61.9)	29.5 (17.5-43.6)
**Latin America and Caribbean**				
Andean Latin America				
Bolivia	29.4 (15.3-47.1)	51.0 (26.9-75.0)	39.3 (15.6-67.2)	19.3 (3.9-45.2)
Ecuador	29.2 (22.0-37.2)	50.7 (39.0-61.8)	37.2 (26.0-49.1)	18.2 (9.6-29.1)
Peru	22.8 (19.0-27.1)	35.5 (29.0-42.0)	28.9 (23.1-35.0)	14.1 (9.5-19.4)
Caribbean				
Antigua and Barbuda	41.9 (25.9-58.6)	53.3 (29.7-76.3)	39.7 (16.0-66.6)	18.1 (3.5-42.9)
Bahamas	45.5 (32.7-58.6)	57.2 (40.3-73.6)	42.3 (24.8-60.4)	16.9 (5.1-34.7)
Barbados	40.0 (28.7-52.7)	61.7 (44.5-77.3)	51.0 (33.0-69.3)	28.9 (11.6-50.9)
Belize	38.0 (24.1-53.7)	47.1 (28.3-66.9)	34.0 (16.2-55.5)	15.9 (3.4-35.3)
Bermuda	42.0 (26.0-59.6)	53.3 (29.3-76.6)	40.0 (16.9-68.0)	17.8 (3.4-41.8)
Cuba	40.3 (27.6-53.9)	63.3 (45.6-78.5)	52.5 (33.0-71.3)	27.6 (10.4-50.5)
Dominica	45.5 (31.1-60.3)	47.7 (29.0-67.1)	36.2 (17.8-57.2)	14.4 (3.2-33.6)
Dominican Republic	49.0 (35.5-62.2)	60.6 (43.8-75.9)	46.4 (28.2-64.7)	17.9 (5.5-36.5)
Grenada	47.6 (33.9-61.5)	53.0 (35.3-70.4)	35.3 (18.6-53.8)	14.7 (4.0-32.1)
Guyana	38.2 (28.3-48.7)	54.8 (39.2-69.5)	40.1 (24.7-56.5)	16.4 (6.7-29.6)
Haiti	37.6 (28.8-46.8)	43.0 (29.7-56.8)	21.4 (12.2-32.9)	7.5 (2.8-14.7)
Jamaica	44.5 (31.8-58.0)	51.0 (34.1-67.6)	37.3 (20.9-55.1)	14.0 (4.0-29.6)
Puerto Rico	41.8 (25.5-59.6)	53.4 (29.9-76.6)	39.6 (16.4-66.3)	17.8 (3.4-41.7)
Saint Kitts and Nevis	45.1 (29.9-60.6)	50.3 (31.6-69.4)	37.8 (18.9-59.1)	16.8 (3.9-38.1)
Saint Lucia	38.8 (26.4-51.9)	57.3 (40.3-73.7)	37.5 (20.8-56.2)	12.1 (3.1-26.5)
Saint Vincent and the Grenadines	37.2 (26.3-48.5)	49.1 (33.6-64.2)	33.3 (19.2-49.2)	15.3 (5.4-29.8)
Suriname	42.4 (29.7-55.8)	54.6 (37.9-70.7)	41.6 (23.7-60.5)	18.1 (5.7-35.2)
Trinidad and Tobago	43.2 (30.8-56.6)	52.3 (35.5-68.7)	39.5 (23.0-57.1)	17.3 (5.3-35.8)
Central Latin America				
Colombia	31.1 (21.4-41.9)	60.3 (46.3-73.4)	45.6 (30.9-61.6)	24.0 (9.9-43.1)
Costa Rica	36.0 (26.9-45.6)	71.8 (60.2-82.6)	63.5 (50.7-75.7)	45.4 (28.3-63.2)
El Salvador	31.4 (22.2-41.8)	61.2 (46.6-74.8)	50.6 (34.6-66.3)	26.6 (12.5-44.1)
Guatemala	31.5 (21.0-43.1)	48.5 (32.8-64.1)	30.3 (16.0-47.5)	14.7 (4.7-31.1)
Honduras	33.2 (20.1-48.2)	56.1 (34.3-76.9)	47.9 (24.6-70.9)	25.4 (7.2-52.1)

Men aged 30-79 years	**Hypertension (%)**	**Detection/ awareness (%)**	**Treatment (%)**	**Control (%)**
Mexico	32.8 (26.4-39.2)	46.9 (37.8-56.2)	39.3 (30.3-48.8)	21.2 (13.5-30.5)
Nicaragua	34.5 (21.1-50.6)	56.3 (33.8-77.1)	49.7 (25.4-73.5)	25.8 (7.2-52.2)
Panama	36.8 (23.8-51.3)	59.0 (41.5-75.7)	45.7 (26.7-66.2)	21.4 (6.8-43.4)
Venezuela	39.7 (30.1-50.0)	64.8 (51.5-77.5)	54.2 (40.5-67.8)	25.3 (12.9-41.4)
Southern Latin America				
Argentina	54.0 (45.1-62.9)	52.5 (41.0-63.6)	35.3 (24.9-46.4)	11.0 (5.4-18.7)
Brazil	47.9 (40.2-55.6)	61.8 (52.4-70.8)	54.4 (44.9-63.8)	28.1 (18.5-39.2)
Chile	39.0 (29.8-48.2)	63.7 (51.3-75.4)	49.9 (36.8-62.8)	26.7 (14.4-42.6)
Paraguay	61.6 (47.8-74.4)	44.0 (28.2-61.1)	28.4 (15.3-44.1)	8.4 (2.0-20.0)
Uruguay	46.0 (35.0-57.4)	61.0 (46.6-74.5)	47.3 (33.0-61.6)	24.9 (11.2-42.1)
**Oceania**				
Melanesia				
Fiji	36.5 (24.1-50.5)	45.3 (27.9-62.8)	27.7 (12.6-45.7)	9.1 (1.7-23.2)
Papua New Guinea	25.4 (14.0-40.3)	27.7 (11.8-47.4)	17.5 (5.4-35.5)	8.6 (1.2-24.6)
Solomon Islands	24.9 (16.4-35.0)	24.7 (13.3-38.0)	11.2 (4.2-21.5)	5.2 (1.1-13.2)
Vanuatu	37.4 (25.1-51.5)	23.9 (11.0-40.3)	11.0 (3.5-22.6)	3.6 (0.5-11.0)
Polynesia and Micronesia				
American Samoa	48.8 (33.5-63.9)	44.8 (26.2-63.1)	27.6 (11.5-46.7)	8.8 (1.5-23.8)
Cook Islands	44.6 (32.7-56.6)	54.0 (38.5-69.3)	38.7 (24.0-54.6)	14.9 (5.3-29.7)
Federated States of Micronesia	31.7 (21.9-42.0)	43.9 (30.1-58.1)	23.7 (12.3-37.4)	11.7 (3.8-24.8)
French Polynesia	43.7 (30.4-57.4)	39.2 (23.3-56.9)	29.0 (14.5-46.0)	11.6 (2.9-26.2)
Kiribati	40.4 (29.7-52.3)	31.0 (18.4-45.1)	15.6 (7.0-27.1)	5.3 (1.3-12.7)
Marshall Islands	30.8 (22.4-39.6)	41.9 (29.3-55.1)	26.5 (15.6-38.9)	12.3 (5.4-22.0)
Nauru	43.7 (32.5-55.6)	49.0 (33.2-64.2)	27.6 (15.0-42.5)	12.8 (4.3-26.4)
Niue	39.1 (26.5-52.8)	49.7 (32.3-68.5)	37.6 (20.7-56.6)	14.4 (3.9-31.8)
Palau	45.1 (35.2-55.4)	48.5 (34.6-62.2)	29.2 (18.4-41.9)	9.5 (3.7-18.9)
Samoa	38.6 (27.8-50.3)	30.2 (17.8-44.5)	20.3 (9.7-34.4)	9.9 (2.9-22.1)
Tokelau	41.3 (29.2-54.5)	41.8 (26.3-58.8)	24.3 (11.7-39.8)	7.5 (1.8-18.7)
Tonga	39.6 (30.6-49.4)	32.1 (20.9-44.1)	22.5 (13.0-33.7)	6.8 (2.6-13.5)
Tuvalu	49.2 (36.9-61.8)	29.8 (17.1-45.3)	18.9 (9.0-32.0)	5.8 (1.4-13.9)
**South Asia**				
Afghanistan	35.3 (26.5-45.0)	43.9 (30.7-57.6)	34.6 (21.7-49.0)	9.9 (4.1-18.3)
Bangladesh	23.5 (17.9-29.8)	44.8 (34.8-54.9)	34.1 (24.5-44.2)	14.0 (7.7-21.9)
Bhutan	43.6 (35.7-51.9)	40.7 (30.4-51.5)	21.1 (13.5-29.9)	6.9 (3.3-12.3)

Men aged 30-79 years	Hypertension (%)	Detection/ awareness (%)	Treatment (%)	Control (%)
India	31.6 (25.1-38.7)	31.7 (23.4-40.6)	25.1 (17.3-33.8)	11.3 (6.0-18.4)
Nepal	39.6 (33.6-45.9)	30.3 (22.8-38.6)	15.9 (11.0-21.4)	5.9 (3.3-9.5)
Pakistan	41.6 (29.4-54.3)	34.0 (20.5-49.1)	24.9 (12.4-39.9)	8.4 (2.1-19.5)
Sri Lanka	34.4 (24.5-45.0)	38.7 (24.5-53.6)	28.6 (16.6-42.1)	13.0 (4.7-25.1)
**Sub-Saharan Africa**				
Central Africa				
Angola	36.5 (22.4-51.7)	39.1 (20.3-60.8)	22.2 (8.5-41.2)	9.3 (1.7-24.4)
Central African Republic	39.5 (28.6-50.8)	25.0 (14.6-36.9)	14.1 (6.4-24.7)	4.9 (1.3-12.0)
Congo	38.0 (22.6-55.0)	30.9 (13.3-51.8)	21.6 (6.9-42.2)	7.6 (1.0-23.4)
DR Congo	32.7 (19.2-48.5)	34.7 (17.3-54.6)	25.4 (9.4-46.4)	10.7 (1.7-28.3)
Equatorial Guinea	36.6 (20.9-54.6)	34.2 (13.6-58.4)	22.6 (5.9-47.1)	9.6 (1.1-27.9)
Gabon	36.0 (22.0-51.2)	32.6 (17.1-50.9)	22.7 (8.7-41.4)	10.8 (2.0-27.7)
East Africa				
Burundi	32.5 (18.5-48.4)	32.7 (14.1-56.1)	20.1 (5.5-42.7)	9.2 (1.2-26.6)
Comoros	30.0 (19.1-42.1)	31.5 (17.6-47.9)	18.7 (7.8-33.7)	9.4 (2.3-22.5)
Djibouti	32.7 (18.6-49.0)	32.9 (13.8-56.0)	20.1 (5.3-42.3)	9.1 (1.2-26.4)
Eritrea	22.5 (12.9-33.9)	33.3 (18.5-50.0)	21.4 (9.3-37.7)	11.5 (3.0-25.8)
Ethiopia	24.5 (16.3-34.3)	30.1 (18.4-43.3)	15.6 (7.5-26.2)	5.6 (1.5-12.9)
Kenya	31.4 (22.6-41.0)	27.8 (16.8-40.3)	9.9 (4.1-18.4)	4.5 (1.2-10.4)
Madagascar	34.7 (21.1-49.9)	26.7 (12.8-44.8)	12.6 (3.8-26.7)	5.7 (0.8-16.6)
Malawi	26.8 (19.0-35.0)	26.7 (17.3-37.8)	16.6 (9.4-25.7)	6.4 (2.4-12.7)
Mauritius	34.2 (26.2-42.7)	61.2 (49.6-72.1)	54.4 (41.5-66.7)	30.5 (17.1-46.4)
Mozambique	34.3 (24.3-45.4)	22.2 (12.1-34.8)	10.1 (4.0-19.1)	4.9 (1.1-12.0)
Rwanda	28.5 (18.2-40.9)	17.7 (7.8-30.7)	9.7 (3.2-19.8)	5.9 (1.2-16.0)
Seychelles	45.8 (34.5-57.6)	58.6 (43.4-72.2)	47.1 (31.5-63.4)	18.5 (7.1-34.3)
Somalia	33.4 (21.0-47.9)	34.4 (17.9-53.8)	22.9 (9.3-40.4)	8.8 (1.8-22.0)
South Sudan	32.3 (18.0-48.6)	32.6 (13.0-56.6)	20.5 (5.7-43.2)	9.3 (1.3-26.1)
Sudan	38.0 (28.1-48.9)	24.8 (14.5-37.3)	16.3 (8.2-26.8)	6.4 (2.1-13.5)
Tanzania	30.9 (21.7-40.8)	25.5 (15.4-37.3)	13.7 (6.8-23.1)	6.0 (1.8-13.5)
Uganda	30.9 (20.8-41.9)	24.8 (14.1-37.7)	13.0 (5.5-23.0)	6.5 (1.7-15.5)
Zambia	30.3 (21.6-39.7)	34.1 (21.9-47.2)	17.8 (9.5-28.5)	6.6 (2.2-13.9)
Southern Africa				
Botswana	40.3 (29.1-52.2)	49.8 (34.4-65.0)	32.5 (18.9-47.9)	13.3 (4.4-27.0)
Eswatini	37.4 (26.2-49.1)	37.4 (23.8-53.0)	26.9 (14.7-41.7)	9.1 (2.6-19.8)

Men aged 30-79 years	Hypertension (%)	Detection/ awareness (%)	Treatment (%)	Control (%)
Lesotho	32.1 (22.5-42.5)	40.8 (27.0-55.3)	29.4 (16.6-43.8)	13.9 (4.9-27.4)
Namibia	42.6 (30.1-54.9)	45.6 (29.8-61.7)	38.9 (23.2-55.5)	17.7 (6.4-34.8)
South Africa	43.8 (36.6-51.2)	42.5 (33.5-51.8)	37.2 (28.2-46.5)	16.4 (9.7-24.2)
Zimbabwe	36.9 (22.0-53.3)	38.2 (16.0-62.4)	27.5 (8.3-52.9)	12.4 (1.9-32.9)
West Africa				
Benin	28.9 (22.1-36.1)	28.3 (19.6-38.2)	19.4 (12.1-28.1)	8.1 (3.8-14.4)
Burkina Faso	29.1 (19.0-40.2)	29.4 (16.4-44.7)	13.7 (5.4-26.0)	5.9 (1.3-15.0)
Cabo Verde	45.7 (37.0-54.4)	40.8 (29.3-52.9)	24.8 (15.9-35.4)	8.6 (3.8-15.1)
Cameroon	34.8 (23.9-46.6)	29.6 (17.4-43.9)	16.2 (7.1-28.5)	4.3 (0.7-11.6)
Chad	35.1 (21.5-50.1)	33.3 (15.7-53.6)	20.3 (7.3-39.4)	9.1 (1.6-23.7)
Cote d'Ivoire	36.9 (23.5-51.5)	31.7 (16.1-50.5)	20.3 (7.6-38.7)	6.4 (1.0-18.0)
Gambia	34.7 (23.2-47.4)	35.6 (20.4-52.8)	22.0 (10.2-37.2)	8.0 (1.7-19.9)
Ghana	31.4 (22.0-41.2)	37.8 (26.2-51.2)	27.6 (16.5-40.7)	13.5 (5.2-25.6)
Guinea	38.3 (25.2-53.0)	31.0 (16.3-48.0)	16.6 (6.2-31.7)	5.7 (0.9-16.9)
Guinea Bissau	36.0 (20.9-52.7)	34.1 (14.5-58.1)	21.2 (5.8-44.3)	8.6 (1.1-25.2)
Liberia	37.4 (26.2-49.5)	35.6 (20.0-52.7)	23.7 (10.5-41.0)	7.9 (1.7-19.4)
Mali	30.5 (18.5-44.0)	43.0 (24.9-62.9)	29.6 (12.6-50.0)	11.8 (2.2-29.6)
Mauritania	35.9 (21.8-52.4)	33.7 (14.2-57.5)	21.1 (5.5-44.4)	8.6 (1.2-24.5)
Niger	40.4 (27.0-54.3)	19.1 (7.9-34.6)	11.7 (3.6-25.0)	4.0 (0.5-12.6)
Nigeria	33.0 (25.7-40.8)	41.7 (30.3-53.9)	27.4 (18.0-38.7)	11.4 (5.5-19.5)
Sao Tome and Principe	41.9 (33.2-50.9)	36.0 (24.8-48.1)	19.3 (11.1-29.6)	6.9 (2.8-13.3)
Senegal	37.3 (24.5-51.0)	30.1 (14.9-48.0)	15.5 (5.6-30.7)	7.2 (1.5-18.1)
Sierra Leone	38.0 (25.1-51.8)	29.7 (15.5-46.8)	15.5 (5.8-29.8)	6.2 (1.1-16.6)
Togo	34.3 (22.2-47.2)	30.0 (16.0-47.5)	13.7 (4.6-27.1)	5.2 (0.8-15.0)

Appendix Table 4. Status of hypertension risk factors, and policies and programmes related to hypertension prevention, detection, treatment and control in seven countries with high hypertension treatment rates.

Country	Factors related to hypertension prevention*	Factors related to hypertension detection, treatment and control
Canada	- Mean BMI: 27 kg/m^2 in women and 27.5 kg/m^2 in men, an increase of 2-2.5 kg/m^2 since 1990.<br>- Average sodium intake: ~3.7 g/day.<br>- Availability of vegetables and of starchy roots and fruits increased since 1990.<br>- Smoking prevalence: 12% in women and 17% in men, a 40-50% relative reduction since 2000.	- The healthcare system is funded through personal and corporate taxes.<br>- 100% public insurance coverage for physician consultations and for drugs provided in hospitals. Payment needed for prescriptions. Private benefit plans cover varying proportions of prescriptions.<br>- National effort to improve hypertension control is coordinated by the Canadian Hypertension Education Program (CHEP), started in 1999. CHEP included three workforces on recommendation, implementation and outcome evaluation. Key features in CHEP include: annual update of guidelines; tailored knowledge dissemination; and coordinated implementation and outcome evaluation.<br>- Hypertension guidelines were first published in 1977 with annual update since 1999. Since 1993, guidelines recommended immediate treatment for people with BP ≥160/100 mmHg and recommended immediate treatment based on other risks for people with BP between 140/90 and 160/100 mmHg.
Costa Rica	- Mean BMI: 28 kg/m^2 in women and 27 kg/m^2 in men, an increase of 3.5 kg/m^2 in women and 2.5 kg/m^2 in men since 1990.<br>- Average sodium intake: ~3.2 g/day.<br>- Availability of vegetables and of starchy roots and fruits increased since 1990.<br>- Smoking prevalence: 6% in women and 17% in men, a ~30% relative reduction since 2000.	- Healthcare system is funded by 70% public resources, 27% out of pocket spending, and 3% private insurance in 2010. Government covers children and pregnant women not covered through other means, retried people and homeless people.<br>- Single-payer health system (CCSS) with universal lifetime coverage, serving 95% of the population and providing strong safety net for the poor. Low inequality in health service access and health outcomes nationally.<br>- A wide nationwide network of primary care facilities organised in a community-based clinic model (EBAIS). Hypertension prevention and detection is part of the planned care for adults and carried out in primary care.<br>- Antihypertensive medications are in the list of essential medicines. No co-payment for drugs accessed via the public sector.<br>- Evidence-based national guidelines for hypertension management that is adapted to local resources.
Cuba	- Mean BMI: 26 kg/m^2 in women and 25 kg/m^2 in men, an increase of 1.5-2 kg/m^2 since 1990.<br>- Average sodium intake: ~2.6 g/day.<br>- Availability of vegetables and that of starchy roots and fruits increased since 1990.<br>- Smoking prevalence: 17% in women and 53% in men, a 45% relative reduction in women and 5% in men since 2000.	- Publicly funded health care system with universal coverage. There is no private health care while out-of-pocket expenditures account for ~10% of total health expenditure.<br>- A comprehensive primary health care system has an extensive and well-organised network of primary health care facilities and plentiful workforce throughout the country. 80% of the care is provided at primary care level.<br>- Domestically produced generic antihypertensive medicines are available and prices are kept stable by subsidy from state budget.<br>- Primary-care based national hypertension programme implemented for more than four decades, with updated national clinical guidelines and affordable medications. The programme now

		includes task sharing among different health care professionals and a registry with performance reporting.<br>- The Cuba HEARTS programme was awarded the 2020 World Hypertension League Award.
Germany	- Mean BMI: 26 kg/m^2 in women and 27.5 kg/m^2 in men, an increase of ~1.5 kg/m^2 since 1990.<br>- Average sodium intake: ~3.5 g/day.<br>- Availability of vegetables increased, but availability of starchy roots and fruits decreased slightly since 1990.<br>- Smoking prevalence: 28% in women and 33% in men, a 13% relative reduction in women and 21% in men since 2000.	- 99.9% insurance coverage, with 88% public and 11% private. The healthcare system is funded through 78% compulsory insurance with equal contributions by employer and employee, 7% government schemes, 13% out of pocket and 1% voluntary health insurance.<br>- Free health check-ups, included BP screening, are available for those aged ≥35 years since 1989, and expanded to ≥18 years in 2019.<br>- Payment needed for outpatient prescriptions.<br>- Hypertension guidelines published by ESH/ESC have been used since 2003, which is updated every ~5 years. Since 2003, guidelines recommended immediate treatment for people with BP ≥180/110 mmHg, and treatment after lifestyle modification (unless high CVD risk) for people with BP between 140/90 and 180/110 mmHg. Since 2018, guidelines recommended treatment based on CVD risk for people with BP between 130/85 and 140/90 mmHg.
South Korea	- Mean BMI: 23 kg/m^2 in women and 24.5 kg/m^2 in men, an increase of 0.8 kg/m^2 for women and ~2 kg/m^2 for men, with trends largely flat for women since 2000.<br>- Average sodium intake: ~5.2 g/day.<br>- Availability of vegetables increased greatly, but availability of starchy roots and fruits decreased slightly since 1990.<br>- Smoking prevalence: 6% in women and 41% in men, a 13% relative reduction in women and 35% in men since 2000.	- The healthcare system is funded by 56.5% public resources, 6.7% private insurance and 36.8% out of pocket in 2014.<br>- National Health Insurance system increased coverage from ~25% in 1980 to ~100% in 1989.<br>- A 2000 pharmaceutical policy reform mandated separating drug prescription and dispensing, and increased physician contact.<br>- Co-payment is required for each prescription.<br>- National Health Screening programme started in 1995. It currently covers 97% of adult population and offers free biennial general health screening, including BP check. The participation rate of health screening was 75-80% in 2014-2019.<br>- In some regions, community-based hypertension and diabetes registration and management programmes have been launched since 2012 to improve diagnosis and treatment adherence.<br>- Hypertension guidelines are published by KSH since 2000, with updates every 4-9 years. Since 2004, guidelines recommended immediate treatment for people with BP ≥160/100 mmHg and recommended treatment after lifestyle modification for people with BP between 140/90 and 160/100 mmHg and low CVD risk (immediate treatment for those with medium to high CVD risk).
Taiwan	- Mean BMI: 24 kg/m^2 in women and 25 kg/m^2 in men, an increase of 1-2 kg/m^2 since 1990.<br>- Average sodium intake: ~3.9 g/day.<br>- Availability of vegetables increased greatly, and availability of starchy roots and fruits decreased slightly since 1990.<br>- Smoking prevalence: 3% in women and 19% in men, decreased by 1-3% per year since 1990.	- Single-payer national health insurance system provides universal health care with ~100% coverage since 1996. The costs for clinical visits, medications and monitoring are covered by public funds.<br>- National annual health check-up programmes for elderly population, including county-based integrated preventive service programmes.<br>- Antihypertensive medications can be easily prescribed by physicians and the cost is covered by the insurance systems, including generics and brand drugs.<br>- Academic and professional societies publish hypertensin guidelines since 2010 which are updated regularly, with good uptake among physicians and health professionals. Guidelines recommend treating people with BP ≥140/90 mmHg.

USA	- Mean BMI: 29 kg/m^2 in both men and women, an increase of ~2.5 kg/m^2 since 1990.<br>- Average sodium intake: ~3.6 g/day.<br>- Availability of vegetables and of starchy roots and fruits increased since 1990.<br>- Smoking prevalence: 19% in women and 25% in men, a ~30% relative reduction since 2000.	- Public and private insurance covers 80-90% of population. Healthcare is funded 48% by public sources, 40% by private insurance and 12% out of pocket.<br>- Co-payment is dependent on health insurance scheme and service.<br>- Good access to care in the population, although there is a socio-economic gradient.<br>- High awareness of hypertension in the public, through efforts of academic societies and national programmes over the past 3-4 decades, e.g., the National High Blood Pressure Education Program (NHBPEP) since 1972.<br>- Evidence-based, regularly updated hypertension guidelines. Guidelines recommended immediate treatment for people with BP ≥160/110 mmHg in 1993 and lowered threshold to 160/100 mmHg in 1997, regardless of other risks. Since 2003, guidelines recommended treating people with BP between 140/90 and 160/100 mmHg regardless of other risks but after lifestyle modification. Since 2017, guidelines recommended treating people with BP between 130/80 and 140/90 based on CVD risks.

*Latest data available are reported: BMI (2016);[17] sodium intake (2010);[19] availability of vegetables and availability of starchy roots and fruits for human consumption (2013);[20] tobacco smoking (2015/2016).[21,22]

BMI: body-mass index; BP: blood pressure; CVD: cardiovascular disease; NCD: noncommunicable disease.
CCSS: Caja Costarricense de Seguro Social; EBAIS: Equipos Básicos de Atención Integral de Salud; ESC: European Society of Cardiology; ESH: European Society of Hypertension; KSH: Korean Society of Hypertension.

Appendix Figure 1. Proportion of people with hypertension who reported a diagnosis, who used treatment, and whose blood pressure was effectively controlled, by region and age group in 2019.

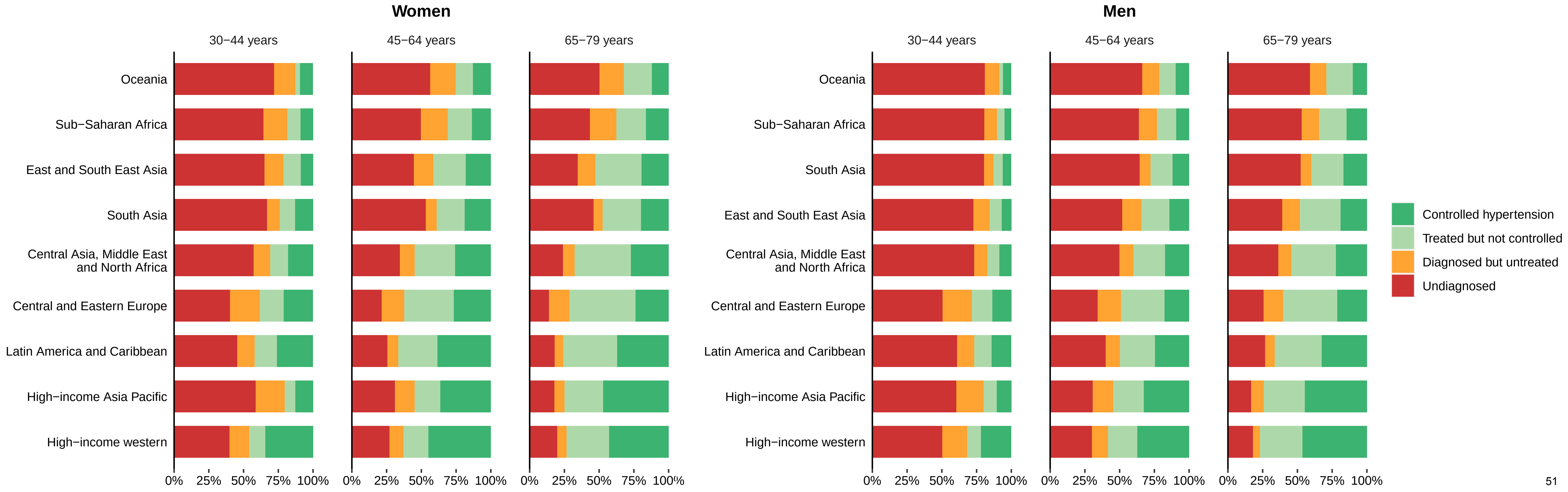
Women
Men
30−44 years
45−64 years
65−79 years
Oceania
Sub−Saharan Africa
East and South East Asia
South Asia
Central Asia, Middle East and North Africa
Central and Eastern Europe
Latin America and Caribbean
High−income Asia Pacific
High−income western
0%
25%
50%
75%
100%
Controlled hypertension
Treated but not controlled
Diagnosed but untreated
Undiagnosed

Appendix Figure 2. Proportion of people using hypertension treatment whose blood pressure was effectively controlled in 2019, and change from 1990 to 2019.

2019

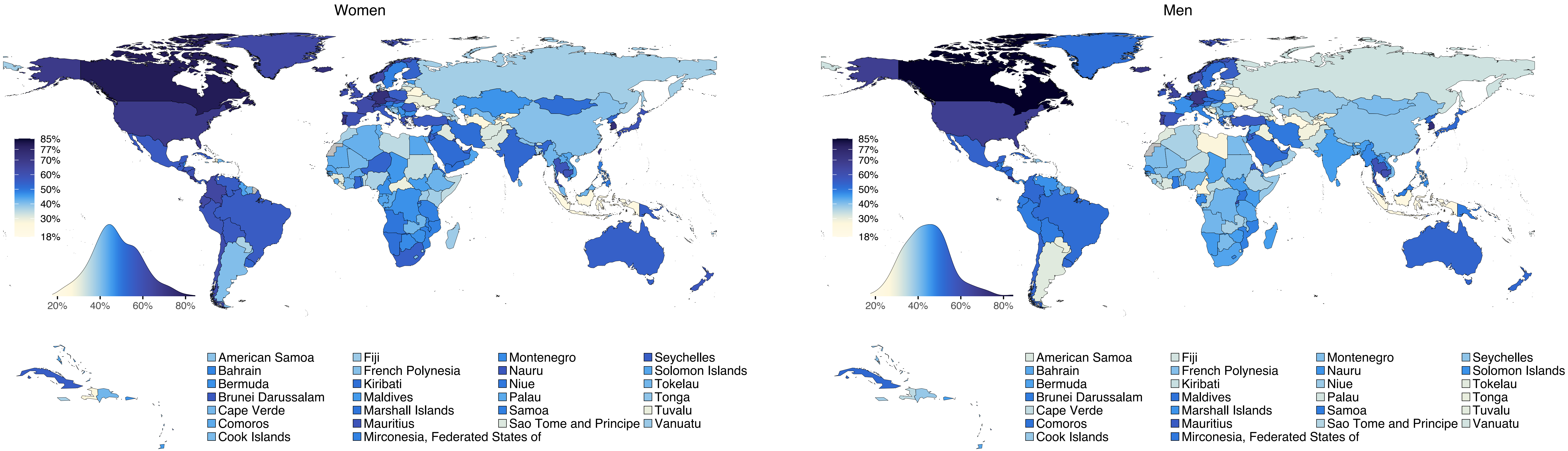

Change 1990-2019

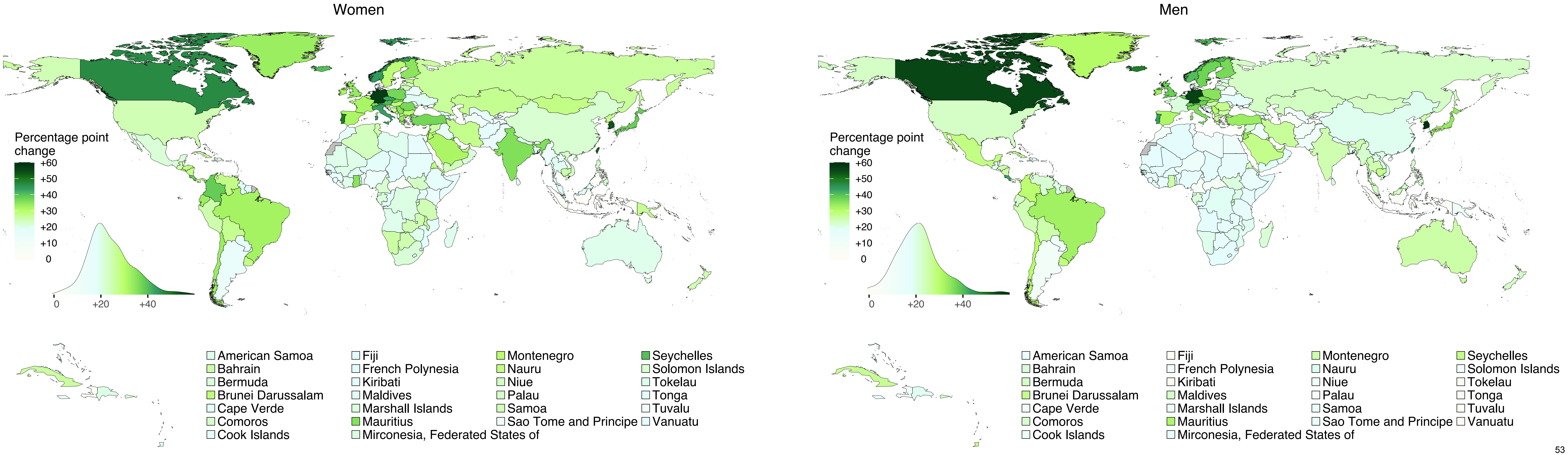

Appendix Figure 3. The relationship between the proportion of people with hypertension who had treatment and whose blood pressure was effectively controlled in 2019. Each point shows one country. The line represents the equality of treatment and control rates.

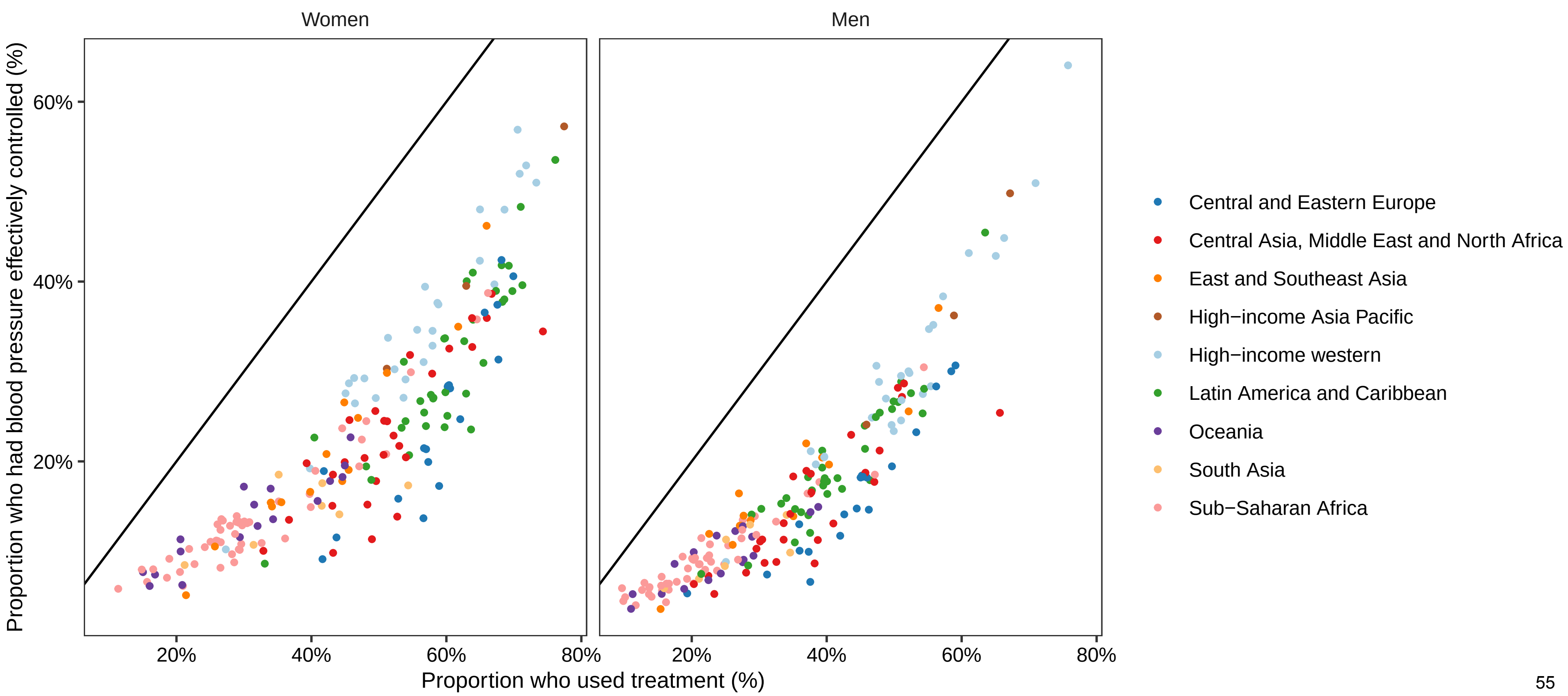

Women
Men
Proportion who had blood pressure effectively controlled (%)
60%
40%
20%
20%
40%
60%
80%
20%
40%
60%
80%
Proportion who used treatment (%)
Central and Eastern Europe
Central Asia, Middle East and North Africa
East and Southeast Asia
High−income Asia Pacific
High−income western
Latin America and Caribbean
Oceania
South Asia
Sub−Saharan Africa

Appendix Figure 4. The relationship between male and female hypertension prevalence, diagnosis, treatment and control in 2019. Each point shows one country.

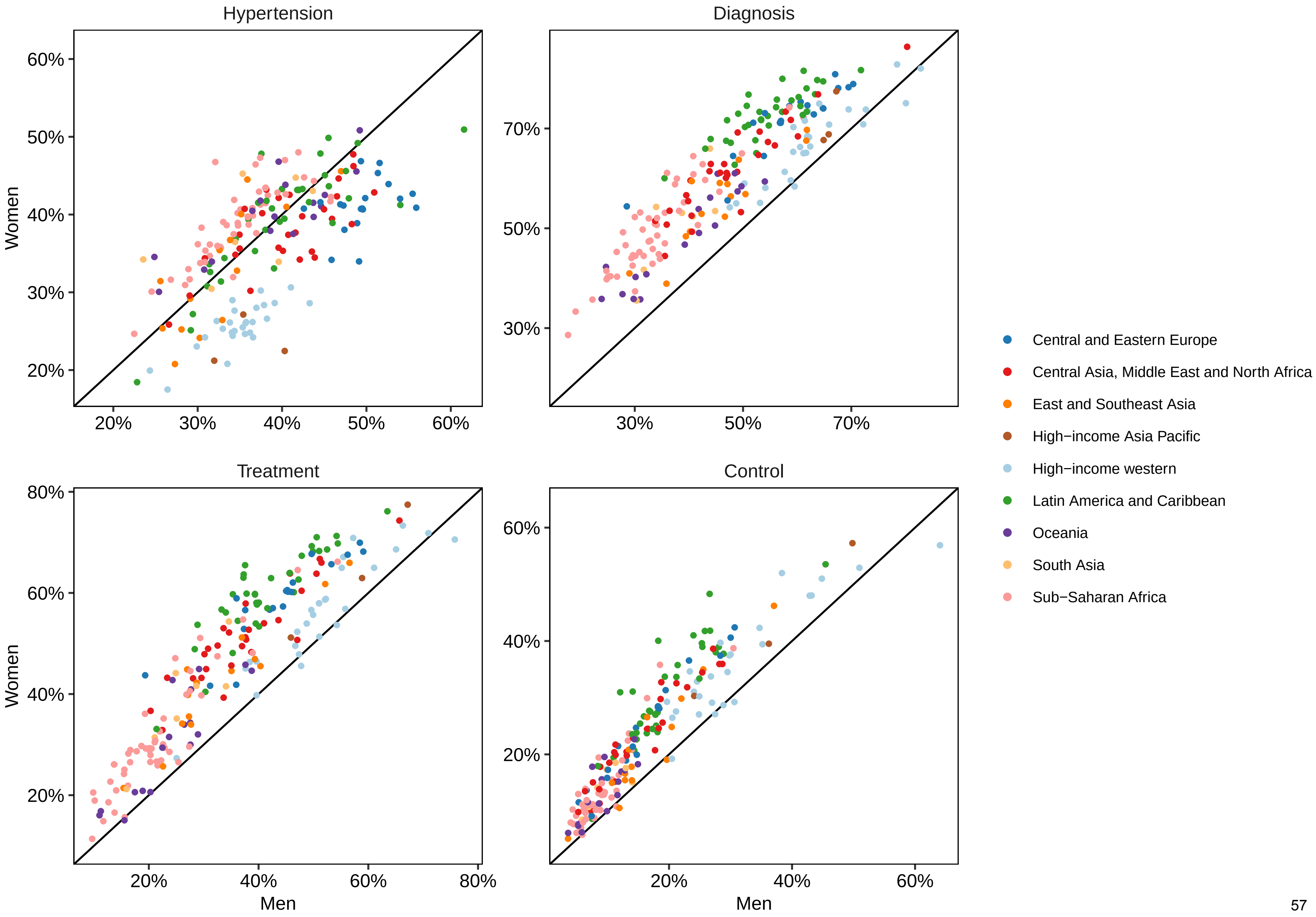

Hypertension
Diagnosis
Treatment
Control
Women
Men
20%
30%
40%
50%
60%
70%
80%
Central and Eastern Europe
Central Asia, Middle East and North Africa
East and Southeast Asia
High−income Asia Pacific
High−income western
Latin America and Caribbean
Oceania
South Asia
Sub−Saharan Africa

Appendix Figure 5. Trends in the proportion of people with hypertension who reported a diagnosis, who had treatment, and whose blood pressure was effectively controlled, globally and by region from 1990 to 2019.

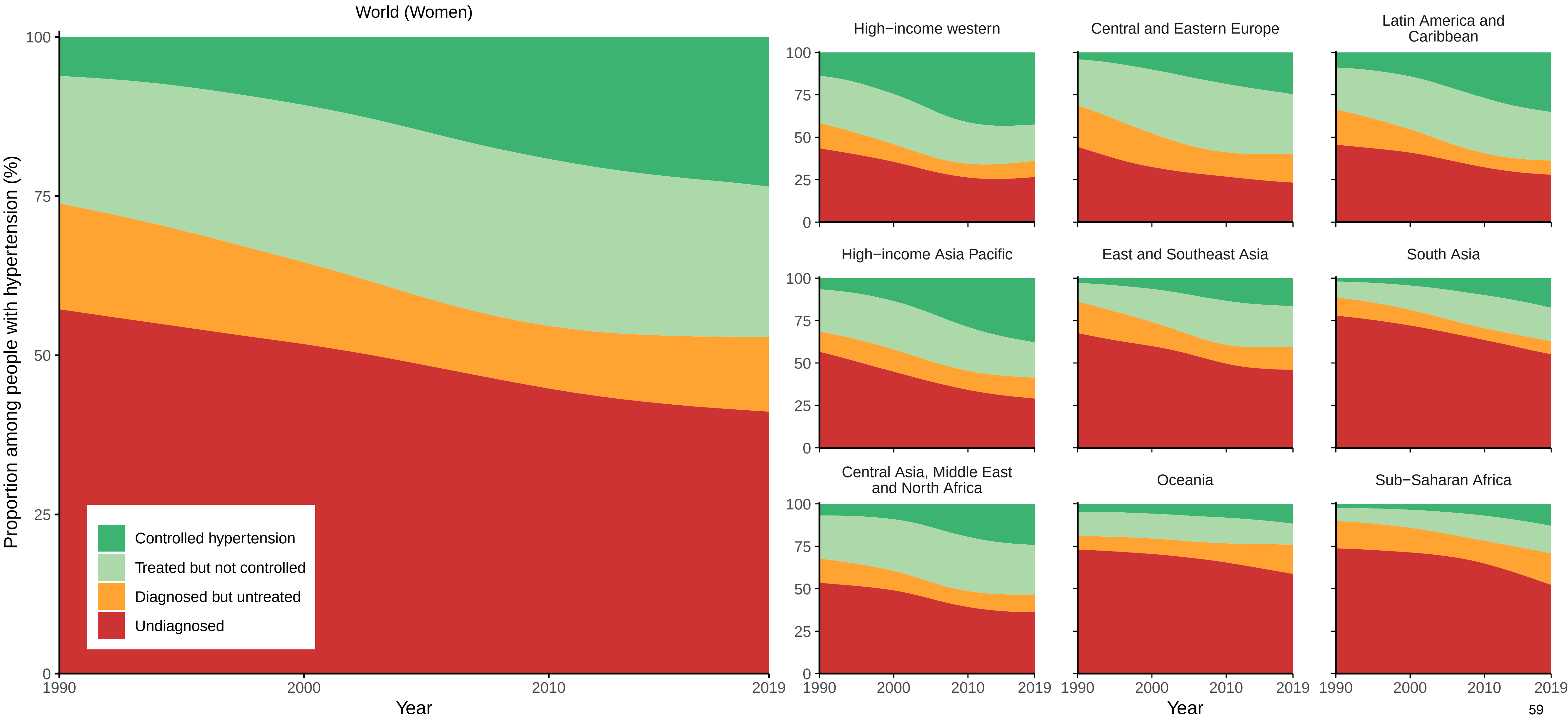
World (Women)
Proportion among people with hypertension (%)
100
75
50
25
0
1990
2000
2010
2019
Year
Controlled hypertension
Treated but not controlled
Diagnosed but untreated
Undiagnosed
High−income western
Central and Eastern Europe
Latin America and Caribbean
High−income Asia Pacific
East and Southeast Asia
South Asia
Central Asia, Middle East and North Africa
Oceania
Sub−Saharan Africa

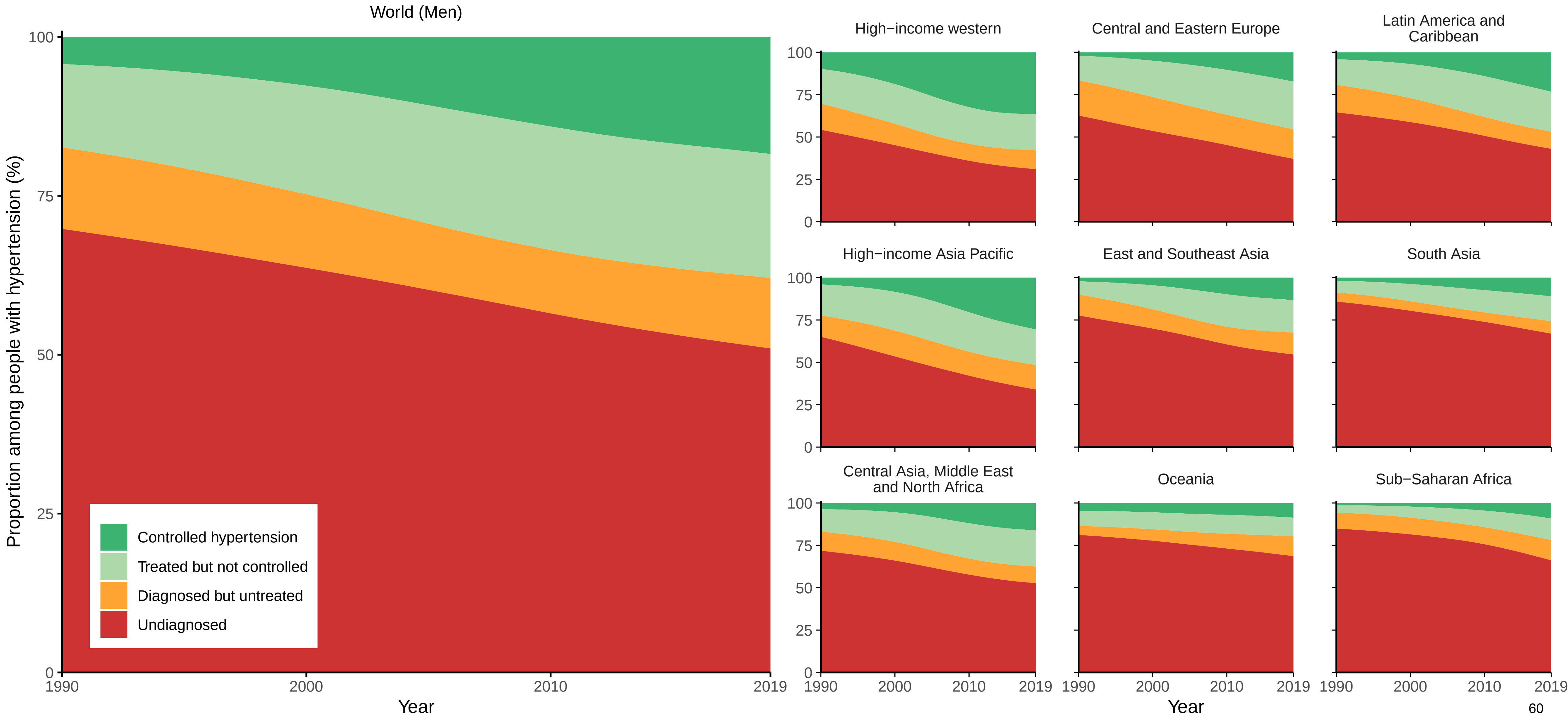
World (Men)
Proportion among people with hypertension (%)
Year
Controlled hypertension
Treated but not controlled
Diagnosed but untreated
Undiagnosed
High−income western
Central and Eastern Europe
Latin America and Caribbean
High−income Asia Pacific
East and Southeast Asia
South Asia
Central Asia, Middle East and North Africa
Oceania
Sub−Saharan Africa
0
25
50
75
100
1990
2000
2010
2019

Appendix Figure 6. Proportion of people with hypertension who reported a diagnosis in 2019, and change from 1990 to 2019.

The density plot alongside each map shows the distribution of estimates across countries. The top right panel shows the results ordered within regions and super-regions together with their 95% credible intervals. The bottom right panel shows change from 1990 to 2019 in hypertension detection rates in relation to the uncertainty of the change measured by posterior standard deviation; shaded areas show the posterior probability of an estimated increase or decrease being a true increase or decrease. Each point shows one country. See Appendix Table 3 for numerical results. SD: standard deviation. PP: posterior probability.

2019 (women)

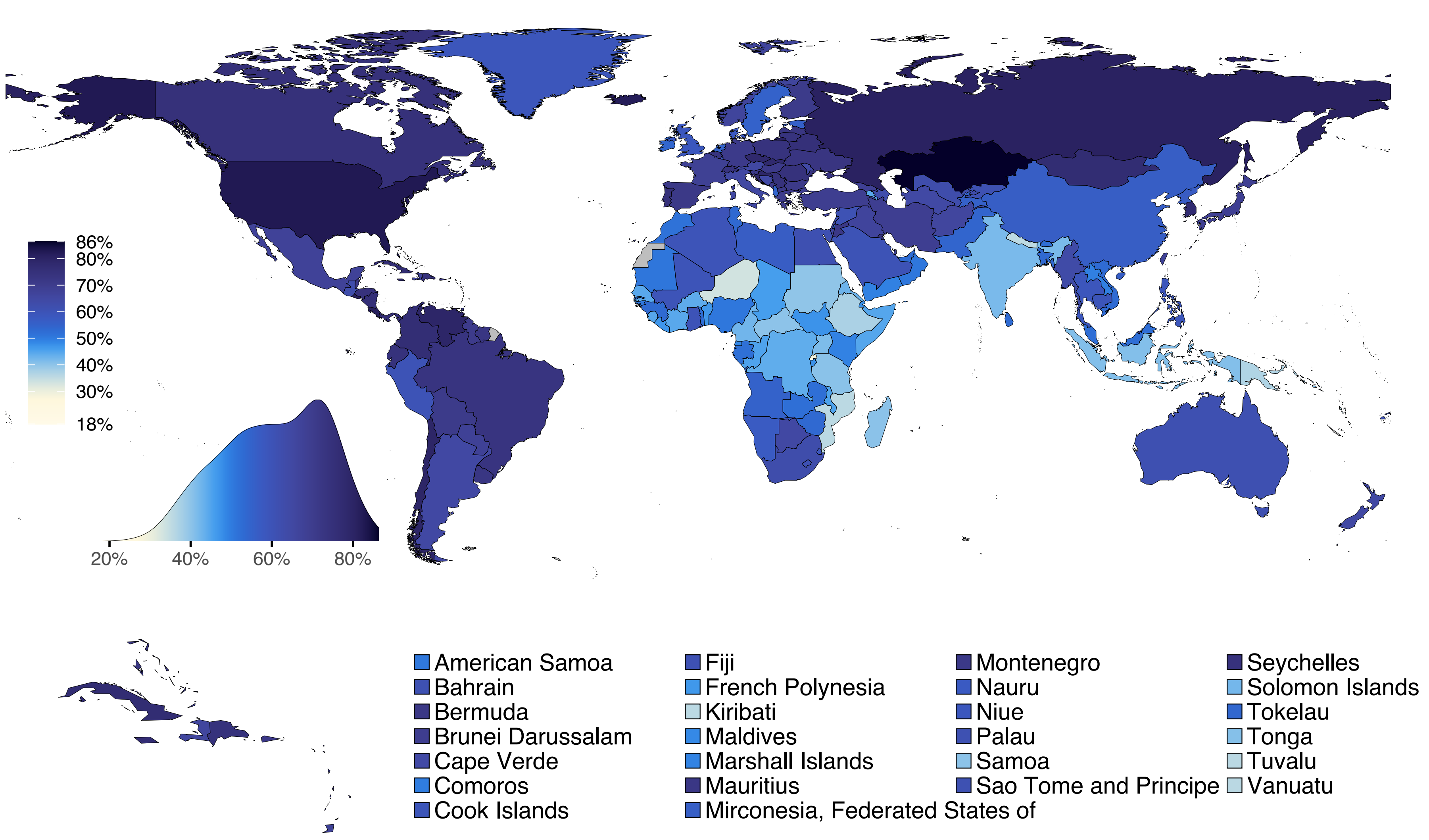

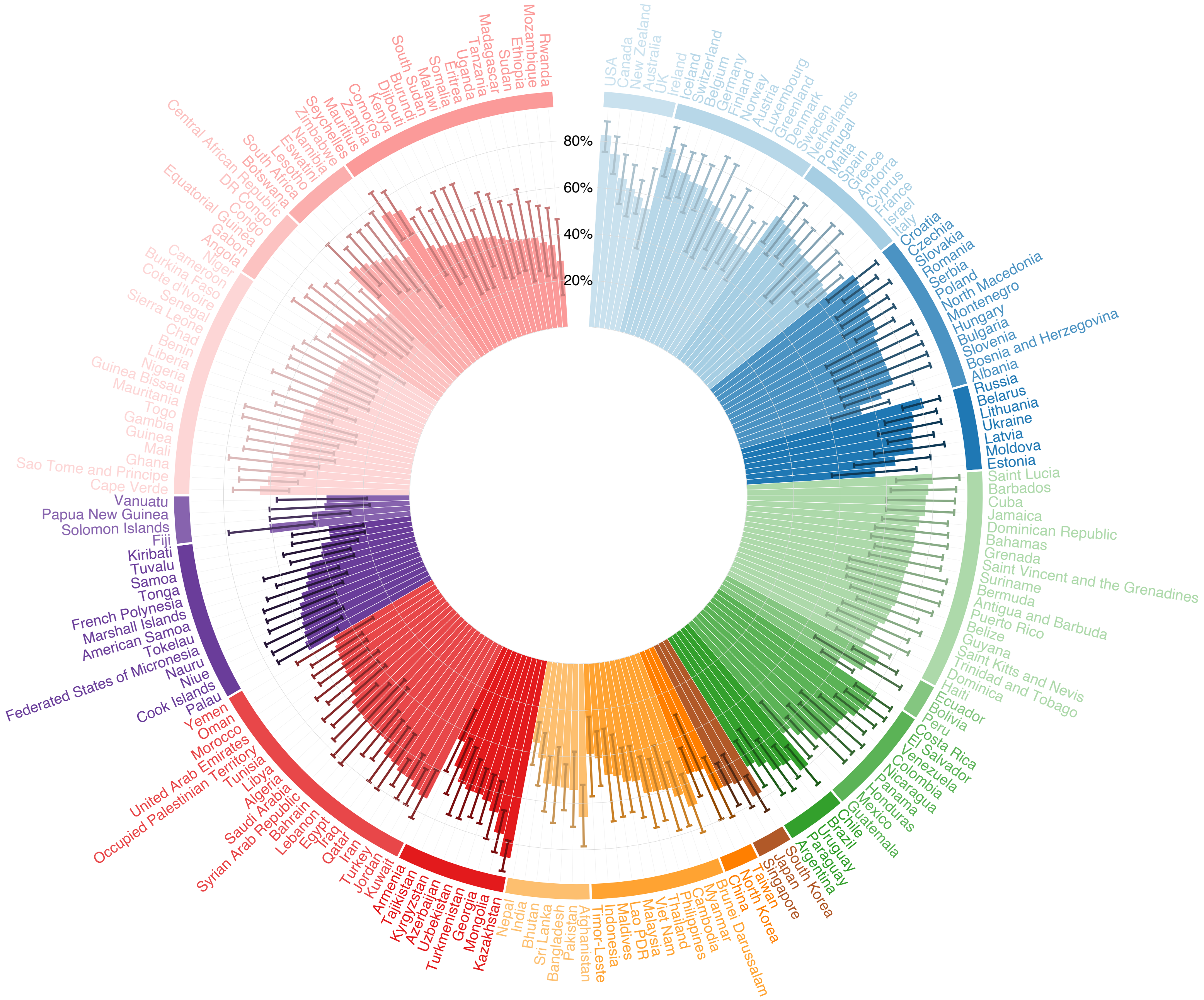

Change 1990-2019 (women)

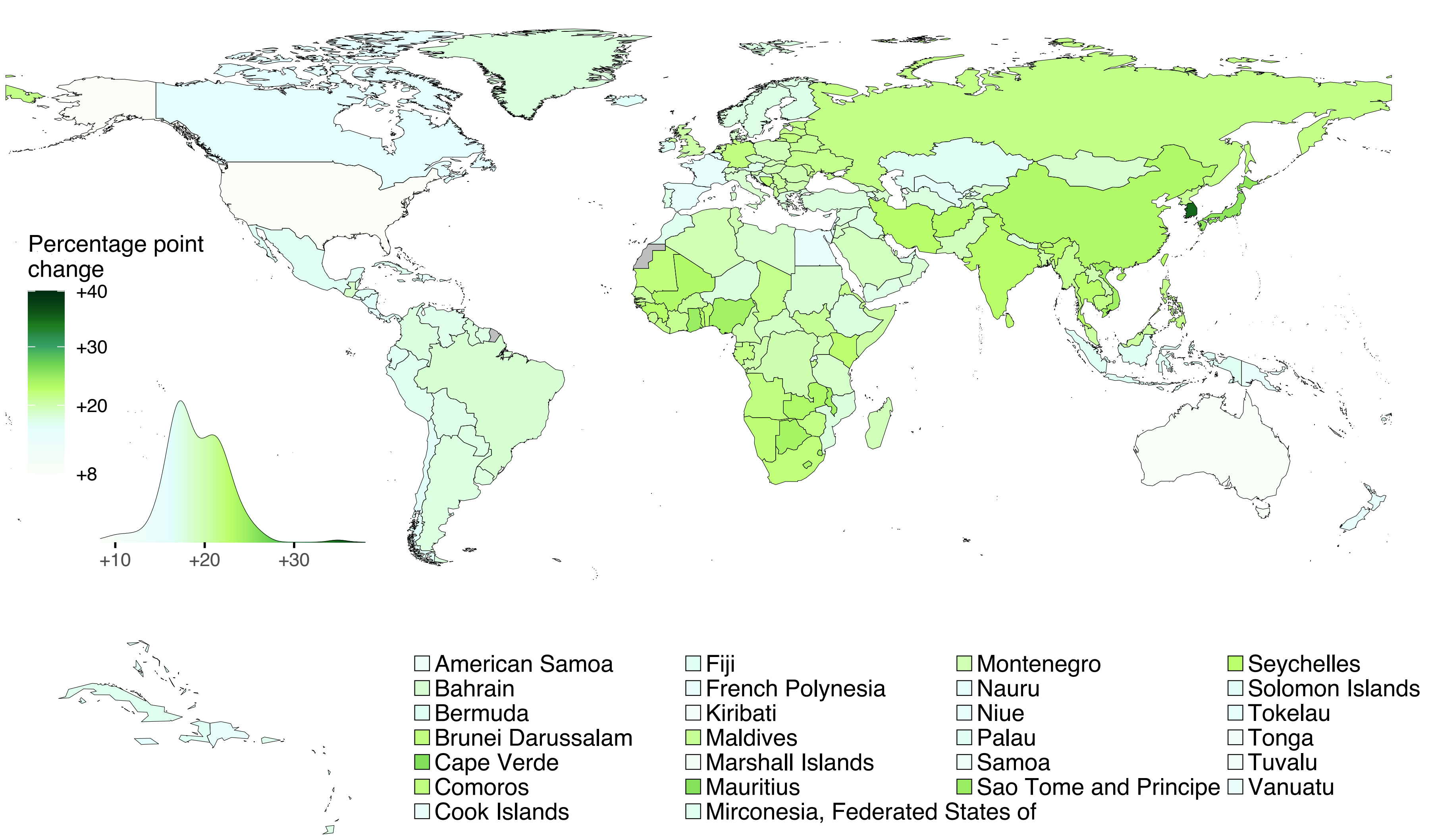

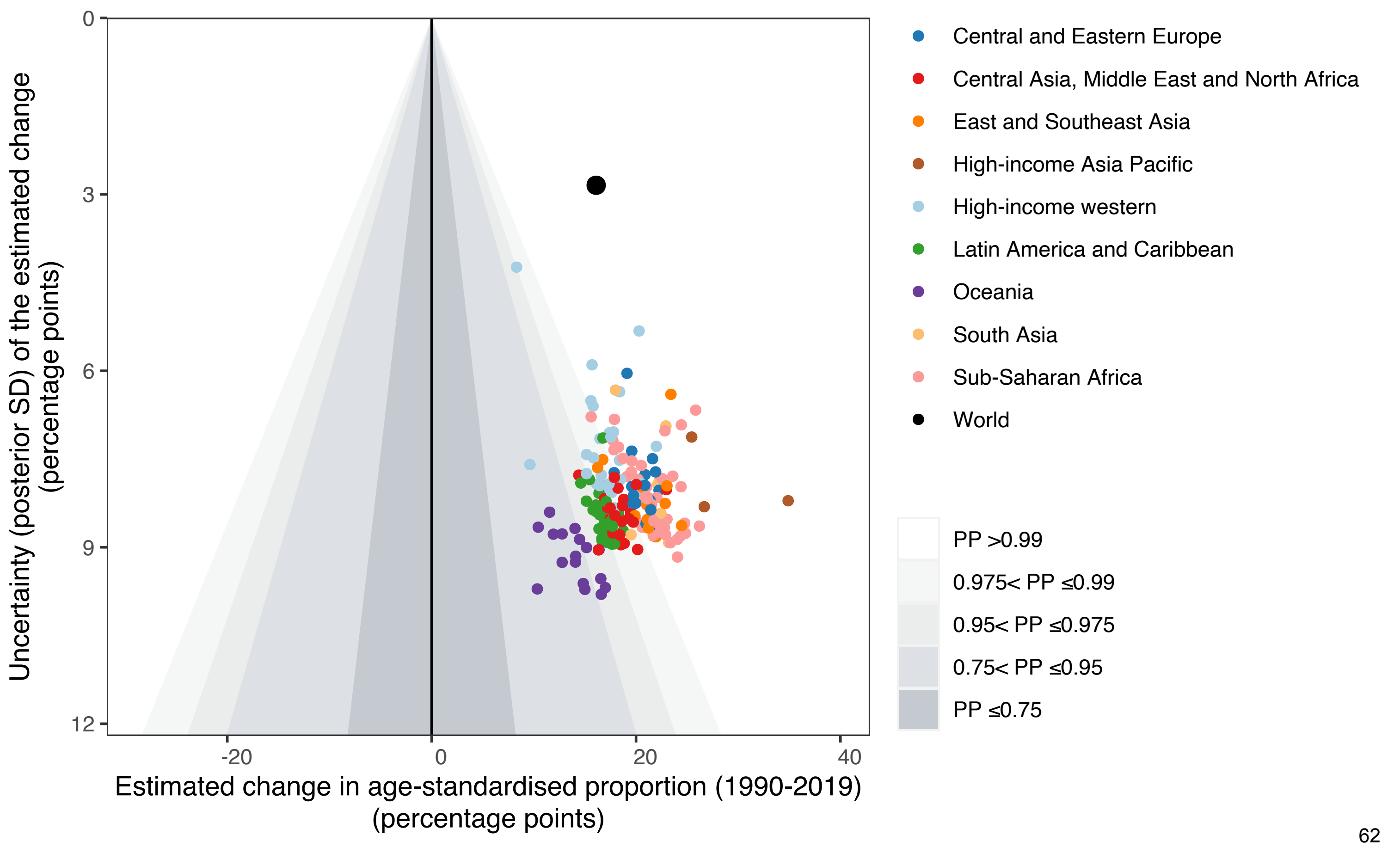

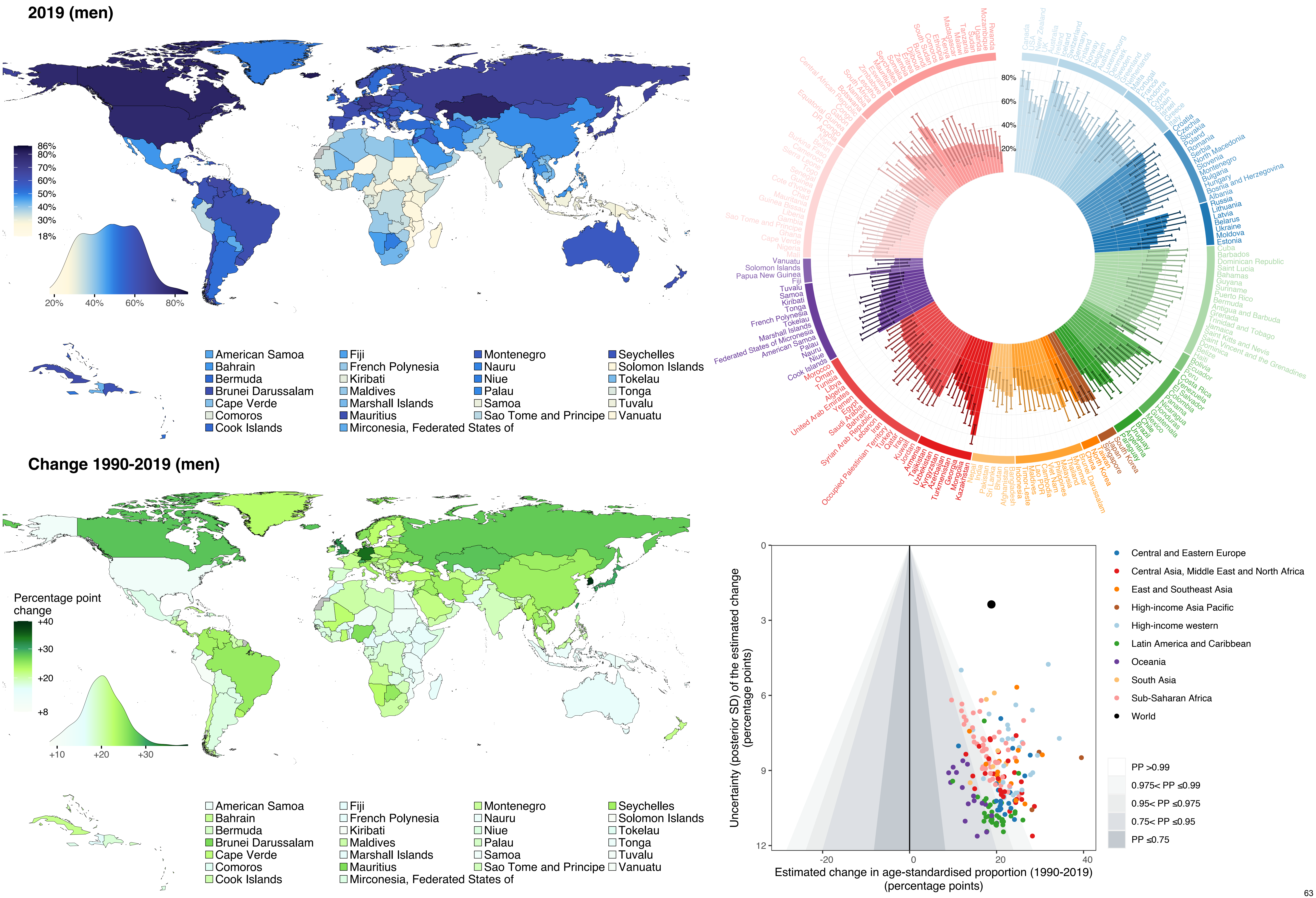
2019 (men)
86%
80%
70%
60%
50%
40%
30%
18%
20%
40%
60%
80%
American Samoa
Bahrain
Bermuda
Brunei Darussalam
Cape Verde
Comoros
Cook Islands
Fiji
French Polynesia
Kiribati
Maldives
Marshall Islands
Mauritius
Mirconesia, Federated States of
Montenegro
Nauru
Niue
Palau
Samoa
Sao Tome and Principe
Seychelles
Solomon Islands
Tokelau
Tonga
Tuvalu
Vanuatu
Change 1990-2019 (men)
Percentage point change
+40
+30
+20
+8
+10
+20
+30
American Samoa
Bahrain
Bermuda
Brunei Darussalam
Cape Verde
Comoros
Cook Islands
Fiji
French Polynesia
Kiribati
Maldives
Marshall Islands
Mauritius
Mirconesia, Federated States of
Montenegro
Nauru
Niue
Palau
Samoa
Sao Tome and Principe
Seychelles
Solomon Islands
Tokelau
Tonga
Tuvalu
Vanuatu
80%
60%
40%
20%
Uncertainty (posterior SD) of the estimated change (percentage points)
0
3
6
9
12
-20
0
20
40
Estimated change in age-standardised proportion (1990-2019) (percentage points)
Central and Eastern Europe
Central Asia, Middle East and North Africa
East and Southeast Asia
High-income Asia Pacific
High-income western
Latin America and Caribbean
Oceania
South Asia
Sub-Saharan Africa
World
PP >0.99
0.975< PP ≤0.99
0.95< PP ≤0.975
0.75< PP ≤0.95
PP ≤0.75

References

1. NCD Risk Factor Collaboration. Worldwide trends in blood pressure from 1975 to 2015: a pooled analysis of 1479 population-based measurement studies with 19.1 million participants. *Lancet* 2017; **389**: 37-55.
2. Danaei G, Finucane MM, Lin JK, et al. National, regional, and global trends in systolic blood pressure since 1980: systematic analysis of health examination surveys and epidemiological studies with 786 country-years and 5.4 million participants. *Lancet* 2011; **377**: 568-77.
3. Danaei G, Finucane MM, Lu Y, et al. National, regional, and global trends in fasting plasma glucose and diabetes prevalence since 1980: systematic analysis of health examination surveys and epidemiological studies with 370 country-years and 2.7 million participants. *Lancet* 2011; **378**: 31-40.
4. Farzadfar F, Finucane MM, Danaei G, et al. National, regional, and global trends in serum total cholesterol since 1980: systematic analysis of health examination surveys and epidemiological studies with 321 country-years and 3.0 million participants. *Lancet* 2011; **377**: 578-86.
5. Finucane MM, Stevens GA, Cowan MJ, et al. National, regional, and global trends in body-mass index since 1980: systematic analysis of health examination surveys and epidemiological studies with 960 country-years and 9.1 million participants. *Lancet* 2011; **377**: 557-67.
6. Finucane MM, Paciorek CJ, Danaei G, Ezzati M. Bayesian estimation of population-level trends in measures of health status. *Stat Sci* 2014; **29**: 18-25.
7. Rue H, Held L. Gaussian Markov Random Fields: Theory and Applications. Boca Raton: Chapman and Hall/CRC; 2005.
8. NCD Risk Factor Collaboration. Long-term and recent trends in hypertension awareness, treatment, and control in 12 high-income countries: an analysis of 123 nationally representative surveys. *Lancet* 2019; **394**: 639-51.
9. Zhang Y, Moran AE. Trends in the prevalence, awareness, treatment, and control of hypertension among young adults in the United States, 1999 to 2014. *Hypertension* 2017; **70**: 736-42.
10. United Nations, Department of Economic and Social Affairs, Population Division. World Urbanization Prospects: The 2018 Revision (ST/ESA/SER.A/420). New York: United Nations, 2018.
11. Gu Q, Burt VL, Paulose-Ram R, Dillon CF. Gender differences in hypertension treatment, drug utilization patterns, and blood pressure control among US adults with hypertension: data from the National Health and Nutrition Examination Survey 1999-2004. *Am J Hypertens* 2008; **21**: 789-98.
12. Choi HM, Kim HC, Kang DR. Sex differences in hypertension prevalence and control: Analysis of the 2010-2014 Korea National Health and Nutrition Examination Survey. *PLoS One* 2017; **12**: e0178334.
13. Rahman M, Williams G, Al Mamun A. Gender differences in hypertension awareness, antihypertensive use and blood pressure control in Bangladeshi adults: findings from a national cross-sectional survey. *J Health Popul Nutr* 2017; **36**: 23.
14. R Core Team. R: A language and environment for statistical computing. R Foundation for Statistical Computing, Vienna, Austria. 2020. https://www.R-project.org/.
15. NCD Risk Factor Collaboration. A century of trends in adult human height. *Elife* 2016; **5**: e13410.
16. NCD Risk Factor Collaboration. Worldwide trends in diabetes since 1980: a pooled analysis of 751 population-based studies with 4.4 million participants. *Lancet* 2016; **387**: 1513-30.
17. NCD Risk Factor Collaboration. Worldwide trends in body-mass index, underweight, overweight, and obesity from 1975 to 2016: a pooled analysis of 2416 population-based measurement studies in 128.9 million children, adolescents, and adults. *Lancet* 2017; **390**: 2627-42.

18. NCD Risk Factor Collaboration. Repositioning of the global epicentre of non-optimal cholesterol. *Nature* 2020; **582**: 73-7.
19. Powles J, Fahimi S, Micha R, et al. Global, regional and national sodium intakes in 1990 and 2010: a systematic analysis of 24 h urinary sodium excretion and dietary surveys worldwide. *BMJ Open* 2013; **3**: e003733.
20. Bentham J, Singh GM, Danaei G, et al. Multi-dimensional characterisation of global food supply from 1961-2013. *Nat Food* 2020; **1**: 70-5.
21. WHO global report on trends in prevalence of tobacco smoking 2000-2025, second edition. Geneva: World Health Organization, 2018.
22. GBD Tobacco Collaborators. Smoking prevalence and attributable disease burden in 195 countries and territories, 1990-2015: a systematic analysis from the Global Burden of Disease Study 2015. *Lancet* 2017; **389**: 1885-906.
